# Selective Para‐C−H Alkynylation of Aniline Derivatives by Pd/S,O‐Ligand Catalysis

**DOI:** 10.1002/chem.202104107

**Published:** 2022-01-12

**Authors:** Ke‐Zuan Deng, Wen‐Liang Jia, M. Ángeles Fernández‐Ibáñez

**Affiliations:** ^1^ Van't Hoff Institute for Molecular Sciences University of Amsterdam Science Park 904 1098 XH Amsterdam (The Netherlands

**Keywords:** aniline, alkynylation, C−H activation, palladium, S,O-ligand

## Abstract

Herein, we report a nondirected *para*‐selective C−H alkynylation of aniline derivatives by a Pd/S,O‐ligand‐based catalyst. The reaction proceeds under mild conditions and is compatible with a variety of substituted anilines. The scalability and further derivatizations of the alkynylated products have been also demonstrated.

## Graphical abstract

***para*** **‐Alkynylated** aromatic amines are obtained without the need to prefunctionalize the starting aniline by Pd/S,O‐ligand catalyzed C−H alkynylation reactions.

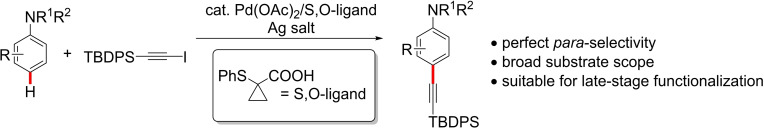

## Introduction

**Scheme 1:**
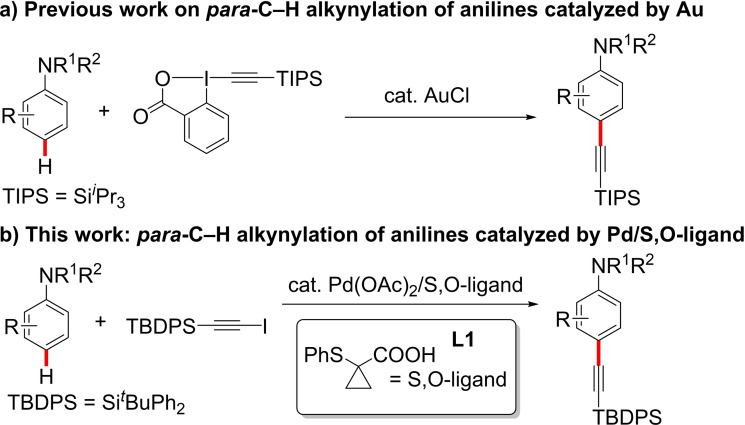
*para*‐C−H alkynylation of anilines.

The alkyne motif is a key functional group in organic chemistry due to its synthetic versatility.^[1]^ In addition, it is widely present in pharmaceuticals,^[2]^ biomolecules,[^1f^, ^3^] natural products,^[4]^ and materials.^[5]^ Therefore, the development of methodologies that allow the introduction of this functional group is of great interest in synthesis. In this regard, one of the most common methods to obtain arylacetylenes is the Sonogashira coupling.^[6]^ Despite its popularity, this reaction suffers from the disadvantage of requiring prefunctionalized aryl halides. In the last decade, metal‐catalyzed C−H functionalization has emerged as a greener alternative to traditional cross coupling since no prefuntionalization of starting materials is required. In this regard, metal‐catalyzed C−H alkynylation reactions of arenes have been widely described. However, the majority of the reported methodologies rely on the use of directing groups (DGs),^[7]^ leading in most of the cases to the *ortho*‐alkynylated products.^[8]^ Recently, a breakthrough was reported by the group of van Gemmeren on the Pd‐catalyzed C−H alkynylation of non‐directed arenes enabled by a dual ligand system.^[9]^ However, the C−H alkynylation of anilines, which are ubiquitous structural moieties in organic molecules, was not disclosed. As far as we know, only one report on the *para*‐C−H alkynylation of anilines using Au catalyst has been reported by the Waser group (Scheme 1a).^[10]^ Hence, the development of new general methodologies that permit the remote C−H alkynylation of aniline derivatives is still needed. Herein, we report an efficient *para*‐selective C−H alkynylation of a wide range of aniline derivatives by Pd/S,O‐ligand based catalyst (Scheme 1b).

Recently, we discovered a new type of S,O‐ligands, namely thioethercarboxylic acids, that enables Pd‐catalyzed C−H olefination of simple arenes, thiophenes, anisole and aniline derivatives.^[11]^ So far, only the introduction of an olefin moiety was achieved using the Pd/S,O‐ligand catalyst. However, taking into account the need to develop new methodologies for the remote C−H alkynylation of aniline derivatives, we decided to investigate if the Pd/S,O‐ligand catalyst was also suitable for the introduction of alkyne functionalities.

## Results and Discussion

We started our investigations using *N*,*N*‐dibenzylaniline **1 a** and 1‐iodo‐2‐(triisopropylsilyl)acetylene as model substrates in the presence of Pd(OAc)_2_, 3‐methyl‐2‐(phenylthio)butanoic acid as S,O‐ligand and AgOAc using a sealed tube. After an extensive screening of reaction conditions including solvents, silver salts, temperatures, Pd and alkyne sources and S,O‐ligands, optimal results were obtained using 1‐iodo‐2‐(*tert*‐butyldiphenylsilyl)acetylene (2 equiv) as alkyne source, Pd(OAc)_2_/S,O‐ligand **L1** (10 mol%) as catalyst, in the presence of AgOAc (2 equiv)^[12]^ in chloroform (0.2 M) at 80 °C (see Supporting Information). Under these conditions, we obtained the desired *para*‐alkynylated product **2 a** in 58 % isolated yield. It is worth mentioning that the reaction proceeds with perfect *para*‐selectivity as no other regioisomers were detected in the crude mixture.^[13]^

With the optimal alkynylation conditions in hand, we explored the C−H alkynylation reaction using different substituted anilines (Table 1). First, we evaluated *meta*‐halogenated *N,N*‐dibenzylanilines. The reaction of *N*,*N*‐dibenzyl‐3‐fluoroaniline (**1 b**) with 1‐iodo‐2‐(*tert*‐butyldiphenylsilyl)acetylene provided the desired product **2 b** in 69 % isolated yield with perfect *para*‐selectivity. Replacing the TBDPS protecting group by TIPS gave the product **2 c** in 62 % yield. When 3‐chloro‐ and 3‐bromo‐anilines were used, 58 % isolated yield of the desired products **2 d** and **2 e** were obtained. Then, when the reaction was performed with 3‐methyl‐ methoxy and ‐phenoxy *N*,*N*‐dibenzyl anilines **1 f**–**h**, the *para*‐alkynylated products **2 f**–**h** were isolated in synthetically useful yields (42–69 %). In these reactions, some decomposition of the starting or/and final anilines was observed, especially for the aniline bearing the methoxy group. Next, we switched our attention to *ortho*‐substituted anilines. Based on our previous experience that only *ortho*‐substituted secondary anilines are suitable substrates in these C−H functionalization reactions,^[11d]^ we performed the reactions using *N*‐benzyl anilines. We observed that *ortho*‐substituted anilines bearing electron donating substituents were prone to decomposition under the reaction conditions used. Nevertheless, to our delight, the less reactive *o*‐CO_2_Me‐substituted *N*‐benzyl aniline **1 i** provided the desired product in 43 % isolated yield with perfect *para*‐selectivity. Then, we evaluated a variety of disubstituted *N*‐benzyl anilines. Disubstituted anilines with an *ortho*‐methyl ester group and Me−, −OMe and −F substituent at the *meta*‐position underwent C−H alkynylation to provide only the *para*‐alkynylated products **2 j**–**2 l** in synthetically useful yields (37–50 %). *N*‐Benzyl‐*o*‐chloro‐*m*‐(methoxy)aniline (**1 m**) was also a suitable substrate providing the *para*‐alkynylated product in 40 % yield. To highlight the key role of the S,O‐ligand in this transformation, we performed the C−H alkynylation in the absence of the S,O‐ligand **L1** for each substrate (Table 1). As expected, no alkynylated or traces amount of product were obtained, highlighting the crucial role of the S,O‐ligand in the reaction. In comparison with the previously reported methodology by Waser et.al, this methodology is clearly more suitable for anilines bearing electron withdrawing groups while higher yields are obtained with anilines bearing electron donating substituents using Waser's methodology. Next, we proved the practicability of the reaction by performing the C−H alkynylation of *N,N*‐dibenzyl‐3‐fluoroaniline (**1 b**) on a 3.0 mmol scale. Under standard reaction conditions, the *para*‐alkynylated product **2 b** was obtained in 76 % isolated yield.

**Table 1:**
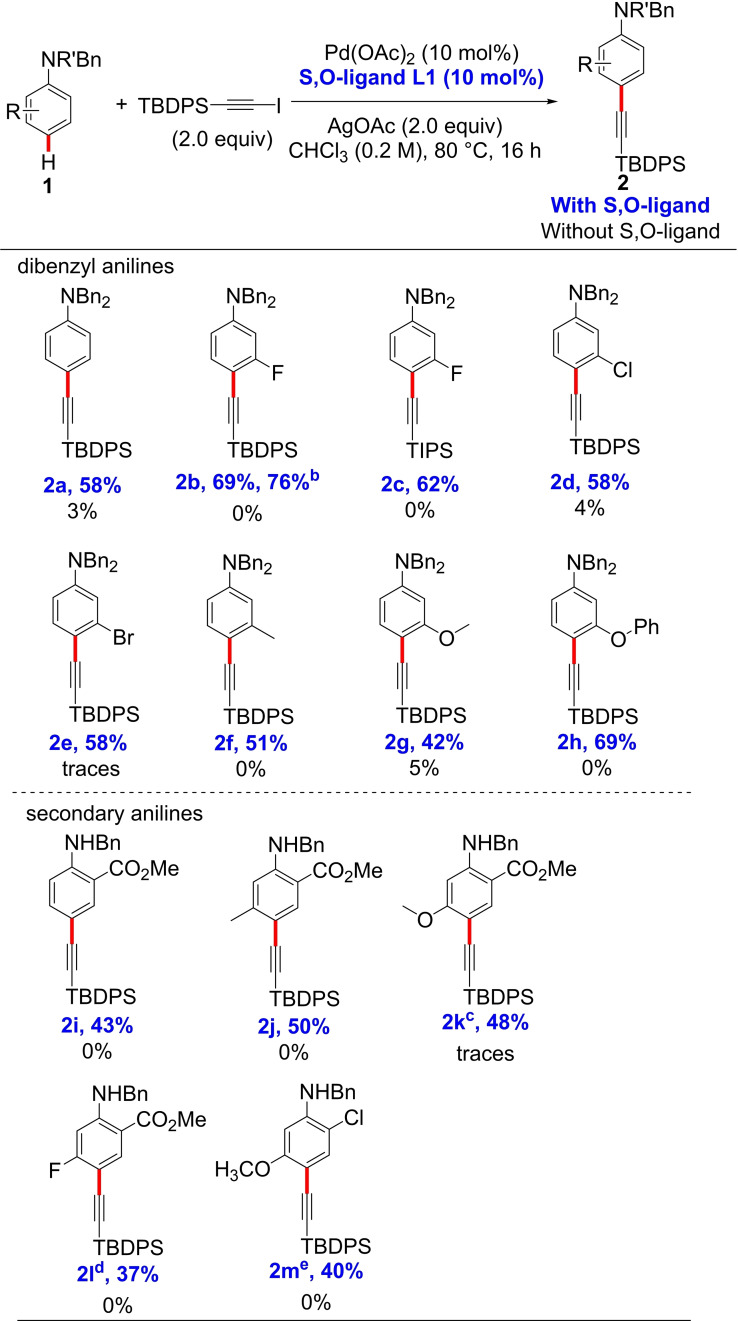
*para*‐C−H alkynylation of N‐benzylaniline.^[a]^

[a] ^1^H NMR yields of parallel reactions without ligands are given in black. ^1^H NMR yields were determined from the crude mixtures using CH_2_Br_2_ as an internal standard. [b] 3.0 mmol scale. [c] 1.0 equiv of alkyne source and AgOAc and 2.0 equiv of aniline substrate were used. [d] 3.0 equiv of alkyne source and AgOAc were used. [e] 1.0 equiv of alkyne source and AgOAc and 2.0 equiv of aniline substrate were used at 100 °C.

After proving the efficiency of the Pd/S,O‐ligand catalyst in anilines bearing both electron donating and withdrawing groups, we studied the effect on the reaction of different substituents attached to the nitrogen atom (Table 2). 3‐Fluoro *N*,*N*‐dimethyl‐ and *N*,*N*‐diethylaniline (**1 n**–**1 o**) were alkynylated in 57 % and 68 % isolated yield, respectively. Interestingly, only the *para*‐alkynylation of the 3‐fluorophenyl ring when using 3‐fluoro‐*N*‐methyl‐*N*‐phenylaniline (**1 p**) was detected, showing the beneficial effect of the fluorine substituent in the reaction. The reaction of *N*‐(3‐fluorophenyl)pyrrolidine (**1 q**) and *N*‐(3‐fluorophenyl)morpholine (**1 r**) furnished the *para*‐alkynylated products in synthetically useful yields (42–51 %). Next, we performed the reaction with the unprotected 8‐acetyltetrahydroquinoline (**1 s**) and the bioactive spiro‐ tetrahydroquinoline derivative **1 t**.^[14]^ To our delight, both substrates were compatible with the reaction conditions, providing the alkynylated products in 49 % and 50 % isolated yield, respectively.

**Table 2:**
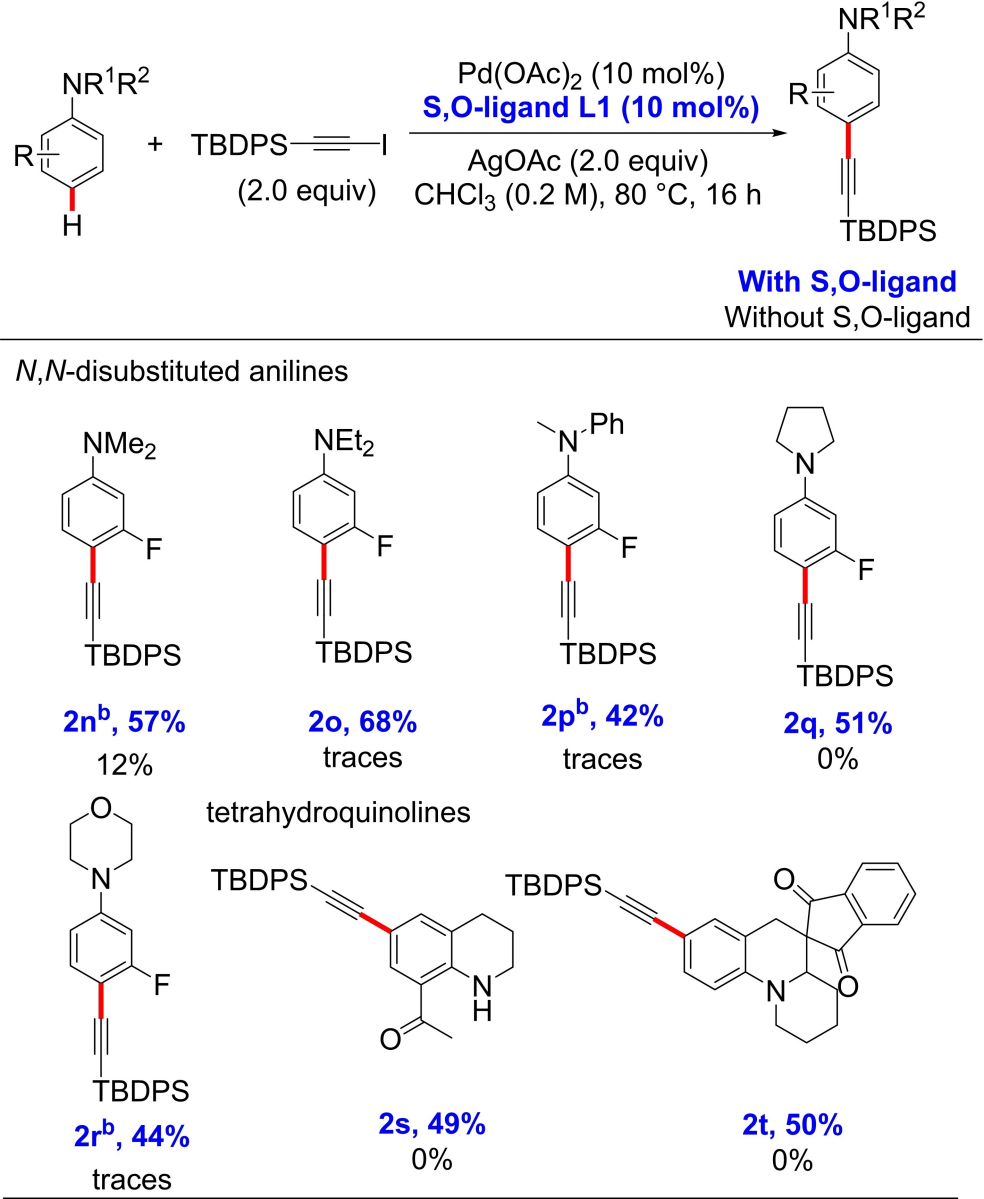
*para*‐C−H alkynylation of *N*,*N*‐disubstituted anilines and tetrahydroquinolines.^[a]^

[a] ^1^H NMR yields of parallel reactions without ligands are given in black. ^1^H NMR yields were determined from the crude mixtures using CH_2_Br_2_ as an internal standard. [b] 1.0 equiv of alkyne source, 1.0 equiv of AgOAc and 2.0 equiv of aniline substrate were used.

**Scheme 2:**
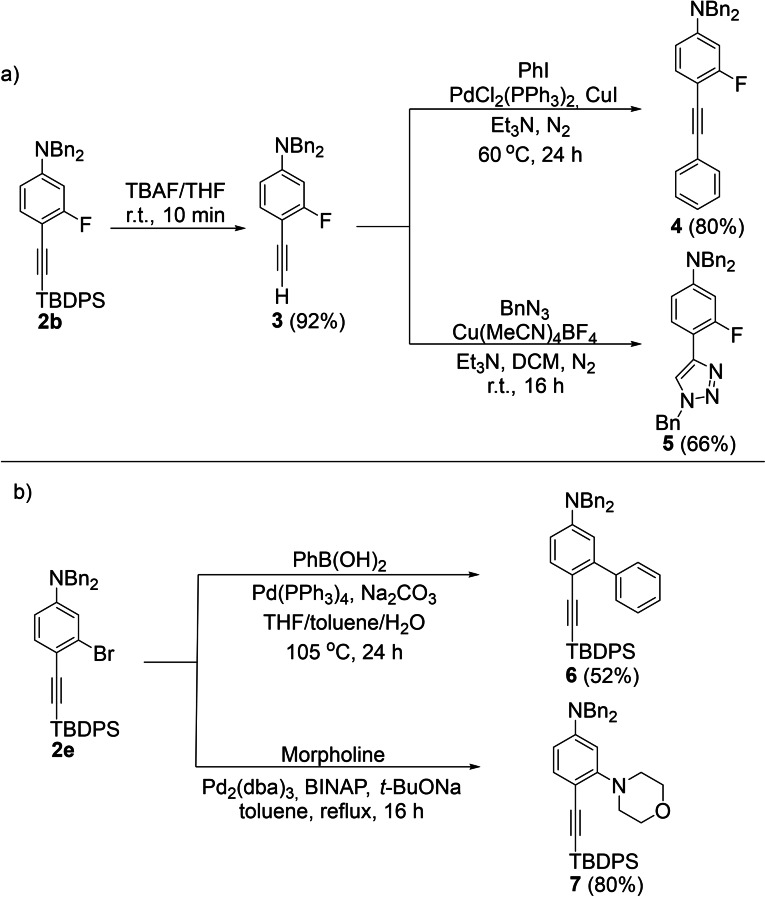
Synthetic derivatizations of the alkynylated products.

Finally, we proved the synthetic utility of the alkynylated products obtained via Pd‐catalyzed C−H alkynylation reactions as building blocks by performing further derivatization reactions (Scheme 2). The deprotection of **2 b** was conducted using TBAF/THF providing the desilylation product **3** in an excellent yield of 92 % (Scheme 2a). The treatment of the obtained terminal alkyne **3** with iodobenzene under typical Sonogashira coupling conditions led to diphenylethyne **4** in 80 % yield. Moreover, the terminal alkyne **3** was subjected to a click reaction to provide the triazole **5** in 66 % yield. To further prove the synthetic value of our methodology, we performed the derivatization of the *m*‐bromo substituted alkynylated aniline **2 e**. Thus, the coupling of **2 e** with PhB(OH)_2_ and morpholine using a Pd catalyst, furnished the coupling products in 52 % and 80 % isolated yield, respectively (Scheme 2b).

## Conclusion

In conclusion, we have developed a general protocol for nondirected *para*‐selective C−H alkynylation of aniline derivatives by Pd/S,O‐ligand catalysis. The reaction proceeds under mild conditions with perfect *para*‐selectivity providing the alkynylated anilines in synthetically useful yields. A wide number of *ortho* and *meta* substituted anilines bearing both electron donating and withdrawing substituents are compatible with the catalytic system. Anilines with different substituents attached to the nitrogen atom as well as tetrahydroquinolines are alkynylated in good yields. The methodology is operationally simple and scalable. The synthetic versatility of the functionalized products was shown by further derivatization of the alkynylated anilines.

## Conflict of interest

The authors declare no conflict of interest.

1

## Supporting information

As a service to our authors and readers, this journal provides supporting information supplied by the authors. Such materials are peer reviewed and may be re‐organized for online delivery, but are not copy‐edited or typeset. Technical support issues arising from supporting information (other than missing files) should be addressed to the authors.

Supporting Information

## Data Availability

The data that support the findings of this study are available in the supplementary material of this article.
