# Supplementary material for: Selective Para‐C−H Alkynylation of Aniline Derivatives by Pd/S,O‐Ligand Catalysis
Source: Chemistry. 2022 Jan 12;28(9):e202104107. doi: 10.1002/chem.202104107 (PMC9306564; doi:10.1002/chem.202104107)
Supplement: Supplementary file 1 — Supporting Information [file CHEM-28-0-s001.pdf]

Chemistry–A European Journal

Supporting Information

Selective Para-C–H Alkynylation of Aniline Derivatives by Pd/S,O-Ligand Catalysis

Ke-Zuan Deng, Wen-Liang Jia, and M. Ángeles Fernández-Ibáñez*

Table of Contents

1. General Information S2

2. Reaction optimization for C-H alkynylation of aniline derivatives S2

3. Synthesis of aniline substrates S5

4. Synthesis of alkyne sources S9

5. C–H alkynylation of aniline derivatives via Pd/S,O-ligand catalysis S11

6. Derivatization of alkynylated products S18

7. ^{1}H NMR and ^{13}C NMR spectra: S21

8. References.. S52

1. General Information

Chromatography: Silicycle Silica Flash P60 size 40-63 µm (230-400 mesh), TLC: Merck silica gel 60 (0.25mm), preparative TLC: Analtech silica gel G 1500 um 20x20 cm. Visualization of the chromatogram was performed by UV and $KMnO_4$ solution. ^1H and ^{13}C were recorded on Bruker 500 AMX, 400 and Bruker DRX 300 using $CDCl_3$ as the solvent otherwise it will be noted. Data are reported as follows: chemical shifts, multiplicity (s = singlet, d = doublet, dd = doublet of doublets, t = triplet, q = quartet, m = multiplet), coupling constants (Hz), and integration. IR spectra were recorded on a Bruker Alpha FTIR machine and wavelengths are reported in cm^{-1}. Mass spectra were recorded on a JEOL JMS-T100LP AccuTOF 2012 (ESI), JEOL JMS-T100GCv AccuTOF 2012, (EI, FD). The melting point was measured in Melting Point apparatus Büchi M-565. All reagents and solvents were used as received unless it was specified. $Pd(OAc)_2$ was purchased from Strem. *N*,*N*-dibenzylaniline (**1a**) were purchased from commercial suppliers. Aniline substrates **1i-1n** were prepared following the procedure reported in the literature.[1] Aniline substrates **1s** and **1t** were synthesized following the procedure described in the literature.[2] S,O-ligands were prepared following the procedure reported in the literature.[3]

2. Reaction optimization for C–H alkynylation of aniline derivatives

Table S1. Screening of stoichiometry of AgOAc and alkyne source. [a,b]

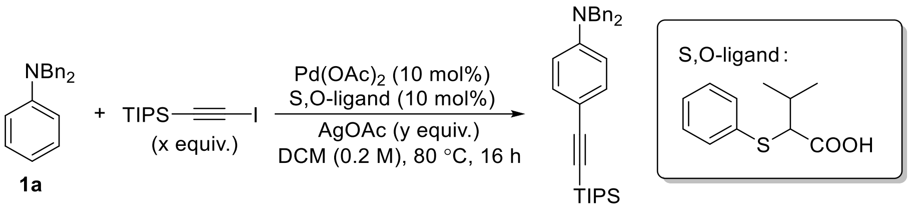

Entry	x/equiv.	y/equiv.	Conversion	^1H NMR yield of alkynylated product
1	1.0	2.0	66%	17%
2	3.0	2.0	53%	34%
3	2.0	1.0	38%	31%
**4**	**2.0**	**2.0**	**58%**	**39%**
5	2.0	3.0	77%	26%

[a]Reaction conditions: **1a** (27.3 mg, 0.1 mmol, 1.0 equiv.), $Pd(OAc)_2$ (2.2 mg, 0.01 mmol, 10 mol%), S,O-ligand (2.1 mg, 0.01 mmol, 10 mol%), CH_2Cl_2 (0.5 mL), 80 °C, 16 h. [b]The yields were determined by ^1H NMR analysis of crude reaction samples using CH_2Br_2 as internal standard.

Table S2. Screening of solvents. [a,b]

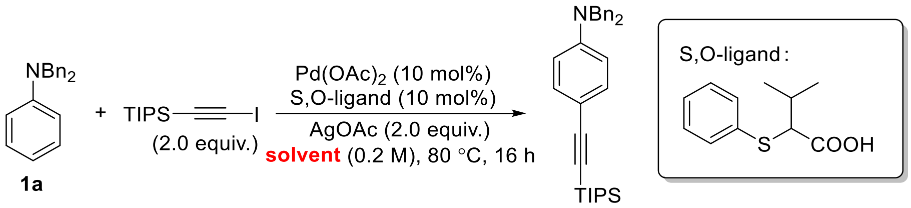

Entry	Solvent	Conversion	^1HNMR yield of alkynylated product
1	t-Amyl-OH	12%	9%
2	C_6F_6	34%	24%
3	HFIP	messy	
4	DMF	21%	10%
5	CH_2Cl_2	58%	39%
6	CH_3CN	5%	traces

7	EtOAc	35%	26%
8	1,4-dioxane	33%	14%
**9**	**$CHCl_3$**	**52%**	**41%**
10	DCE	56%	37%

[a]Reaction conditions: **1a** (27.3 mg, 0.1 mmol, 1.0 equiv.), alkyne source (61.6 mg, 0.2 mmol, 2.0 equiv.), $Pd(OAc)_2$ (2.2 mg, 0.01 mmol, 10 mol%), S,O-ligand (2.1 mg, 0.01 mmol, 10 mol%), AgOAc (33.4 g, 0.2 mmol, 2.0 equiv.), solvent (0.5 mL), 80 °C, 16 h. [b]The yields were determined by ^1H NMR analysis of crude reaction samples using CH_2Br_2 as internal standard.

Table S3. Screening of silver salts. [a,b]

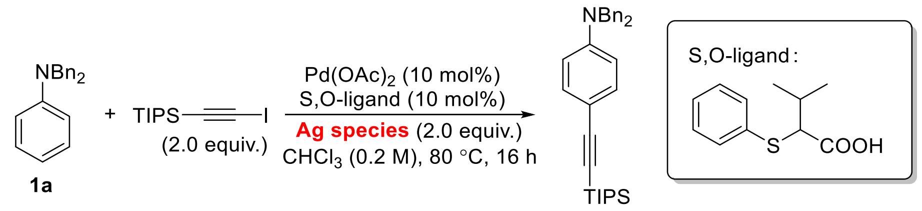

Entry	Ag species	Conversion	^{1}HNMR yield of alkynylated product
**1**	**AgOAc**	**52%**	**41%**
2	Ag_2O	61%	20%
3	Ag_2CO_3	21%	11%
4	AgTFA	messy	
5	$AgNO_3$	messy	
6	AgOTf	messy	

[a]Reaction conditions: **1a** (27.3 mg, 0.1 mmol, 1.0 equiv.), alkyne source (61.6 mg, 0.2 mmol, 2.0 equiv.), $Pd(OAc)_2$ (2.2 mg, 0.01 mmol, 10 mol%), S,O-ligand (2.1 mg, 0.01 mmol, 10 mol%), Ag species (0.2 mmol, 2.0 equiv.), $CHCl_3$ (0.5 mL), 80 °C, 16 h. [b]The yields were determined by ^1H NMR analysis of crude reaction samples using CH_2Br_2 as internal standard.

Table S4. Screening of temperatures. [a,b]

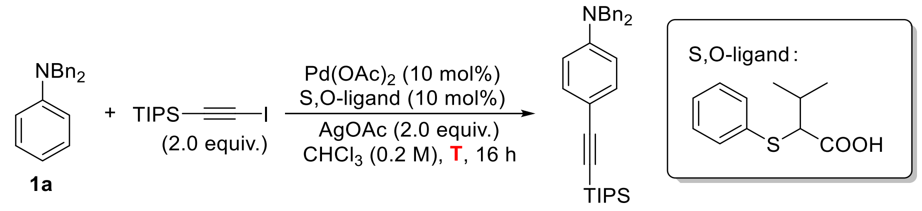

Entry	T/°C	Conversion	^{1}HNMR yield of alkynylated product
1	70	49%	39%
**2**	**80**	**52%**	**41%**
3	90	44%	36%
4	100	52%	34%

[a]Reaction conditions: **1a** (27.3 mg, 0.1 mmol, 1.0 equiv.), alkyne source (61.6 mg, 0.2 mmol, 2.0 equiv.), $Pd(OAc)_2$ (2.2 mg, 0.01 mmol, 10 mol%), S,O-ligand (2.1 mg, 0.01 mmol, 10 mol%), AgOAc (33.4 g, 0.2 mmol, 2.0 equiv.), $CHCl_3$ (0.5 mL), 80 °C, 16 h. [b]The yields were determined by ^1H NMR analysis of crude reaction samples using CH_2Br_2 as internal standard.

Table S5. Screening of palladium salts. [a,b]

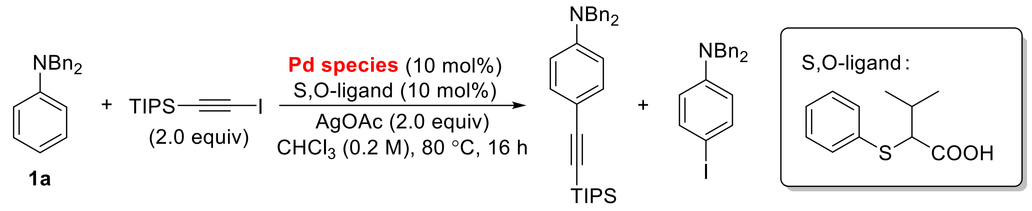

Entry	Pd species	^{1}HNMR yield of iodinated product	^{1}HNMR yield of alkynylated product
1	$Pd(OPiv)_2$	5%	26%
2	$Pd(TFA)_2$	39%	7%
3	$PdCl_2$	90%	4%
4	$Pd(CH_3CN)_2Cl_2$	4%	30%
5	$Pd(acac)_2$	traces	23%
**6**	**$Pd(OAc)_2$**	**6%**	**41%**

[a]Reaction conditions: **1a** (27.3 mg, 0.1 mmol, 1.0 equiv.), alkyne source (61.6 mg, 0.2 mmol, 2.0 equiv.), Pd species (0.01 mmol, 10 mol%), S,O-ligand (2.1 mg, 0.01 mmol, 10 mol%), AgOAc (33.4 g, 0.2 mmol, 2.0 equiv.), $CHCl_3$ (0.5 mL), 80 °C, 16 h. [b]The yields were determined by ^{1}H NMR analysis of crude reaction samples using CH_2Br_2 as internal standard.

Table S6. Screening of additives. [a,b]

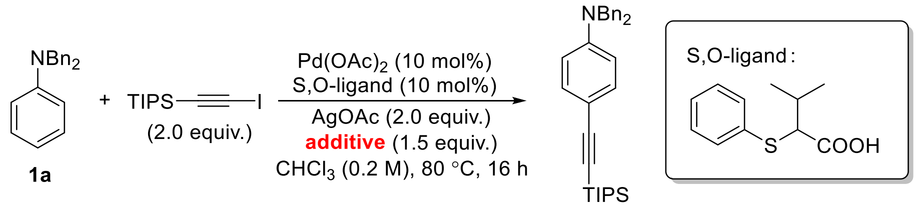

Entry	Additive	^{1}HNMR yield of alkynylated product
1	NaOAc	34%
2	Na_2CO_3	33%
3	K_2CO_3	14%
4	$NaHCO_3$	30%
5	Cs_2CO_3	0%
6	K_2HPO_4	23%
7	$NaH_2PO_4 \cdot 2H_2O$	34%
8	Na_2HPO_4	33%
**9**	**-**	**41%**

[a]Reaction conditions: **1a** (27.3 mg, 0.1 mmol, 1.0 equiv.), alkyne source (61.6 mg, 0.2 mmol, 2.0 equiv.), $Pd(OAc)_2$ (2.2 mg, 0.01 mmol, 10 mol%), S,O-ligand (2.1 mg, 0.01 mmol, 10 mol%), AgOAc (33.4 g, 0.2 mmol, 2.0 equiv.), $CHCl_3$ (0.5 mL), 80 °C, 16 h. [b]The yields were determined by ^{1}H NMR analysis of crude reaction samples using CH_2Br_2 as internal standard.

Table S7. Screening of S,O-ligands. [a,b]

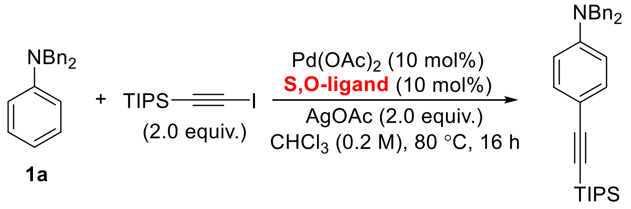

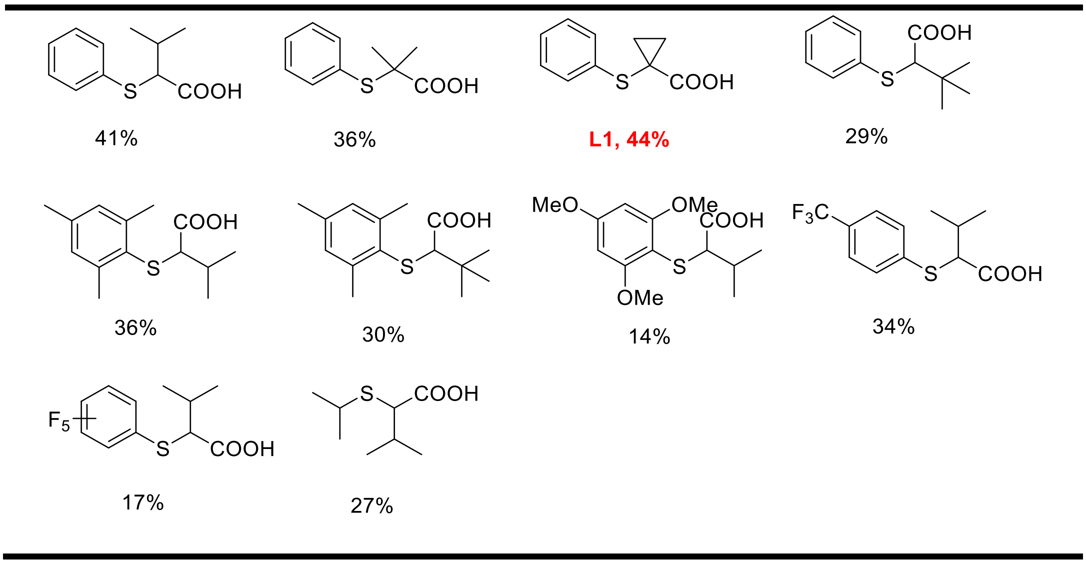

[a]Reaction conditions: **1a** (27.3 mg, 0.1 mmol, 1.0 equiv.), alkyne source (61.6 mg, 0.2 mmol, 2.0 equiv.), $Pd(OAc)_2$ (2.2 mg, 0.01 mmol, 10 mol%), **S,O-ligand** (0.01 mmol, 10 mol%), AgOAc (33.4 g, 0.2 mmol, 2.0 equiv.), $CHCl_3$ (0.5 mL), 80 °C, 16 h. [b]The yields were determined by ^1H NMR analysis of crude reaction samples using CH_2Br_2 as internal standard.

Table S8. Screening of alkyne sources. [a,b]

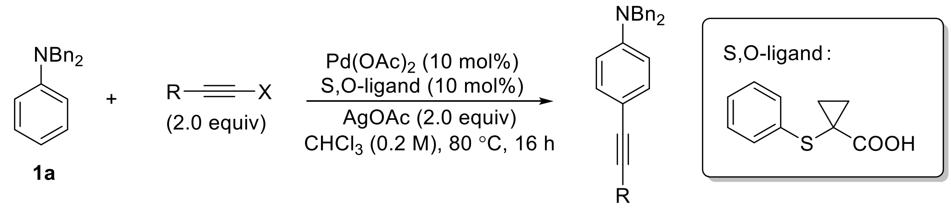

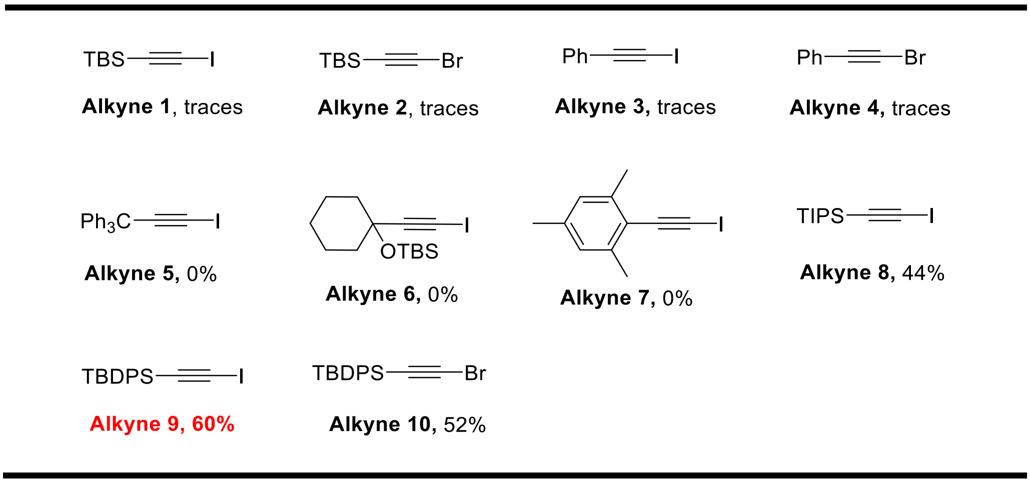

[a]Reaction conditions: **1a** (27.3 mg, 0.1 mmol, 1.0 equiv.), **alkyne source** (0.2 mmol, 2.0 equiv.), $Pd(OAc)_2$ (2.2 mg, 0.01 mmol, 10 mol%), S,O-ligand (2.1 mg, 0.01 mmol, 10 mol%), AgOAc (33.4 g, 0.2 mmol, 2.0 equiv.), $CHCl_3$ (0.5 mL), 80 °C, 16 h. [b]The yields were determined by ^1H NMR analysis of crude reaction samples using CH_2Br_2 as internal standard.

3. Synthesis of aniline substrates

Substrates **1a-1h** and **1o** were prepared following the general procedure reported in literature:[4]

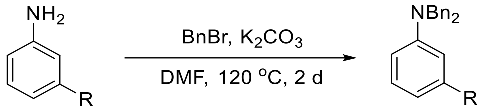

To a solution of *m*-substituted aniline (1.0 equiv.) in DMF (0.5 M) was added K_2CO_3 (5.0 equiv.) and $BnBr_2$ (4.0 equiv.) under nitrogen atmosphere at room temperature. The mixture was then stirred for 2 days at 120 °C. The reaction was then stopped by adding Et_2NH (2.5 equiv.) at 0 °C. The mixture was extracted with CH_2Cl_2 three times and the combined organic extracts were washed with brine, dried over anhydrous $MgSO_4$, filtered and concentrated under reduced pressure. The product was purified by flash column chromatography.

***N,N*-Dibenzyl-3-fluoroaniline (1b)**

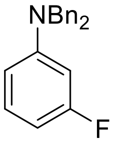

1b

N,N-Dibenzyl-3-fluoroaniline (**1b**) was prepared following the general procedure using 3-fluoroaniline (1.92 mL, 20 mmol, 1.0 equiv.), K_2CO_3 (13.82 g, 100 mmol, 5.0 equiv.), $BnBr_2$ (9.51 mL, 80 mmol, 4.0 equiv.) in DMF (40 mL). Et_2NH (5.19 mL, 50 mmol, 2.5 equiv.) was added to the mixture. Purification by column chromatography on silica gel using pentane:CH_2Cl_2 (10:1, v:v) as an eluent provided the titled compound as a white solid (4.66 g, 80% yield). **^{1}H NMR** (300 MHz, Chloroform-*d*) δ 7.43 – 7.31 (m, 7H), 7.29 – 7.26 (m, 3H), 7.17 – 7.09 (m, 1H), 6.54 (dd, *J* = 8.4, 2.4 Hz, 1H), 6.50 – 6.40 (m, 2H), 4.69 (s, 4H). **^{13}C NMR** (101 MHz, Chloroform-*d*) δ 164.2 (d, *J* = 243.4 Hz), 151.0 (d, *J* = 10.1 Hz), 138.0, 130.3 (d, *J* = 10.1 Hz), 128.8, 127.1, 126.6, 108.1 (d, *J* = 2.0 Hz), 103.3 (d, *J* = 22.2 Hz), 99.5 (d, *J* = 26.3 Hz), 54.3. **IR**: ν 3029, 1620, 1499, 1162, 754, 734, 696 cm^{-1}. **HRMS** (ESI) m/z: $[M+H]^+$ calculated for $C_{20}H_{18}FN^+$ 292.1502; found: 292.1513.

***N,N*-Dibenzyl-3-chloroaniline (1d)**

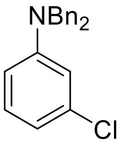

1d

N,N-Dibenzyl-3-chloroaniline (**1d**) was prepared following the general procedure using 3-chloroaniline (0.83 mL, 9 mmol, 1.0 equiv.), K_2CO_3 (6.22 g, 45 mmol, 5.0 equiv.), $BnBr_2$ (4.28 mL, 36 mmol, 4.0 equiv.) in DMF (20 mL). Et_2NH (2.33 mL, 22.5 mmol, 2.5 equiv.) was added to the mixture. Purification by column chromatography on silica gel using pentane/CH_2Cl_2 (10:1, v:v) as an eluent provided the titled compound as a white solid (2.30 g, 83% yield). ^{1}H NMR spectra data of the isolated material matched with the spectra data reported in the literature for this compound.[5] **^{1}H NMR** (400 MHz, Chloroform-*d*) δ 7.38 – 7.29 (m, 6H), 7.26 – 7.25 (m, 4H), 7.09 (t, *J* = 8.8 Hz, 1H), 7.77 (t, *J* = 0.8 Hz, 1H), 6.68 (dd, *J* = 25.6, 8.0 Hz, 2H), 4.66 (s, 4H).

***N,N*-Dibenzyl-3-bromoaniline (1e)**

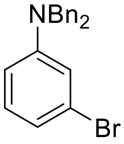

1e

N,N-Dibenzyl-3-bromoaniline (**1e**) was prepared following the general procedure using 3-bromoaniline (0.54 mL, 5 mmol, 1.0 equiv.), K_2CO_3 (3.46 g, 25 mmol, 5.0 equiv.), $BnBr_2$ (2.37 mL, 20 mmol, 4.0 equiv.) in DMF (10 mL). Et_2NH (1.29 mL, 12.5 mmol, 2.5 equiv.) was added to the mixture. Purification by column chromatography on silica gel using pentane/CH_2Cl_2 (10:1, v:v) as an eluent provided the titled compound as a white solid (1.31 g, 74% yield). ^{1}H NMR spectra data of the isolated material matched with the spectra data reported in the literature for this compound.[6] **^{1}H NMR** (400 MHz, Chloroform-*d*) δ 7.39 – 7.35 (m, 4H), 7.32 – 7.29 (m, 2H), 7.27 – 7.24 (m, 4H), 7.03 (t, *J* = 8.0 Hz, 1H), 6.93 (t, *J* = 2.0 Hz, 1H), 6.86 – 6.84 (m, 1H), 6.68 – 6.65 (m, 1H), 4.66 (s, 4H).

_N,N_-Dibenzyl-3-methylaniline (1f)

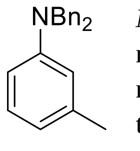

1f

N,N-Dibenzyl-3-methylaniline (**1f**) was prepared following the general procedure using 3-methylaniline (0.96 mL, 9 mmol, 1.0 equiv.), K_2CO_3 (6.22 g, 45 mmol, 5.0 equiv.), $BnBr_2$ (4.28 mL, 36 mmol, 4.0 equiv.) in DMF (20 mL). Et_2NH (2.33 mL, 22.5 mmol, 2.5 equiv.) was added to the mixture. Purification by column chromatography on silica gel using pentane/Et_2O (50:1, v:v) as an eluent provided the titled compound as a white solid (1.61 g, 62% yield). ^{1}H NMR spectra data of the isolated material matched with the spectra data reported in the literature for this compound.[5] **^{1}H NMR** (400 MHz, Chloroform-*d*) δ 7.38 – 7.29 (m, 7H), 7.27 – 7.24 (m, 3H), 7.09 (t, *J* = 10.4 Hz, 1H), 6.63 – 6.59 (m, 3H), 4.66 (s, 4H), 2.27 (s, 3H).

_N,N_-Dibenzyl-3-methoxyaniline (1g)

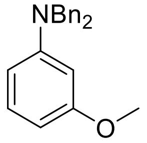

1g

N,N-Dibenzyl-3-methoxyaniline (**1g**) was prepared following the general procedure using m-anisidine (0.45 mL, 4 mmol, 1.0 equiv.), K_2CO_3 (2.76 g, 20 mmol, 5.0 equiv.), $BnBr_2$ (1.91 mL, 16 mmol, 4.0 equiv.) in DMF (10 mL). Et_2NH (2.33 mL, 22.5 mmol, 2.5 equiv.) was added to the mixture. Purification by column chromatography on silica gel using pentane/Et_2O (50:1, v:v) as an eluent provided the titled compound as a yellow oil (1.03 g, 85% yield). ^{1}H NMR spectra data of the isolated material matched with the spectra data reported in the literature for this compound.[7] **^{1}H NMR** (400 MHz, Chloroform-*d*) δ 7.40 – 7.32 (m, 6H), 7.27 – 7.25 (m, 4H), 7.10 (t, *J* = 8.0 Hz, 1H), 6.41 (t, *J* = 8.4 Hz, 1H), 6.34 – 6.31 (m, 2H), 4.66 (s, 4H), 3.72 (s, 3H).

_N,N_-Dibenzyl-3-phenoxyaniline (1h)

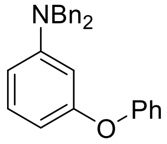

1h

N,N-Dibenzyl-3-phenoxyaniline (**1h**) was prepared following the general procedure using 3-phenoxyaniline (0.8 mL, 5 mmol, 1.0 equiv.), K_2CO_3 (3.46 g, 25 mmol, 5.0 equiv.), $BnBr_2$ (2.37 mL, 20 mmol, 4.0 equiv.) in DMF (10 mL). Et_2NH (2.33 mL, 22.5 mmol, 2.5 equiv.) was added to the mixture. Purification by column chromatography on silica gel using pentane/Et_2O (50:1, v:v) as an eluent provided the titled compound as a colorless liquid (1.58 g, 87% yield). **^{1}H NMR** (300 MHz, Chloroform-*d*) δ 7.43 – 7.30 (m, 8H), 7.28 – 7.25 (m, 4H), 7.14 (t, *J* = 8.1 Hz, 1H), 7.11 – 7.06 (m, 1H), 7.02 – 6.98 (m, 2H), 6.54 (dd, *J* = 8.1, 2.1 Hz, 1H), 6.49 (t, *J* = 2.4 Hz, 1H), 6.36 (dd, *J* = 8.1, 2.1 Hz, 1H), 4.67 (s, 4H). **^{13}C NMR** (75 MHz, Chloroform-*d*) δ 158.3, 157.1, 150.8, 138.3, 130.1, 129.6, 128.7, 127.0, 126.7, 122.9, 118.8, 107.7, 106.9, 103.4, 54.4. **IR**: ν 3026, 1487, 1215, 752, 732, 691 cm^{-1}. **HRMS** (ESI) m/z: $[M+H]^+$ calculated for $C_{26}H_{23}NO^+$ 366.1858; found: 366.1869.

3-Fluoro-_N,N_-diethylaniline (1o)

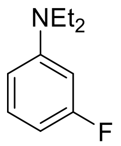

1o

3-Fluoro-*N,N*-diethylaniline (**1o**) was prepared following the general procedure using 3-ethylaniline (0.48 mL, 5 mmol, 1.0 equiv.), K_2CO_3 (3.45 g, 25 mmol, 5.0 equiv.), C_2H_5Br (1.50 mL, 20 mmol, 4.0 equiv.) in DMF (10 mL). Et_2NH (1.29 mL, 12.5 mmol, 2.5 equiv.) was added to the mixture. Purification by column chromatography on silica gel using pentane:CH_2Cl_2 (10:1, v:v) as an eluent provided the titled compound as a yellow liquid (0.65 g, 78% yield). **^{1}H NMR** (400 MHz, Chloroform-*d*) δ 7.03 (q, *J* = 7.6 Hz, 1H), 6.33 (dd, *J* = 8.4, 2.0 Hz, 1H), 6.28 – 6.21 (m, 2H), 3.25 (q, *J* = 7.2 Hz, 4H), 1.08 (t, *J* = 6.8 Hz, 6H). **^{13}C NMR** (75 MHz, Chloroform-*d*) δ 164.5 (d, *J* = 239.3 Hz), 149.5 (d, *J* = 10.5 Hz), 130.2 (d, *J* = 10.5 Hz), 107.2 (d, *J* = 2.3 Hz), 101.6 (d, *J* = 21.8 Hz), 98.4 (d, *J* = 25.5 Hz), 44.5, 12.5. **IR**: ν 2970, 1617, 1500, 1201, 819, 749, 682 cm^{-1}. **HRMS** (ESI) m/z: $[M+H]^+$ calculated for $C_{10}H_{14}FN^+$ 168.1189; found: 168.1195.

3-Fluoro-*N*-methyl-*N*-phenylaniline (1p) was prepared following the procedure reported in the literature:[8]

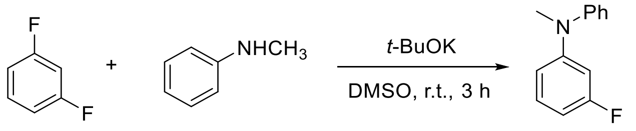

1p

A 25 mL vial was charged with a stir bar and *t*-BuOK (1.122 g, 10 mmol, 1.0 equiv.) in a glove box, capped and then moved out of the glove box. *N*-methylaniline (1.08 mL, 10 mmol, 1.0 equiv.), 1,3-difluorobenzene (0.98 mL, 10 mmol, 1.0 equiv.) and DMSO (10 mL) were injected into the vial and the mixture was stirred at room temperature for 3 hours. Then, the mixture was poured into water and extracted with CH_2Cl_2 three times. The combined organic extracts were washed with brine, dried over anhydrous $MgSO_4$, filtered and concentrated under reduced pressure. Purification by column chromatography on silica gel using pentane:CH_2Cl_2 (10:1, v:v) as an eluent provided the titled compound as an orange liquid (1.61 g, 80%).

^{1}H NMR (300 MHz, Chloroform-*d*) δ 7.40 – 7.34 (m, 2H), 7.19 – 7.10 (m, 4H), 6.70 – 6.66 (m, 1H), 6.64 – 6.53 (m, 2H), 3.33 (s, 3H). ^{1}H NMR spectra data of the isolated material matched with the spectra data reported in the literature for this compound.[8]

1-(3-Fluorophenyl)pyrrolidine (1q) was prepared following the procedure reported in the literature:[9]

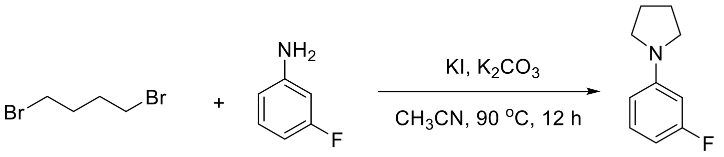

1q

To a solution of 1,4-dibromobutane (1.18 mL, 10 mmol, 1.0 equiv.) in CH_3CN (20 mL) was added KI (3.65 g, 22 mmol, 2.2 equiv.), K_2CO_3 (3.04 g, 22 mmol, 2.2 equiv.) and 3-fluoroaniline (1.25 mL, 13 mmol, 1.3 equiv.) at room temperature. The mixture was stirred at 90 °C for 12 hours. The mixture was then cooled to room temperature, filtered and concentrated under reduced pressure. Purification by column chromatography on silica gel using PE:EtOAc (20:1, v:v) as an eluent provided the titled compound as a yellow liquid (1.40 g, 85%).

^{1}H NMR (300 MHz, Chloroform-*d*) δ 7.20 – 7.12 (m, 1H), 6.40 – 6.32 (m, 2H), 6.29 (dt, *J* = 12.6, 2.4 Hz, 1H), 3.31 – 3.27 (m, 4H), 2.06 – 2.01 (m, 4H). ^{1}H NMR spectra data of the isolated material matched with the spectra data reported in the literature for this compound.[9]

4-(3-Fluorophenyl)morpholine (1r) was prepared following the procedure reported in the literature:[10]

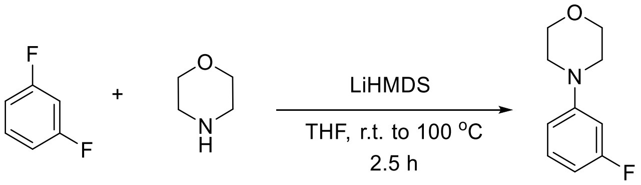

1r

To a vial morpholine (0.43 mL, 5 mmol, 1.0 equiv.) and lithium bis(trimethylsilyl)amide (1.0 M in THF, 7.5 mL, 1.5 equiv.) were added. The vial was sealed and stirred at 100 °C for 10 minutes. 1,3-Difluorobenzene was them added to the mixture (0.74 mL, 7.5 mmol, 1.5 equiv.) at room temperature. The reaction was stirred at 100 °C for 2.5 hours. The reaction was then quenched by adding solid $NaHCO_3$ and loaded directly onto a silica

gel column. Purification by column chromatography on silica gel using pentane:Et_2O (10:1, v:v) as an eluent provided the titled compound as a colorless liquid (0.46 g, 51%).

^{1}H NMR (400 MHz, Chloroform-*d*) δ 7.15 – 7.10 (m, 1H), 6.60 – 6.57 (m, 1H), 6.52 – 6.45 (m, 2H), 3.78 – 3.76 (m, 4H), 3.09 – 3.06 (m, 4H). ^{1}H NMR spectra data of the isolated material matched with the spectra data reported in the literature for this compound.[10]

4. Synthesis of alkyne sources

***tert*-Butyl((1-(iodoethynyl)cyclohexyl)oxy)dimethylsilane (Alkyne 6)** was prepared using the following synthetic sequence.

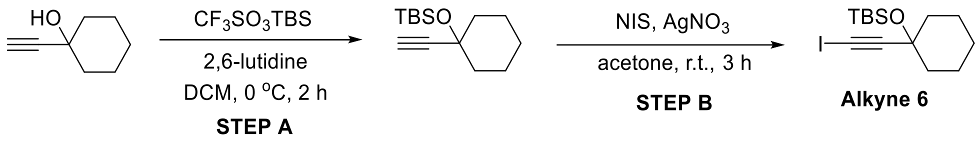

STEP A:[11] To a solution of 1-ethynyl-1-cyclohexanol (0.50 g, 4.0 mmol, 1.1 equiv.) in CH_2Cl_2 (10 mL) was added TBSOTf (0.83 mL, 3.6 mmol) at 0 °C. The mixture was stirred at 0 °C for 2 hours before water (10 mL) was added. After reaching room temperature, the organic layer was separated and the aqueous layer was extracted three times with CH_2Cl_2. The combined organic layers were washed with brine, dried over anhydrous $MgSO_4$, filtered and concentrated under reduced pressure. Purification by column chromatography on silica gel using hexane as an eluent provided the product as a colorless oil (0.64 g, 75%). **^{1}H NMR** (400 MHz, Chloroform-*d*) δ 2.47 (s, 1H), 1.85 – 1.79 (m, 2H), 1.72 – 1.61 (m, 4H), 1.58 – 1.51 (m, 2H), 1.48 – 1.40 (m, 1H), 1.38 – 1.29 (m, 1H), 0.91 (s, 9H), 0.20 (s, 6H). ^{1}H NMR spectra data of the isolated material matched with the spectra data reported in the literature for this compound.[11]

STEP B: To a solution of alkyne (0.71 g, 3 mmol, 1.0 equiv.) obtained from STEP A in acetone (10 mL) was added *N*-iodosuccinimide (0.74 g, 3.3 mmol, 1.1 equiv.) and $AgNO_3$ (51 mg, 0.3 mmol, 10 mol%). The reaction mixture was stirred under nitrogen at room temperature for 3 hours. The excess acetone was removed and the reaction was then quenched with saturated NH_4Cl solution. The aqueous layer was extracted three times with CH_2Cl_2. The combined organic layers were washed with brine, dried over anhydrous $MgSO_4$, filtered and concentrated under reduced pressure. Purification by column chromatography on silica gel using hexane as an eluent provided the titled compound as a colorless oil (0.86 g, 79%).

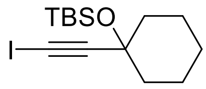

Alkyne 6

^{1}H NMR (400 MHz, Chloroform-*d*) δ 1.66 – 1.60 (m, 2H), 1.53 – 1.42 (m, 4H), 1.38 – 1.29 (m, 2H), 1.28 – 1.11 (m, 2H), 0.73 (s, 9H), 0.00 (s, 6H). **^{13}C NMR** (101 MHz, Chloroform-*d*) δ 98.8, 70.5, 40.6, 25.4, 25.3, 24.8, 22.2, 17.7, 0.0, -3.5. **IR**: ν 2931, 2855, 1462, 1252, 1154, 834, 774, 698 cm^{-1}. **HRMS** (FD) m/z: $[M]^+$ calculated for $C_{14}H_{25}IOSi^+$ 364.0719; found: 364.0730.

2-(Iodoethynyl)-1,3,5-trimethylbenzene (Alkyne 7) was prepared using the following synthetic sequence.

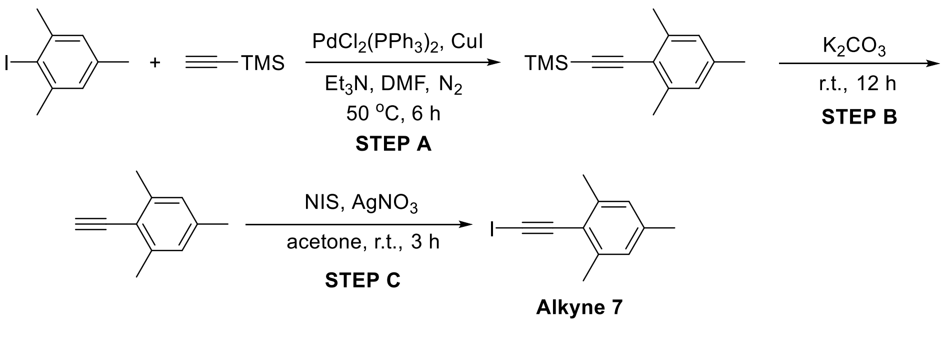

STEP A:[12] To a suitable vial was added 2-iodo-1,3,5-trimethylbenzene (1.23 g, 5 mmol, 1.0 equiv.), trimethylsilylacetylene (0.78 mL, 5.5 mmol, 1.1 equiv.), $Pd(PPh_3)_2Cl_2$ (0.18 g, 0.25 mmol, 5 mol%), CuI (0.95 g, 0.5 mmol, 10 mol%), triethylamine (1.82 g, 2.5 mL, 18 mmol, 3.6 equiv.) and DMF (10 mL). The mixture was degassed and stirred at 50 °C for 6 hours under nitrogen atmosphere. After the mixture was cooled to room temperature, water was added. The aqueous layer was extracted three times with Et_2O. The combined organic layers were washed with brine, dried over anhydrous $MgSO_4$, filtered and concentrated under reduced pressure. Purification by column chromatography on silica gel using pentane provided the product as a yellow oil (0.43 g, 40%). **^{1}H NMR** (400 MHz, Chloroform-*d*) δ 6.70 (s, 2H), 2.25 (s, 6H), 2.12 (s, 3H), 0.12 (s, 9H). ^{1}H NMR spectra data of the isolated material matched with the spectra data reported in the literature for this compound.[12]

STEP B:[12] To a solution of (mesitylethynyl)trimethylsilane (0.43, 2.0 mmol, 1.0 equiv.) in THF (7 mL) and MeOH (7 mL) was added K_2CO_3 (0.30 g, 2.2 mmol, 1.1 equiv.) at room temperature. After the mixture was stirred for 12 hours, it was concentrated under reduced pressure, washed with water, extracted three times with Et_2O, dried over anhydrous $MgSO_4$, filtered and concentrated under reduced pressure. Purification by column chromatography on silica gel using pentane provided the product as a yellow oil (0.16 g, 57%). **^{1}H NMR** (400 MHz, Chloroform-*d*) δ 6.90 (s, 2H), 3.48 (s, 1H), 2.44 (s, 6H), 2.31 (s, 3H). ^{1}H NMR spectra data of the isolated material matched with the spectra data reported in the literature for this compound.[12]

STEP C: To a solution of 2-ethynyl-1,3,5-trimethylbenzene (0.16 g, 1.1 mmol, 1.0 equiv.) was added *N*-iodosuccinimide (0.28 g, 1.2 mmol, 1.1 equiv.) and $AgNO_3$ (18.7 mg, 0.11 mmol, 10 mol%) in acetone (5 mL). The resulting mixture was stirred under nitrogen at room temperature for 3 hours. The excess acetone was removed and the reaction was then quenched with saturated NH_4Cl solution. The aqueous layer was extracted three times with CH_2Cl_2. The combined organic layers were washed with brine, dried over anhydrous $MgSO_4$, filtered and concentrated under reduced pressure. Purification by column chromatography on silica gel using hexane as an eluent provided the titled compound (92 mg, 31%).

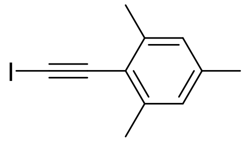

Alkyne 7

^{1}H NMR (400 MHz, Chloroform-*d*) δ 6.77 (s, 2H), 2.32 (s, 6H), 2.20 (s, 3H). ^{1}H NMR spectra data of the isolated material matched with the spectra data reported in the literature for this compound.[13]

***tert*-Butyl(iodoethynyl)diphenylsilane (Alkyne 9)** was prepared using the following synthetic sequence.

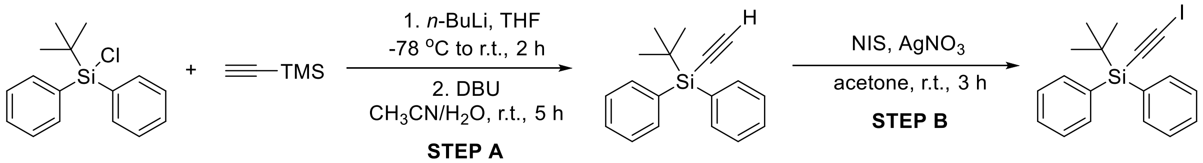

STEP A:[14] To a flame-dried round-bottom flask was charged with *n*-BuLi (2.5 M in hexane, 15.6 mL, 39 mmol, 1.3 equiv.) anhydrous THF (50 mL) under nitrogen atmosphere. The mixture was cooled to -78 °C in a dry ice/acetone bath. Trimethylsilylacetylene (5.6 mL, 39 mmol, 1.3 equiv.) was added dropwise and the mixture was stirred for 15 minutes at - 78 °C. Then *tert*-butyl(chloro)diphenylsilane (7.8 mL, 30 mmol) was added dropwise and the mixture was allowed to warm up to room temperature and stirred for 2 hours. The reaction was quenched with saturated NH_4Cl solution and the mixture was extracted three times with Et_2O. The combined organic layers were washed with brine and dried over anhydrous $MgSO_4$, filtered and concentrated under reduced pressure. The crude product was subjected to the next step without further purification. The crude product was dissolved in CH_3CN (60 mL) and water (3 mL). DBU (4.5 mL, 30 mmol, 1.0 equiv.) was added into the reaction. The mixture was stirred for 5 hours at room temperature and then concentrated under reduced pressure. Purification by column chromatography on silica gel using pentane provided the product as a colorless solid (3.9 g, 49% for two steps). **^{1}H NMR** (400 MHz,

Chloroform-*d*) δ 7.84 – 7.82 (m, 4H), 7.46 – 7.38 (m, 6H), 2.74 (s, 1H), 1.13 (s, 9H). ^{1}H NMR spectra data of the isolated material matched with the spectra data reported in the literature for this compound.[14]

STEP B: To a solution of *tert*-butyl(ethynyl)diphenylsilane (3.9 g, 14.7 mmol, 1.0 equiv.) was added *N*-iodosuccinimide (3.6 g, 16.2 mmol, 1.1 equiv.) and $AgNO_3$ (0.25 g, 1.5 mmol, 10 mol%) in acetone (70 mL). The resulting mixture was stirred under nitrogen at room temperature for 3 hours. The excess acetone was removed and the reaction was then quenched with saturated NH_4Cl solution. The aqueous layer was extracted three times with CH_2Cl_2. The combined organic layers were washed with brine, dried over anhydrous $MgSO_4$, filtered and concentrated under reduced pressure. Purification by column chromatography on silica gel using hexane as an eluent provided the titled compound as a white solid (4.82 g, 84%).

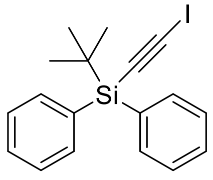

Alkyne 9

^{1}H NMR (300 MHz, Chloroform-*d*) δ 7.82– 7.78 (m, 4H), 7.45 – 7.37 (m, 6H), 1.12 (s, 9H). **^{13}C NMR** (75 MHz, Chloroform-*d*) δ 135.6, 132.8, 129.7, 127.8, 99.6, 27.0, 24.3, 18.9. **IR**: ν 3069, 2928, 2856, 2097, 1427, 1109, 763, 738, 698 cm^{-1}. **HRMS** (FI) m/z: $[M]^+$ calculated for $C_{18}H_{19}ISi^+$ 390.0301; found: 390.0314.

5. C–H alkynylation of aniline derivatives via Pd/S,O-ligand catalysis

General Procedure: In a pressure tube containing a suitable stirring bar was added the corresponding aniline (0.2 mmol, 1.0 equiv.), alkyne (0.4 mmol, 2.0 equiv.), $Pd(OAc)_2$ (4.5 mg, 0.02 mmol, 10 mol%), AgOAc (66.8 mg, 0.4 mmol, 2.0 equiv.), S,O-ligand (1-(phenylthio)cyclopropane-1-carboxylic acid, **L1**) (200 μL, 0.1 M stock solution in $CHCl_3$, 0.02 mmol, 10 mol%) and 0.8 mL of $CHCl_3$. The tube was put in a pre-heated oil bath at 80 ℃ and was stirred for 16 h. After cooling to room temperature, the resulting mixture was diluted with CH_2Cl_2, filtered through a plug of Celite and concentrated under reduced pressure. The ^{1}H NMR yield was determined by adding CH_2Br_2 (10 μL, 0.143 mmol, 0.715 equiv) as an internal standard. The crude mixture was purified by column chromatography to obtain the desired product.

***N,N*-Dibenzyl-4-((*tert*-butyldiphenylsilyl)ethynyl)aniline (2a)**

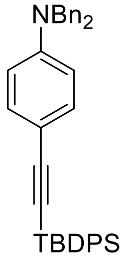

2a

General procedure was followed using *N,N*-dibenzylaniline (**1a**) (54.6 mg, 0.2 mmol, 1.0 equiv.) and *tert*-butyl(iodoethynyl)diphenylsilane (156.2 mg, 0.4 mmol, 2.0 equiv.) in $CHCl_3$ (1.0 mL, 0.2 M) at 80 °C, providing **2a** in 58% ^{1}H NMR yield. Purification by column chromatography on silica gel using pentane: Et_2O (50:1 v/v) as an eluent provided the titled compound as a yellow solid (62.4 mg, 58% yield). **^{1}H NMR** (300 MHz, Chloroform-*d*) δ 7.78 – 7.60 (m, 4H), 7.34 – 7.26 (m, 2H), 7.25 – 7.23 (m, 2H), 7.15 – 7.13 (m, 5H), 6.60 (d, *J* = 8.8 Hz, 2H), 4.60 (s, 4H), 1.03 (s, 9H). **^{13}C NMR** (101 MHz, Chloroform-*d*) δ 149.3, 137.9, 135.7, 134.0, 133.6, 129.3, 128.8, 127.7, 127.2, 126.5, 112.0, 110.6, 110.5, 86.6, 54.2, 27.2, 18.8. **IR**: ν 3068, 2928, 2855, 2148, 1605, 1516, 1107, 818, 740, 698 cm^{-1}. **HRMS** (ESI) m/z: $[M+H]^+$ calculated for $C_{38}H_{37}NSi^+$ 536.2774; found: 536.2769; M.p. 106 – 108 °C.

A parallel reaction without ligand was also performed, and 3% of product was observed by ^{1}H NMR analysis with CH_2Br_2 as internal standard.

_N,N_-Dibenzyl-4-((_tert_-butyldiphenylsilyl)ethynyl)-3-fluoroaniline (2b)

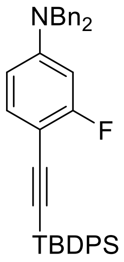

2b

General procedure was followed using *N,N*-dibenzyl-3-fluoroaniline (**1b**) (58.2 mg, 0.2 mmol, 1.0 equiv.) and *tert*-butyl(iodoethynyl)diphenylsilane (156.2 mg, 0.4 mmol, 2.0 equiv.) in $CHCl_3$ (1.0 mL, 0.2 M) at 80 °C, providing **2b** in 74% ^{1}H NMR yield. Purification by column chromatography on silica gel using pentane: CH_2Cl_2 (5:1 v/v) as an eluent provided the titled compound as a brown solid (76.9 mg, 69% yield). **^{1}H NMR** (300 MHz, Chloroform-*d*) δ 7.94 – 7.82 (m, 4H), 7.48 – 7.16 (m, 17H), 6.54 – 6.39 (m, 2H), 4.68 (s, 4H), 1.13 (s, 9H). **^{13}C NMR** (75 MHz, Chloroform-*d*) δ 163.8 (d, *J* = 249.4 Hz), 150.1 (d, *J* = 10.7 Hz), 136.2, 134.6, 133.4 (d, *J* = 3.7 Hz), 132.6, 128.3, 127.8, 126.6, 126.3, 125.4, 106.9 (d, *J* = 2.2 Hz), 102.4, 98.2 (d, *J* = 25.8 Hz), 98.0 (d, *J* = 16.4 Hz), 91.1, 91.1, 53.2, 26.1, 17.7. **IR**: ν 3070, 2929, 2855, 2154, 1623, 1517, 1109, 819, 740, 699 cm^{-1}. **HRMS** (ESI) m/z: $[M+H]^+$ calculated for $C_{38}H_{36}FNSi^+$ 554.2679; found: 554.2691; M.p. 122 – 124 °C.

A parallel reaction without ligand was also performed, and no product was observed by ^{1}H NMR analysis with CH_2Br_2 as internal standard.

<u>Large-scale synthesis:</u>

General procedure was followed using *N,N*-dibenzyl-3-fluoroaniline (**1b**) (0.87 g, 3 mmol, 1.0 equiv.), *tert*-butyl(iodoethynyl)diphenylsilane (2.34 g, 6 mmol, 2.0 equiv.), $Pd(OAc)_2$ (67.5 mg, 0.3 mmol, 10 mol%), AgOAc (1.00 g, 6.0 mmol, 2.0 equiv.) in $CHCl_3$ (15 mL, 0.2 M) at 80 °C. Purification by column chromatography on silica gel using pentane: CH_2Cl_2 (5:1 v/v) as an eluent provided the titled compound as a brown solid (1.26 g, 76% yield).

_N,N_-Dibenzyl-3-fluoro-4-((triisopropylsilyl)ethynyl)aniline (2c)

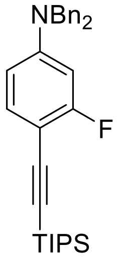

2c

General procedure was followed using *N,N*-dibenzyl-3-fluoroaniline (**1b**) (58.2 mg, 0.2 mmol, 1.0 equiv.) and 1-iodo-2-(triisopropylsilyl)acetylene (123.2 mg, 0.4 mmol, 2.0 equiv.) in $CHCl_3$ (1.0 mL, 0.2 M) at 80 °C. Purification by column chromatography on silica gel using pentane: CH_2Cl_2 (5:1 v/v) as an eluent provided the titled compound as a yellow oil (58.7 mg, 62% yield). **^{1}H NMR** (400 MHz, Chloroform-*d*) δ 7.38 – 7.31 (m, 4H), 7.29 – 7.19 (m, 7H), 6.44 – 6.38 (m, 2H), 4.65 (s, 4H), 1.12 – 1.11 (m, 21H). **^{13}C NMR** (75 MHz, Chloroform-*d*) δ 164.5 (d, *J* = 247.5 Hz), 150.7 (d, *J* = 10.5 Hz), 137.3, 134.4 (d, *J* = 3.8 Hz), 128.8, 127.3, 126.5, 107.9 (d, *J* = 2.2 Hz), 101.0, 99.6 (d, *J* = 16.5 Hz), 99.3 (d, *J* = 26.2 Hz), 93.2 (d, *J* = 2.2 Hz), 54.2, 18.7, 11.4. **IR**: ν 2942, 2864, 2150, 1707, 1623, 1516, 1360, 1227, 811, 734, 695 cm^{-1}. **HRMS** (FD) m/z: $[M]^+$ calculated for $C_{31}H_{38}FNSi^+$ 471.2758; found: 471.2780.

A parallel reaction without ligand was also performed, and no product was observed by ^{1}H NMR analysis with CH_2Br_2 as internal standard.

_N,N_-Dibenzyl-4-((_tert_-butyldiphenylsilyl)ethynyl)-3-chloroaniline (2d)

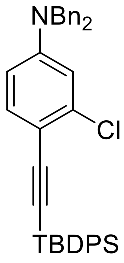

2d

General procedure was followed using *N,N*-dibenzyl-3-chloroaniline (**1d**) (61.6 mg, 0.2 mmol, 1.0 equiv.) and *tert*-butyl(iodoethynyl)diphenylsilane (156.2 mg, 0.4 mmol, 2.0 equiv.) in $CHCl_3$ (1.0 mL, 0.2 M) at 80 °C, providing **2d** in 63% ^{1}H NMR yield. Purification by column chromatography on silica gel using pentane: CH_2Cl_2 (5:1 v/v) as an eluent provided the titled compound as a brown solid (65.9 mg, 58% yield). **^{1}H NMR** (400 MHz, Chloroform-*d*) δ 7.92 – 7.90 (m, 4H), 7.42 – 7.34 (m, 11H), 7.32 – 7.28 (m, 2H), 7.23 – 7.21 (m, 4H), 6.82 (d, *J* = 2.6 Hz, 1H), 6.59 (dd, *J* = 8.8, 2.6 Hz, 1H), 4.68 (s, 4H), 1.15 (s, 9H). **^{13}C NMR** (75 MHz, Chloroform-*d*) δ 150.2, 138.1, 137.3, 135.9, 134.8, 133.8, 129.5, 129.0, 127.8, 127.5, 126.6, 112.6, 110.7, 110.7, 106.7, 92.0, 54.2, 27.3, 18.9. **IR**: ν 3067, 2929, 2856, 2154, 1600, 795, 735, 698 cm^{-1}. **HRMS** (FD) m/z: $[M]^+$ calculated for $C_{38}H_{36}ClNSi^+$ 569.2306; found: 569.2319; M.p. 126 – 129 °C.

A parallel reaction without ligand was also performed, and 4% product was observed by ^{1}H NMR analysis with CH_2Br_2 as internal standard.

N,N-Dibenzyl-3-bromo-4-((tert-butyldiphenylsilyl)ethynyl)aniline (2e)

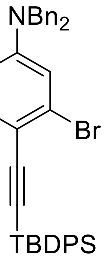

2e

General procedure was followed using *N,N*-dibenzyl-3-bromoaniline (**1e**) (70.5 mg, 0.2 mmol, 1.0 equiv.) and *tert*-butyl(iodoethynyl)diphenylsilane (156.2 mg, 0.4 mmol, 2.0 equiv.) in $CHCl_3$ (1.0 mL, 0.2 M) at 80 °C, providing **2e** in 67% ^{1}H NMR yield. Purification by column chromatography on silica gel using pentane: CH_2Cl_2 (10:1 v/v) as an eluent provided the titled compound as a brown solid (71.4 mg, 58% yield). **^{1}H NMR** (400 MHz, Methylene Chloride-d_2) δ 7.92 – 7.90 (m, 4H), 7.46 – 7.35 (m, 11H), 7.32 – 7.28 (m, 2H), 7.26 – 7.24 (m, 4H), 7.01 (d, *J* = 2.6 Hz, 1H), 6.67 (dd, *J* = 8.8, 2.6 Hz, 1H), 4.71 (s, 4H), 1.16 (s, 9H). **^{13}C NMR** (126 MHz, Methylene Chloride-d_2) δ 150.7, 137.9, 136.3, 135.2, 134.2, 130.0, 129.4, 128.3, 127.8, 127.7, 127.1, 116.1, 112.8, 111.9, 108.9, 91.6, 54.8, 27.5, 19.2. **IR**: ν 3068, 2928, 2856, 2153, 1598, 1503, 779, 738, 698 cm^{-1}. **HRMS** (ESI) m/z: $[M+H]^+$ calculated for $C_{38}H_{36}BrNSi^+$ 614.1879; found: 614.1878; M.p. 125 – 127 °C.

A parallel reaction without ligand was also performed, and traces of product was observed by ^{1}H NMR analysis with CH_2Br_2 as internal standard.

***N,N*-Dibenzyl-4-((*tert*-butyldiphenylsilyl)ethynyl)-3-methylaniline (2f)**

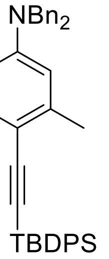

2f

General procedure was followed using *N,N*-dibenzyl-3-methylaniline (**1f**) (70.5 mg, 0.2 mmol, 1.0 equiv.) and *tert*-butyl(iodoethynyl)diphenylsilane (156.2 mg, 0.4 mmol, 2.0 equiv.) in $CHCl_3$ (1.0 mL, 0.2 M) at 80 °C, providing **2f** in 53% ^{1}H NMR yield. Purification by column chromatography on silica gel using pentane: CH_2Cl_2 (5:1 v/v) as an eluent provided the titled compound as a brown oil (55.8 mg, 51% yield). **^{1}H NMR** (400 MHz, Chloroform-*d*) δ 7.90 – 7.88 (m, 4H), 7.42 – 7.33 (m, 11H), 7.31 – 7.28 (m, 2H), 7.27 – 7.23 (m, 4H), 6.64 (d, *J* = 2.6 Hz, 1H), 6.56 (dd, *J* = 8.6, 2.7 Hz, 1H), 4.69 (s, 4H), 2.46 (s, 3H), 1.14 (s, 9H). **^{13}C NMR** (101 MHz, Chloroform-*d*) δ 149.5, 142.5, 138.1, 135.8, 134.2, 134.0, 129.4, 128.9, 127.7, 127.2, 126.7, 113.0, 109.8, 109.5, 90.4, 54.0, 27.3, 21.8, 18.9. **IR**: ν 3066, 2929, 2856, 2142, 1605, 1506, 1452, 1109, 819, 804, 699 cm^{-1}. **HRMS** (FD) m/z: $[M]^+$ calculated for $C_{39}H_{39}NSi^+$ 549.2852; found: 549.2840.

A parallel reaction without ligand was also performed, and no product was observed by ^{1}H NMR analysis with CH_2Br_2 as internal standard.

***N,N*-Dibenzyl-4-((*tert*-butyldiphenylsilyl)ethynyl)-3-methoxyaniline (2g)**

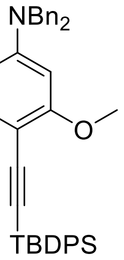

2g

General procedure was followed using *N,N*-dibenzyl-3-methoxyaniline (**1g**) (60.7 mg, 0.2 mmol, 1.0 equiv.) and *tert*-butyl(iodoethynyl)diphenylsilane (156.2 mg, 0.4 mmol, 2.0 equiv.) in $CHCl_3$ (1.0 mL, 0.2 M) at 80 °C, providing **2g** in 43% ^{1}H NMR yield. Purification by column chromatography on silica gel using pentane: Et_2O (40:1 v/v) as an eluent provided the titled compound as a brown oil (47.3 mg, 42% yield). **^{1}H NMR** (300 MHz, Chloroform-*d*) δ 7.94 – 7.90 (m, 4H), 7.39 – 7.24 (m, 17H), 6.34 (dd, *J* = 8.6, 2.4 Hz, 1H), 6.24 (d, *J* = 2.4 Hz, 1H), 4.71 (s, 4H), 3.72 (s, 3H), 1.14 (s, 9H). **^{13}C NMR** (75 MHz, Chloroform-*d*) δ 162.4, 151.0, 137.9, 135.8, 134.7, 134.2, 129.2, 128.8, 127.6, 127.2, 126.6, 107.0, 104.8, 100.6, 96.0, 90.7, 55.7, 54.5, 27.2, 18.8. **IR**: ν 3064, 2927, 2855, 2147, 1608, 1515, 821, 739, 699 cm^{-1}. **HRMS** (ESI) m/z: $[M+H]^+$ calculated for $C_{39}H_{39}NOSi^+$ 566.2879; found: 566.2871.

A parallel reaction without ligand was also performed, and 5% product was observed by ^{1}H NMR analysis with CH_2Br_2 as internal standard.

N,N-Dibenzyl-4-((*tert*-butyldiphenylsilyl)ethynyl)-3-phenoxyaniline (2h)

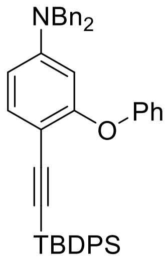

2h

General procedure was followed using *N,N*-dibenzyl-3-phenoxyaniline (**1h**) (73.1 mg, 0.2 mmol, 1.0 equiv.) and *tert*-butyl(iodoethynyl)diphenylsilane (156.2 mg, 0.4 mmol, 2.0 equiv.) in $CHCl_3$ (1.0 mL, 0.2 M) at 80 °C, providing **2h** in 79% ^{1}H NMR yield. Purification by column chromatography on silica gel using pentane: CH_2Cl_2 (8:1 v/v) as an eluent provided the titled compound as a brown solid (86.7 mg, 69% yield). **^{1}H NMR** (400 MHz, Dichloromethane-*d_2*) δ 7.81 – 7.78 (m, 4H), 7.49 (d, *J* = 8.8 Hz, 1H), 7.43 – 7.26 (m, 18H), 7.08 (t, *J* = 8.6, 7.6 Hz, 1H), 6.96 – 6.93 (m, 2H), 6.61 (dd, *J* = 8.8, 2.4 Hz, 1H), 6.43 (d, *J* = 2.4 Hz, 1H), 4.72 (s, 4H), 1.06 (s, 9H). **^{13}C NMR** (101 MHz, Chloroform-*d*) δ 160.5, 159.5, 152.8, 139.5, 137.6, 136.9, 135.8, 131.4, 131.2, 130.7, 129.5, 129.2, 128.5, 124.3, 119.2, 110.3, 107.8, 106.2, 105.8, 93.6, 56.5, 28.9, 20.5. **IR**: ν 3067, 2928, 2855, 2149, 1611, 1211, 821, 730, 694 cm^{-1}. **HRMS** (FD) m/z: $[M]^+$ calculated for $C_{44}H_{41}NOSi^+$ 627.2957; found: 627.2937; M.p. 139 – 141 °C.

A parallel reaction without ligand was also performed, and no product was observed by ^{1}H NMR analysis with CH_2Br_2 as internal standard.

Methyl 2-(benzylamino)-5-((*tert*-butyldiphenylsilyl)ethynyl)benzoate (2i)

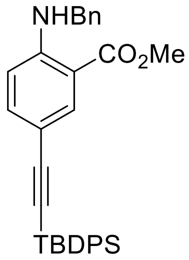

2i

General procedure was followed using methyl 2-(benzylamino)benzoate (**1i**) (48.3 mg, 0.2 mmol, 1.0 equiv.) and *tert*-butyl(iodoethynyl)diphenylsilane (156.2 mg, 0.4 mmol, 2.0 equiv.) in $CHCl_3$ (1.0 mL, 0.2 M) at 80 °C, providing **2i** in 43% ^{1}H NMR yield. Purification by column chromatography on silica gel using pentane: EtOAc (40:1 v/v) as an eluent provided the titled compound as a brown oil (43.0 mg, 43% yield). **^{1}H NMR** (400 MHz, Chloroform-*d*) δ 8.45 (t, *J* = 5.7 Hz, 1H), 8.19 (d, *J* = 2.1 Hz, 1H), 7.87 – 7.85 (m, 4H), 7.50 (dd, *J* = 8.8, 2.1 Hz, 1H), 7.41 – 7.38 (m, 10H), 7.31 – 7.27 (m, 1H), 6.62 (d, *J* = 8.8 Hz, 1H), 4.49 (d, *J* = 5.6 Hz, 2H), 3.90 (s, 3H), 1.14 (s, 9H). **^{13}C NMR** (75 MHz, Chloroform-*d*) δ 168.5, 150.9, 138.2, 138.1, 136.0, 135.7, 133.8, 129.4, 128.8, 127.7, 127.7, 127.4, 127.0, 111.8, 110.1, 109.4, 109.1, 86.6, 51.8, 46.9, 27.2, 18.8. **IR**: ν 3358, 3069, 2953, 2929, 2856, 2151, 1690, 1216, 819, 700 cm^{-1}. **HRMS** (FD) m/z: $[M]^+$ calculated for $C_{33}H_{33}NO_2Si^+$ 503.2281; found: 503.2293.

A parallel reaction without ligand was also performed, and no product was observed by ^{1}H NMR analysis with CH_2Br_2 as internal standard.

Methyl 2-(benzylamino)-5-((*tert*-butyldiphenylsilyl)ethynyl)-4-methylbenzoate (2j)

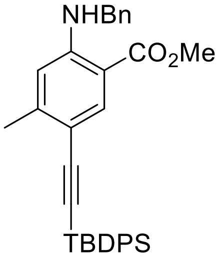

2j

General procedure was followed using methyl 2-(benzylamino)-4-methylbenzoate (**1j**) (51.1 mg, 0.2 mmol, 1.0 equiv.) and *tert*-butyl(iodoethynyl)diphenylsilane (156.2 mg, 0.4 mmol, 2.0 equiv.) in $CHCl_3$ (1.0 mL, 0.2 M) at 80 °C, providing **2j** in 50% ^{1}H NMR yield. Purification by column chromatography on silica gel using pentane: Et_2O (30:1 v/v) as an eluent provided the titled compound as a brown oil (51.7 mg, 50% yield). **^{1}H NMR** (400 MHz, Methylene Chloride-*d_2*) δ 8.38 (t, *J* = 5.7 Hz, 1H), 8.17 (s, 1H), 7.90 – 7.88 (m, 4H), 7.44 – 7.37 (m, 10H), 7.33 – 7.28 (m, 1H), 6.58 (s, 1H), 4.51 (d, *J* = 5.7 Hz, 2H), 3.86 (s, 3H), 2.48 (s, 3H), 1.16 (s, 10H). **^{13}C NMR** (126 MHz, Methylene Chloride-*d_2*) δ 168.8, 151.5, 148.3, 139.2, 136.8, 136.2, 134.4, 130.0, 129.3, 128.3, 127.9, 127.7, 112.8, 110.1, 109.0, 108.8, 90.6, 52.1, 47.3, 27.5, 22.2, 19.1. **IR**: ν 3357, 3069, 2951, 2929, 2856, 2144, 1687, 1223, 795, 738, 699 cm^{-1}. **HRMS** (ESI) m/z: $[M+H]^+$ calculated for $C_{34}H_{35}NO_2Si^+$ 518.2515; found: 518.2516.

A parallel reaction without ligand was also performed, and no product was observed by ^{1}H NMR analysis with CH_2Br_2 as internal standard.

Methyl 2-(benzylamino)-5-((*tert*-butyldiphenylsilyl)ethynyl)-4-methoxybenzoate (2k)

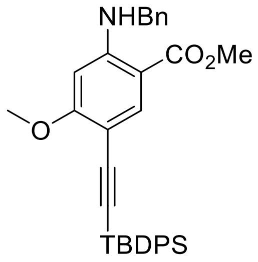

2k

General procedure was followed using methyl 2-(benzylamino)-4-methoxybenzoate (**1k**) (54.3 mg, 0.2 mmol, 1.0 equiv.) and *tert*-butyl(iodoethynyl)diphenylsilane (156.2 mg, 0.4 mmol, 2.0 equiv.) in $CHCl_3$ (1.0 mL, 0.2 M) at 80 °C, providing **2k** in 49% ^{1}H NMR yield. Purification by column chromatography on silica gel using pentane: Et_2O (15:1 v/v) as an eluent provided the titled compound as a brown oil (50.5 mg, 47% yield). **^{1}H NMR** (400 MHz, Chloroform-*d*) δ 8.64 – 8.61 (m, 1H), 8.16 (s, 1H), 7.93 – 7.91 (m, 3H), 7.41–7.38 (m, 10H), 7.33 – 7.30 (m, 1H), 6.03 (s, 1H), 5.32 (s, 1H), 4.52 (d, *J* = 5.2 Hz, 2H), 3.88 (s, 3H), 3.79 (s, 3H), 1.16 (s, 9H). **^{13}C NMR** (75 MHz, Chloroform-*d*) δ 168.3, 165.5, 153.1, 138.1, 137.9, 135.8, 134.0, 129.3, 128.8, 127.6, 127.4, 127.1, 105.7, 103.4, 100.2, 93.1, 90.6, 55.6, 51.5, 47.1, 27.2, 18.8. **IR**: ν 3482, 3069, 2953, 2930, 2856, 2365, 2148, 1683, 819, 798, 741, 700 cm^{-1}. **HRMS** (FD) m/z: $[M]^+$ calculated for $C_{34}H_{35}NO_3Si^+$ 533.2386; found: 533.2410.

A parallel reaction without ligand was also performed, and traces of product was observed by ^{1}H NMR analysis with CH_2Br_2 as internal standard.

Methyl 2-(benzylamino)-5-((*tert*-butyldiphenylsilyl)ethynyl)-4-fluorobenzoate (2l)

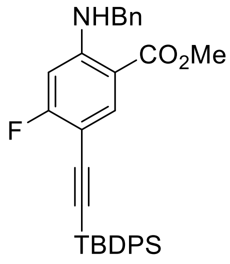

2l

Slightly modified procedure was followed using methyl 2-(benzylamino)-4-fluorobenzoate (**1l**) (51.9 mg, 0.2 mmol, 1.0 equiv.), *tert*-butyl(iodoethynyl)diphenylsilane (234.3 mg, 0.6 mmol, 3.0 equiv.) and AgOAc (100.2 mg, 0.6 mmol, 3.0 equiv.) in $CHCl_3$ (1.0 mL, 0.2 M) at 80 °C, providing **2l** in 40% ^{1}H NMR yield. Purification by column chromatography on silica gel using pentane: CH_2Cl_2 (4:1 v/v) as an eluent provided the titled compound as a yellow oil (38.8 mg, 37% yield). **^{1}H NMR** (400 MHz, Chloroform-*d*) δ 8.62 (t, *J* = 6.0 Hz, 1H), 8.23 (d, *J* = 8.4 Hz, 1H), 7.93 – 7.91 (m, 4H), 7.46 – 7.31 (m, 11H), 6.39 (d, *J* = 12.0 Hz, 1H), 4.48 (d, *J* = 5.6 Hz, 2H), 3.92 (s, 3H), 1.19 (s, 9H). **^{13}C NMR** (101 MHz, Chloroform-*d*) δ 167.8, 166.3 (d, *J* = 257.6 Hz), 153.0 (d, *J* = 12.1 Hz), 138.4 (d, *J* = 5.1 Hz), 137.5, 135.7, 133.5, 129.5, 128.9, 127.8, 127.6, 127.1, 107.1 (d, *J* = 2.02 Hz), 102.3, 98.8 (d, *J* = 18.2 Hz), 98.2 (d, *J* = 25.3 Hz), 92.3 (d, *J* = 2.2 Hz), 51.8, 47.1, 27.2, 18.8. **IR**: ν 3341, 3069, 2952, 2929, 2857, 2153, 1691, 1109, 699 cm^{-1}. **HRMS** (ESI) m/z: $[M+H]^+$ calculated for $C_{33}H_{32}FNO_2Si^+$ 522.2265; found: 522.2264.

A parallel reaction without ligand was also performed, and no product was observed by ^{1}H NMR analysis with CH_2Br_2 as internal standard.

***N*-benzyl-4-((*tert*-butyldiphenylsilyl)ethynyl)-2-chloro-5-methoxyaniline (2m)**

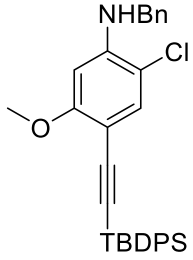

2m

Slightly modified procedure was followed using *N*-benzyl-2-chloro-5-methoxyaniline (**1m**) (99.1 mg, 0.4 mmol, 2.0 equiv.), *tert*-butyl(iodoethynyl)diphenylsilane (78.1 mg, 0.2 mmol, 1.0 equiv.) AgOAc (33.4 mg, 0.2 mmol, 1.0 equiv.) in $CHCl_3$ (1.0 mL, 0.2 M) at 100 °C, providing **2m** in 44% ^{1}H NMR yield. Purification by column chromatography on silica gel using pentane: Et_2O (50:1 v/v) as an eluent provided the titled compound as a yellow oil (41.2mg, 40% yield). **^{1}H NMR** (400 MHz, Chloroform-*d*) δ 7.93 – 7.91 (m, 4H), 7.46 (s, 1H), 7.43 – 7.37 (m, 18H), 7.35– 7.31 (m, 1H), 6.15 (s, 1H), 4.48 (d, *J* = 5.6 Hz, 2H), 3.79 (s, 3H), 1.16 (s, 9H). **^{13}C NMR** (101 MHz, Chloroform-*d*) δ 161.5, 145.3, 138.0, 135.8, 133.9, 133.4, 129.3, 128.9, 127.6, 127.6, 127.3, 109.9, 105.4, 101.3, 94.9, 91.7, 56.0, 47.8, 27.2, 18.8. **IR**: ν 3423, 3069, 2929, 2855, 2146, 1603, 1517, 847, 740, 699 cm^{-1}. **HRMS** (ESI) m/z: $[M+H]^+$ calculated for $C_{32}H_{32}ClNOSi^+$ 510.2020; found: 510.2005.

A parallel reaction without ligand was also performed, and no product was observed by ^{1}H NMR analysis with CH_2Br_2 as internal standard.

4-((*tert*-Butyldiphenylsilyl)ethynyl)-3-fluoro-*N,N*-dimethylaniline (2n)

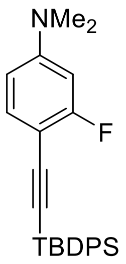

2n

Slightly modified procedure was followed using 3-fluoro-*N,N*-dimethylaniline (**1n**) (55.6 mg, 0.4 mmol, 2.0 equiv.) and *tert*-butyl(iodoethynyl)diphenylsilane (78.1 mg, 0.2 mmol, 1.0 equiv.) in $CHCl_3$ (1.0 mL, 0.2 M) at 80 °C, providing **2n** in 60% ^{1}H NMR yield. Purification by column chromatography on silica gel using pentane: Et_2O (40:1 v/v) as an eluent provided the titled compound as a brown solid (46.1 mg, 57% yield). **^{1}H NMR** (400 MHz, Chloroform-*d*) δ 7.82 – 7.79 (m, 4H), 7.33 – 7.28 (m, 7H), 6.312 (s, 1H), 6.311 (dd, *J* = 23.6, 2.8 Hz, 1H), 2.92 (s, 6H), 1.06 (s, 9H). **^{13}C NMR** (101 MHz, Chloroform-*d*) δ 164.8 (d, *J* = 242.4 Hz), 152.1 (d, *J* = 10.8 Hz), 135.7, 134.3 (d, *J* = 3.7 Hz), 133.8, 129.4, 127.7, 107.4 (d, *J* = 2.2 Hz), 103.8, 98.6 (d, *J* = 25.7 Hz), 98.2 (d, *J* = 16.6 Hz), 91.8 (d, *J* = 2.8 Hz), 40.2, 47.1, 27.2, 18.7. **IR**: ν 3070, 2929, 2856, 2150, 1623, 1109, 816, 743, 702 cm^{-1}. **HRMS** (ESI) m/z: $[M+H]^+$ calculated for $C_{26}H_{28}FNSi^+$ 402.2053; found: 402.2061; M.p. 101 – 103 °C.

A parallel reaction without ligand was also performed, and 12% product was observed by ^{1}H NMR analysis with CH_2Br_2 as internal standard.

4-((*tert*-Butyldiphenylsilyl)ethynyl)-*N,N*-diethyl-3-fluoroaniline (2o)

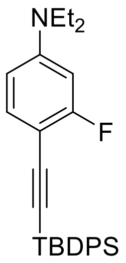

2o

General procedure was followed using *N,N*-diethyl-3-fluoroaniline (**1o**) (33.4 mg, 0.2 mmol, 1.0 equiv.) and *tert*-butyl(iodoethynyl)diphenylsilane (156.2 mg, 0.4 mmol, 2.0 equiv.) in $CHCl_3$ (1.0 mL, 0.2 M) at 80 °C, providing **2o** in 69% ^{1}H NMR yield. Purification by column chromatography on silica gel using pentane: CH_2Cl_2 (5:1 v/v) as an eluent provided the titled compound as an orange oil (58.2 mg, 68% yield). **^{1}H NMR** (400 MHz, Chloroform-*d*) δ 8.02 – 7.98 (m, 4H), 7.50 – 7.42 (m, 7H), 6.46 – 6.39 (m, 2H), 3.40 (q, *J* = 9.2 Hz, 4H), 1.26 – 1.21 (m, 15H). **^{13}C NMR** (75 MHz, Chloroform-*d*) δ 165.2 (d, *J* = 246.5 Hz), 149.8 (d, *J* = 10.8 Hz), 135.8, 134.5 (d, *J* = 3.7 Hz), 133.9, 129.4, 127.7, 107.1(d, *J* = 2.0 Hz), 104.2, 98.1 (d, *J* = 25.5 Hz), 97.2 (d, *J* = 16.6 Hz), 91.5 (d, *J* = 2.6 Hz), 44.7, 27.2, 18.8, 12.5. **IR**: ν 3070, 2961, 2930, 2856, 2153, 1622, 1519, 1108, 809, 742, 700 cm^{-1}. **HRMS** (FD) m/z: $[M]^+$ calculated for $C_{28}H_{32}FNSi^+$ 429.2288; found: 429.2284.

A parallel reaction without ligand was also performed, and traces of product was observed by ^{1}H NMR analysis with CH_2Br_2 as internal standard.

4-((*tert*-Butyldiphenylsilyl)ethynyl)-3-fluoro-*N*-methyl-*N*-phenylaniline (2p)

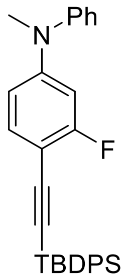

2p

Slightly modified procedure was followed using 3-fluoro-*N*-methyl-*N*-phenylaniline (**1p**) (80.4 mg, 0.4 mmol, 2.0 equiv.) and *tert*-butyl(iodoethynyl)diphenylsilane (78.1 mg, 0.2 mmol, 1.0 equiv.) in $CHCl_3$ (1.0 mL, 0.2 M) at 80 °C, providing **2p** in 46% ^{1}H NMR yield. Purification by column chromatography on silica gel using pentane: CH_2Cl_2 (5:1 v/v) as an eluent provided the titled compound as an orange oil (38.8 mg, 42% yield). **^{1}H NMR** (400 MHz, Chloroform-*d*) δ 7.92 – 7.90 (m, 4H), 7.45 – 7.35 (m, 9H), 7.26 – 7.22 (m, 3H), 6.560 – 6.555 (m, 1H), 6.54 – 6.52 (m, 1H), 3.36 (s, 3H), 1.17 (s, 9H). **^{13}C NMR** (101 MHz, Chloroform-*d*) δ 164.5 (d, *J* = 250.7 Hz), 151.1 (d, *J* = 10.6 Hz), 147.4, 135.7, 134.1 (d, *J* = 3.2 Hz), 133.6, 129.8, 129.4, 127.7 (d, *J* = 2.7 Hz), 125.7, 125.4, 110.6 (d, *J* = 2.5 Hz), 103.4, 101.8 (d, *J* = 25.5 Hz), 100.5 (d, *J* = 16.6 Hz), 92.6 (d, *J* = 2.9 Hz), 40.3, 27.1, 18.7. **IR**: ν 3069, 2929, 2855, 2152, 1621, 1105, 838, 820, 800, 697 cm^{-1}. **HRMS** (FD) m/z: $[M]^+$ calculated for $C_{31}H_{30}FNSi^+$ 463.2132; found: 463.2140.

A parallel reaction without ligand was also performed, and traces of product was observed by ^{1}H NMR analysis with CH_2Br_2 as internal standard.

1-(4-((*tert*-Butyldiphenylsilyl)ethynyl)-3-fluorophenyl)pyrrolidine (2q)

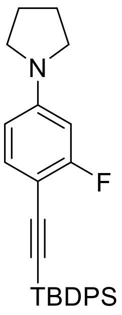

2q

General procedure was followed using 1-(3-fluorophenyl)pyrrolidine (**1q**) (33.0 mg, 0.2 mmol, 1.0 equiv.) and *tert*-butyl(iodoethynyl)diphenylsilane (156.2 mg, 0.4 mmol, 2.0 equiv.) in $CHCl_3$ (1.0 mL, 0.2 M) at 80 °C, providing alkynylated product **2q**. Purification by column chromatography on silica gel using pentane: CH_2Cl_2 (5:1 v/v) as an eluent provided the titled compound as a white solid (43.7 mg, 51% yield). **^{1}H NMR** (400 MHz, Chloroform-*d*) δ 7.82 – 7.80 (m, 4H), 7.32 – 7.28 (m, 7H), 6.170 (s, 1H), 6.166 (dd, *J* = 22.8, 2.4 Hz, 1H), 3.23 – 3.20 (m, 4H), 1.96 – 1.93 (m, 4H), 1.06 (s, 9H). **^{13}C NMR** (101 MHz, Chloroform-*d*) δ **164.9** (d, *J* = 250.5 Hz), 149.6 (d, *J* = 11.1 Hz), 135.7, 134.4 (d, *J* = 4.0 Hz), 133.9, 129.3, 127.7 (d, *J* = 6.1 Hz), 107.3 (d, *J* = 2.0 Hz), 104.2, 98.2 (d, *J* = 26.3 Hz), 97.4 (d, *J* = 16.2 Hz), 91.5 (d, *J* = 2.0 Hz), 47.7, 27.2, 25.4, 18.8. **IR**: ν 3070, 2957, 2929, 2855, 2149, 1623, 1109, 819, 810, 742, 701 cm^{-1}. **HRMS** (FD) m/z: $[M]^+$ calculated for $C_{28}H_{30}FNSi^+$ 427.2132; found: 427.2131; M.p. 80 – 82 °C.

A parallel reaction without ligand was also performed, and no product was observed by ^{1}H NMR analysis with CH_2Br_2 as internal standard.

4-(4-((*tert*-Butyldiphenylsilyl)ethynyl)-3-fluorophenyl)morpholine (2r)

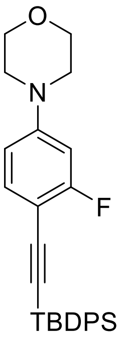

2r

Slightly modified procedure was followed using 4-(3-fluorophenyl)morpholine (**1r**) (72.5 mg, 0.4 mmol, 2.0 equiv.) and *tert*-butyl(iodoethynyl)diphenylsilane (78.1 mg, 0.2 mmol, 1.0 equiv.) in $CHCl_3$ (1.0 mL, 0.2 M) at 80 °C, providing **2r** in 47% ^{1}H NMR yield. Purification by column chromatography on silica gel using pentane: Et_2O (4:1 v/v) as an eluent provided the titled compound as a brown solid (39.0 mg, 44% yield). **^{1}H NMR** (400 MHz, Chloroform-*d*) δ 7.80 – 7.78 (m, 4H), 7.36 – 7.27 (m, 7H), 6.507 (s, 1H), 6.502 (dd, *J* = 22.0, 2.4 Hz, 1H), 3.77 – 3.74 (m, 4H), 3.13 – 3.11 (m, 4H), 1.06 (s, 9H). **^{13}C NMR** (101 MHz, Chloroform-*d*) δ 164.6 (d, *J* = 251.2 Hz), 152.9 (d, *J* = 9.9 Hz), 135.7, 134.4 (d, *J* = 3.2 Hz), 133.5, 129.5, 127.7, 110.0 (d, *J* = 2.5 Hz), 102.9, 101.63 (d, *J* = 16.4 Hz), 101.60 (d, *J* = 25.0 Hz), 93.0 (d, *J* = 2.9 Hz), 66.5, 48.0, 27.1, 18.8. **IR**: ν 3070, 3049, 2957, 2929, 2856, 2154, 1620, 1510, 1108, 803, 700 cm^{-1}. **HRMS** (FD) m/z: $[M]^+$ calculated for $C_{28}H_{30}FNOSi^+$ 443.2081; found: 443.2094; M.p. 112 – 114 °C.

A parallel reaction without ligand was also performed, and traces of product was observed by ^{1}H NMR analysis with CH_2Br_2 as internal standard.

1-(6-((*tert*-Butyldiphenylsilyl)ethynyl)-1,2,3,4-tetrahydroquinolin-8-yl)ethan-1-one (**2s**)

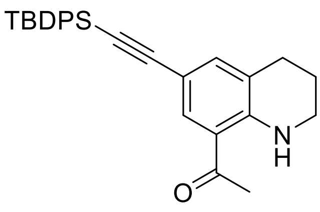

2s

General procedure was followed using 1-(1,2,3,4-tetrahydroquinolin-8-yl)ethan-1-one (**1s**) (35.0 mg, 0.2 mmol, 1.0 equiv.) and *tert*-butyl(iodoethynyl)diphenylsilane (156.2 mg, 0.4 mmol, 2.0 equiv.) in $CHCl_3$ (1.0 mL, 0.2 M) at 80 °C, providing **2s** in 52% ^{1}H NMR yield. Purification by column chromatography on silica gel using pentane: Et_2O (30:1 v/v) as an eluent provided the titled compound as a yellow oil (42.7 mg, 49% yield). **^{1}H NMR** (400 MHz, Chloroform-*d*) δ 9.30 (s, 1H), 7.92 – 7.89 (m, 4H), 7.85 (d, *J* = 1.6 Hz, 1H), 7.45 – 7.40 (m, 6H), 7.284– 7.277 (m, 1H), 3.48 (t, *J* = 5.6 Hz, 2H), 2.81 (t, *J* = 6.4 Hz, 2H), 2.61 (s, 3H), 1.97 – 1.91 (m, 2H), 1.19 (s, 9H). **^{13}C NMR** (75 MHz, Chloroform-*d*) δ 200.3, 148.7, 136.7, 135.7, 135.1, 133.9, 129.4, 127.7, 123.0, 115.9, 110.0, 106.9, 86.0, 41.1, 28.0, 27.6, 27.3, 20.3, 18.8. **IR**: ν 3326, 2931, 2856, 2324, 2136, 1636, 1520, 702 cm^{-1}. seems no ketone on IR spectrum **HRMS** (ESI) m/z: $[M+H]^+$ calculated for $C_{29}H_{31}NOSi^+$ 438.2253; found: 438.2251.

A parallel reaction without ligand was also performed, and no product was observed by ^{1}H NMR analysis with CH_2Br_2 as internal standard.

8'-((*tert*-Butyldiphenylsilyl)ethynyl)-2',3',4',4a'-tetrahydro-1'*H*,6'*H*-spiro[indene-2,5'-pyrido[1,2-a]quinoline]-1,3-dione (2t)

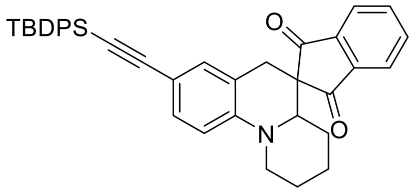

2t

General procedure was followed using 1-(1,2,3,4-tetrahydroquinolin-8-yl)ethan-1-one (**1t**) (53.9 mg, 0.2 mmol, 1.0 equiv.) and *tert*-butyl(iodoethynyl)diphenylsilane (156.2 mg, 0.4 mmol, 2.0 equiv.) in $CHCl_3$ (1.0 mL, 0.2 M) at 80 °C, providing **2t** in 57% ^{1}H NMR yield. Purification by column chromatography on silica gel using pentane: Et_2O (5:1 v/v) as an eluent provided the titled compound as a brown solid (53.5 mg, 50% yield). **^{1}H NMR** (400 MHz, Chloroform-*d*) δ 8.03 – 7.93 (m, 2H), 7.88 – 7.84 (m, 6H), 7.43 – 7.34 (m, 7H), 7.19 – 7.18 (m, 1H), 6.92 (d, *J* = 8.8 Hz, 1H), 4.16 (dt, *J* = 12.8, 2.8 Hz, 1H), 3.45 (dd, *J* = 11.7, 2.4 Hz, 1H), 3.20 (d, *J* = 16.1 Hz, 1H), 2.87 (td, *J* = 12.6, 2.6 Hz, 1H), 2.78 (d, *J* = 16.2 Hz, 1H), 1.76 – 1.71 (m, 2H), 1.66 – 1.55 (m, 1H), 1.50 – 1.46 (m, 1H), 1.40 – 1.35 (m, 1H), 1.18 (dd, *J* = 12.3, 3.3 Hz, 1H), 1.12 (s, 9H). **^{13}C NMR** (126 MHz, Chloroform-*d*) δ 201.9, 199.6, 146.4, 142.3, 141.3, 136.3, 135.8, 134.1, 133.1, 132.1, 129.4, 127.8, 123.6, 123.5, 120.2, 112.9, 111.4, 110.5, 86.6, 59.4, 55.0, 48.8, 32.8, 28.5, 27.3, 25.0, 24.3, 18.9. **IR**: ν 2930, 2855, 2142, 1707, 1607, 1503, 1259, 702 cm^{-1}. **HRMS** (ESI) m/z: $[M+H]^+$ calculated for $C_{39}H_{37}NO_2Si^+$ 580.2672; found: 580.2680; M.p. 76 – 78 °C.

A parallel reaction without ligand was also performed, and no product was observed by ^{1}H NMR analysis with CH_2Br_2 as internal standard.

6. Derivatization of alkynylated products

Procedure for the synthesis of *N,N*-dibenzyl-4-ethynyl-3-fluoroaniline (3). [15]

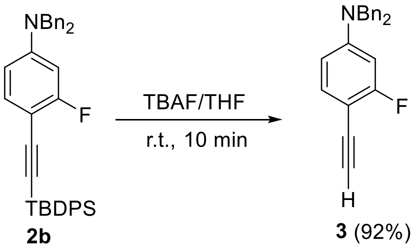

To a solution of alkynylated product **2b** (110.8 mg, 0.2 mmol, 1.0 equiv.) in THF (1.0 mL) and H_2O (50 μL) was added TBAF in THF (1 M, 3.0 mL, 15.0 equiv.) at room temperature and stirred for 10 minutes (reaction time should be extended for a larger scale synthesis). H_2O (5.0 mL) was added and the mixture was extracted three times with EtOAc. The combined organic layers were washed with brine, dried over anhydrous $MgSO_4$, filtered and concentrated under reduced pressure. Purification by column chromatography on silica gel using pentane: Et_2O (4:1 v/v) as an eluent provided the titled compound as a pale yellow solid (58.2 mg, 92%).

^{1}H NMR (400 MHz, Chloroform-*d*) δ 7.28 – 7.27 (m, 1H), 7.24 – 7.10 (m, 10H), 6.345 (s, 1H), 6.341 (dd, *J* = 22.8, 2.8 Hz, 1H), 4.57 (s, 4H), 3.08 (s, 1H). **^{13}C NMR** (101 MHz, Chloroform-*d*) δ 164.7 (d, *J* = 249.5 Hz), 151.2 (d, *J* = 10.9 Hz), 137.2, 134.5 (d, *J* = 3.7 Hz), 128.9, 127.3, 126.4, 108.0 (d, *J* = 2.2 Hz), 99.2 (d, *J* = 26.1 Hz), 97.8 (d, *J* = 16.4 Hz), 79.8 (d, *J* = 2.8 Hz), 78.1, 54.2. **IR**: ν 3289, 3029, 2105, 1623, 1550, 735, 698 cm^{-1}. **HRMS** (FD) m/z: $[M]^+$ calculated for $C_{22}H_{18}FN^+$ 315.1423; found: 315.1438; M.p. 115 – 117 °C.

Procedure for the synthesis of *N,N*-dibenzyl-3-fluoro-4-(phenylethynyl)aniline (4).[16]

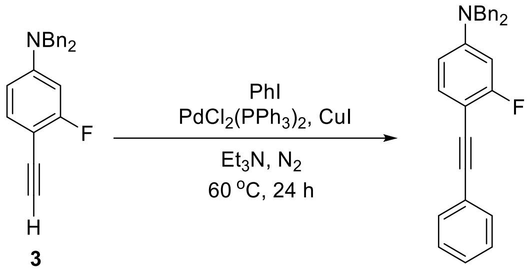

3

4 (80%)

A mixture of iodobenzene (12.3 µL, 0.11 mmol, 1.1 equiv.), *N,N*-dibenzyl-4-ethynyl-3-fluoroaniline (31.5 mg, 0.1 mmol, 1.0 equiv.), $PdCl_2(PPh_3)_2$ (3.5 mg, 5 mol%), CuI (1.9 mg, 10 mol%) and Et_3N (34.8 µL, 2.5 equiv.) was dissolved in THF (2.0 mL). The mixture was degassed and stirred under nitrogen atmosphere at 60 °C for 24 hours. The mixture was diluted with CH_2Cl_2, filtered through a plug of Celite and concentrated under reduced pressure. Purification by column chromatography on silica gel using pentane: Et_2O (4:1 v/v) as an eluent provided the titled compound as a white solid (31.2 mg, 80%).

^{1}H NMR (400 MHz, Chloroform-*d*) δ 7.30 – 7.28 (m, 2H), 7.16 – 7.07 (m, 10H), 7.02 – 7.00 (m, 4H), 6.261 (s, 1H), 6.256 (dd, *J* = 24.0, 2.8 Hz, 1H), 4.46 (s, 4H). **^{13}C NMR** (101 MHz, Chloroform-*d*) δ 164.0 (d, *J* = 249.5 Hz), 150.8 (d, *J* = 10.1 Hz), 137.3, 134.0 (d, *J* = 3.0 Hz), 131.4, 128.9, 128.2, 127.8, 127.3, 126.5, 123.8, 108.1 (d, *J* = 3.0 Hz), 99.4 (d, *J* = 26.3 Hz), 99.1, 92.1 (d, *J* = 3.0 Hz), 83.8, 54.2. **HRMS** (ESI) m/z: $[M+H]^+$ calculated for $C_{28}H_{22}FN^+$ 392.1815; found: 392.1817; M.p. 151 – 153 °C.

Procedure for the synthesis of *N,N*-dibenzyl-4-(1-benzyl-1*H*-1,2,3-triazol-4-yl)-3-fluoroaniline (5).

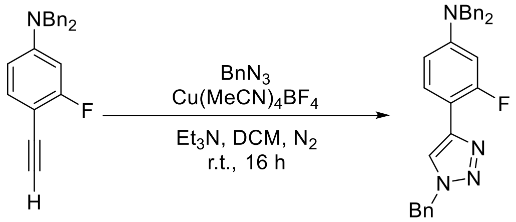

3

5 (66%)

To a solution of *N,N*-dibenzyl-4-ethynyl-3-fluoroaniline (31.5 mg, 0.1 mmol, 1.0 equiv.) and benzyl azide (28.0 mg, 0.15 mmol, 1.5 equiv.) in CH_2Cl_2 (2.5 mL) was added Et_3N (20.9 µL, 0.15 mmol, 1.5 equiv.) and CuI (9.4 mg, 0.03 mmol, 30 mol%). The mixture was degassed and stirred under nitrogen atmosphere at room temperature for 16 hours. The mixture was diluted with CH_2Cl_2, filtered through a plug of Celite and concentrated under reduced pressure. Purification by column chromatography on silica gel using pentane: Et_2O (1:1 v/v) as an eluent provided the titled compound as a white solid (29.5 mg, 66%).

^{1}H NMR (400 MHz, Chloroform-*d*) δ 7.95 (t, *J* = 8.8 Hz, 1H), 7.58 (t, *J* = 3.6 Hz, 1H), 7.27 – 7.13 (m, 15H), 6.56 (dd, *J* = 8.8, 2.4 Hz, 1H), 6.35 (dd, *J* = 14.4, 2.4 Hz, 1H), 5.46 (s, 2H), 4.57 (s, 4H). **^{13}C NMR** (101 MHz, Chloroform-*d*) δ 160.5 (d, *J* = 246.4 Hz), 150.5 (d, *J* = 11.1 Hz), 142.4 (d, *J* = 2.0 Hz), 137.7, 135.0, 129.0, 128.8, 128.6, 128.4 (d, *J* = 5.1 Hz), 127.9, 127.2, 126.6, 120.9 (d, *J* = 11.1 Hz), 108.7 (d, *J* = 2.0 Hz), 106.9 (d, *J* = 14.1 Hz), 99.3 (d, *J* = 27.3 Hz), 54.2, 54.1. **IR**: ν 3030, 1634, 1565, 1501, 1452, 724, 696 cm^{-1}. **HRMS** (ESI) m/z: $[M+H]^+$ calculated for $C_{29}H_{25}FN_4^+$ 449.2141; found: 449.2144; M.p. 158 – 160 °C.

Procedure for the synthesis of *N,N*-dibenzyl-6-((*tert*-butyldiphenylsilyl)ethynyl)-[1,1'-biphenyl]-3-amine (6).[17]

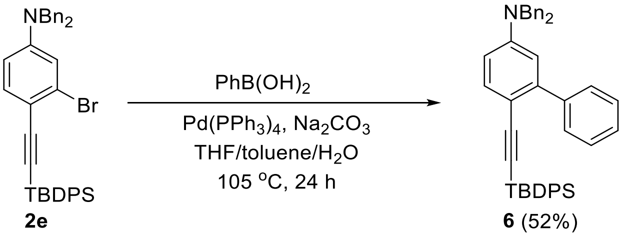

To a solution of alkynylated product **2e** (61.5 mg, 0.1 mmol, 1.0 equiv.) and phenylboronic acid (14.6 mg, 0.12 mmol, 1.2 equiv.) in THF/toluene/H_2O (0.5 mL/0.5 mL/20 μL) was added $Pd(PPh_3)_4$ (8.1 mg, 7 mol%) and sodium carbonate (13.8 mg, 0.13 mmol, 1.3 equiv.). The mixture was degassed and stirred under nitrogen atmosphere at room temperature for 16 hours. The mixture was diluted with CH_2Cl_2, filtered through a plug of Celite and concentrated under reduced pressure. Purification by column chromatography on silica gel using pentane: CH_2Cl_2 (5:1 v/v) as an eluent provided the titled compound as a yellow oil (31.9 mg, 52%).

^{1}H NMR (300 MHz, Chloroform-*d*) δ 7.72 – 7.69 (m, 4H), 7.57 – 7.54 (m, 3H), 7.40 – 7.26 (m, 16H), 7.27 – 7.26 (m, 2H), 6.79 (d, *J* = 2.7 Hz, 1H), 6.72 (dd, *J* = 8.7, 2.7 Hz, 1H), 4.73 (s, 4H), 1.01 (s, 9H). **^{13}C NMR** (101 MHz, Chloroform-*d*) δ 149.4, 145.9, 141.2, 137.8, 135.7, 135.2, 134.0, 129.4, 129.2, 128.8, 128.0, 127.5, 127.2, 127.2, 126.6, 113.1, 111.1, 109.8, 109.5, 89.4, 54.1, 27.1, 18.7. **IR**: ν 3067, 2928, 2855, 2147, 1599, 1493, 1109, 764, 734, 698 cm^{-1}. **HRMS** (FD) m/z: $[M]^+$ calculated for $C_{44}H_{41}NSi^+$ 611.3008; found: 611.2999.

Procedure for the synthesis of *N,N*-dibenzyl-4-((*tert*-butyldiphenylsilyl)ethynyl)-3-morpholinoaniline (7).[18]

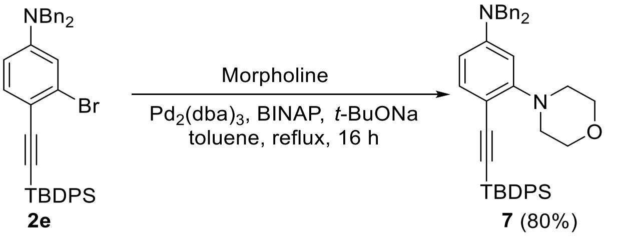

A mixture of alkynylated product (123 mg, 0.2 mmol, 1.0 equiv.), morpholine (87.1 mg, 1.0 mmol, 5.0 equiv.), $Pd_2(dba)_3$ (18.3 mg, 10 mol%), BINAP (24.9 mg, 20 mol%) and *t*-BuONa (25 mg, 0.26 mmol, 1.3 equiv.) was dissolved in toluene (1.0 mL). The mixture was degassed and stirred under nitrogen atmosphere at 115 °C for 16 hours. The mixture was diluted with CH_2Cl_2, filtered through a plug of Celite and concentrated under reduced pressure. Purification by column chromatography on silica gel using pentane: Et_2O (2:1 v/v) as an eluent provided the titled compound as a yellow oil (31.2 mg, 80%).

^{1}H NMR (400 MHz, Chloroform-*d*) δ 7.77 – 7.74 (m, 4H), 7.31 – 7.27 (m, 4H), 7.26 – 7.22 (m, 6H), 7.18 – 7.13 (m, 7H), 6.28 (dd, *J* = 8.8, 2.4 Hz, 1H), 6.12 (d, *J* = 2.4 Hz, 1H), 4.58 (s, 4H), 3.55 (t, *J* = 4.8 Hz, 4H), 2.98 (t, *J* = 4.4 Hz, 4H), 1.03 (s, 9H). **^{13}C NMR** (101 MHz, Chloroform-*d*) δ 155.7, 150.5, 138.1, 136.3, 135.7, 134.1, 129.4, 128.8, 127.7, 127.2, 126.6, 109.2, 106.3, 104.2, 101.7, 92.1, 67.2, 54.6, 51.4, 27.3, 18.7. **IR**: ν 3068, 2956, 2928, 2855, 2140, 1600, 1509, 1112, 738, 699 cm^{-1}. **HRMS** (FD) m/z: $[M]^+$ calculated for $C_{42}H_{44}N_2OSi^+$ 620.3223; found: 620.3218.

7. ^{1}H NMR and ^{13}C NMR spectra

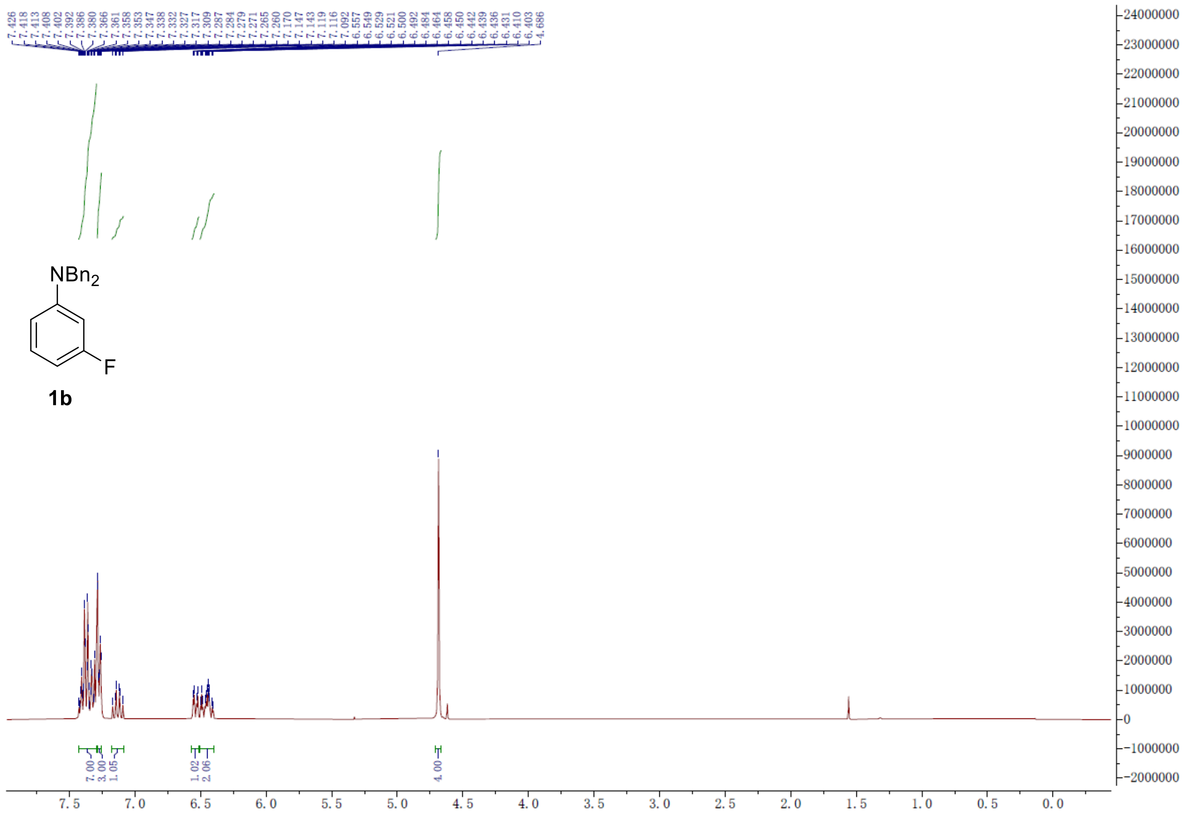

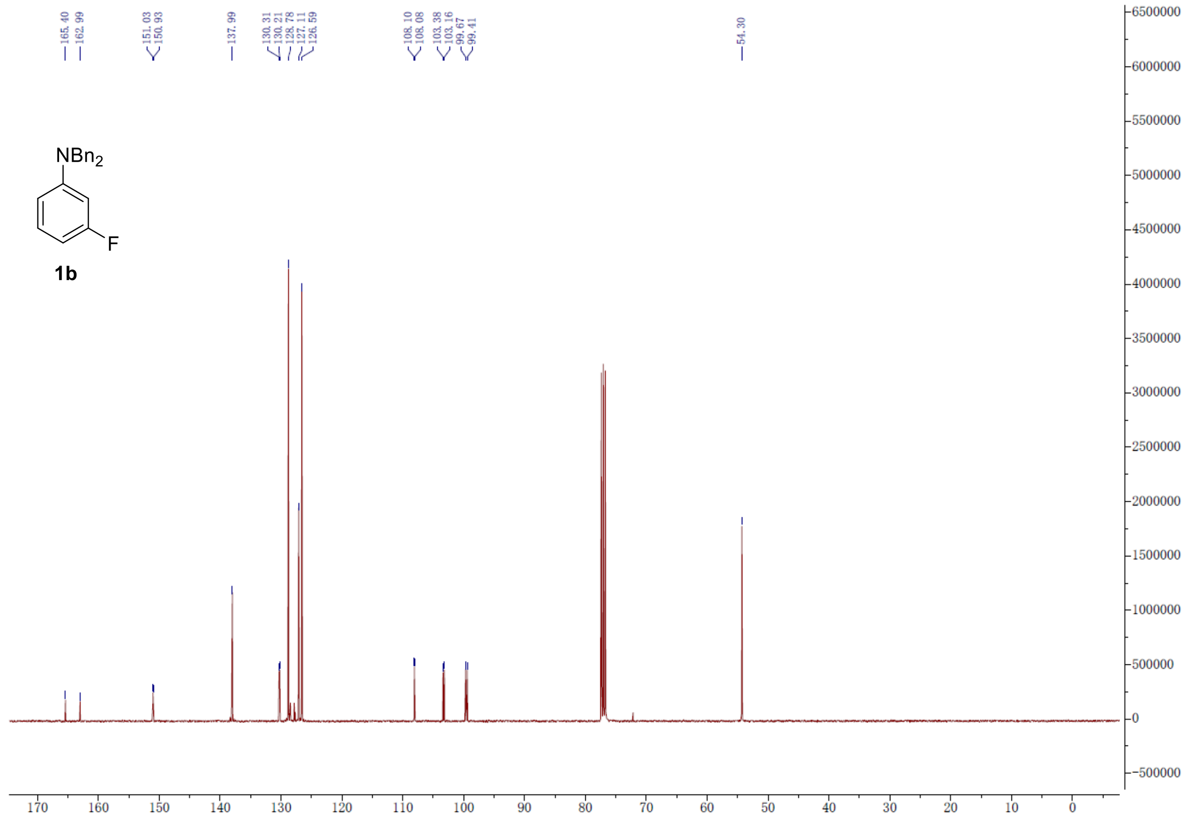

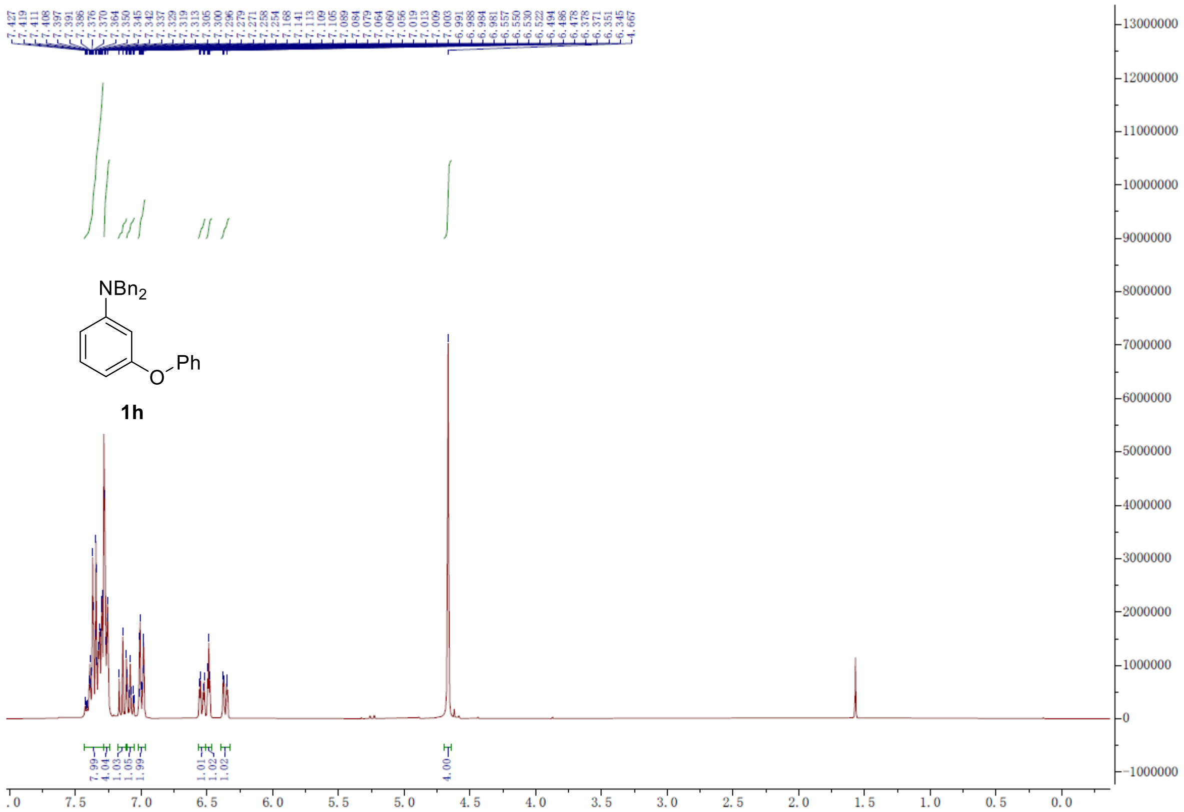

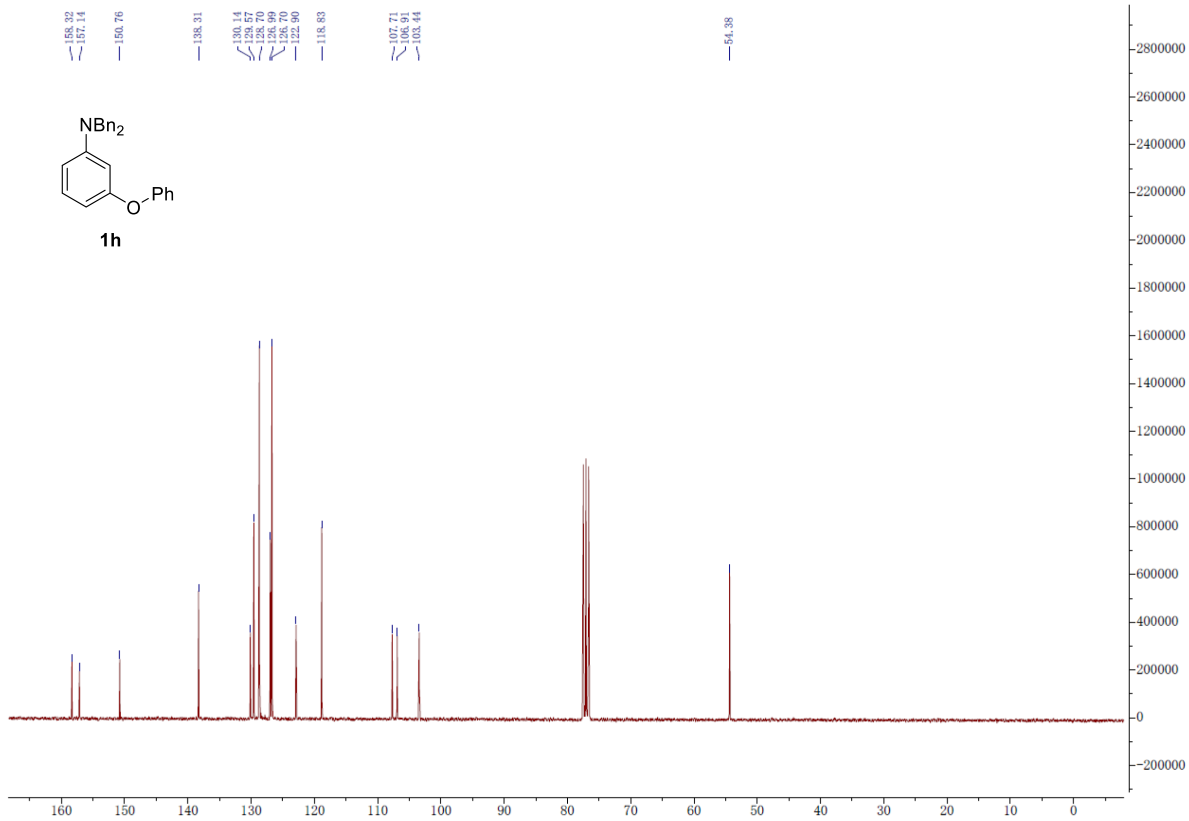

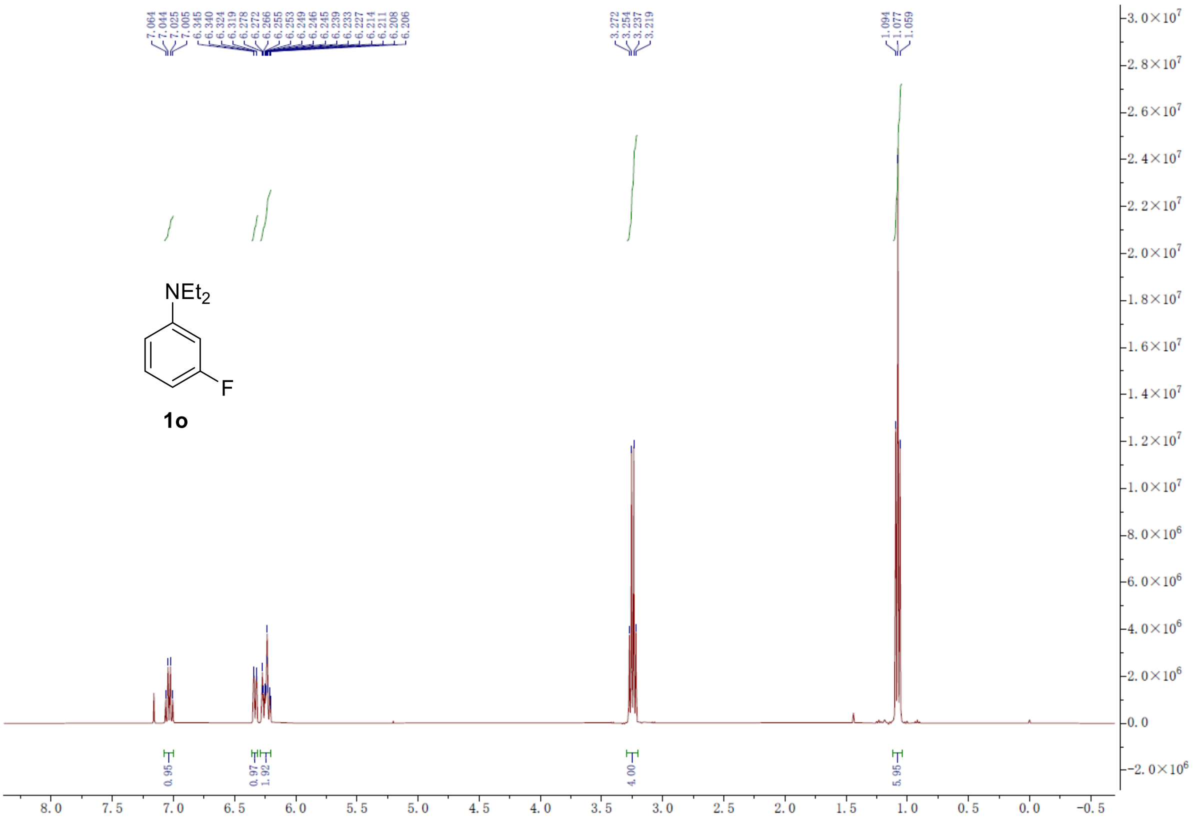

7.064
7.044
7.025
7.005
6.345
6.340
6.324
6.319
6.278
6.272
6.266
6.255
6.253
6.249
6.246
6.245
6.239
6.233
6.227
6.214
6.211
6.208
6.206
3.272
3.254
3.237
3.219
1.094
1.077
1.059
NEt2
F
1o
0.95
0.97
1.92
4.00
5.95

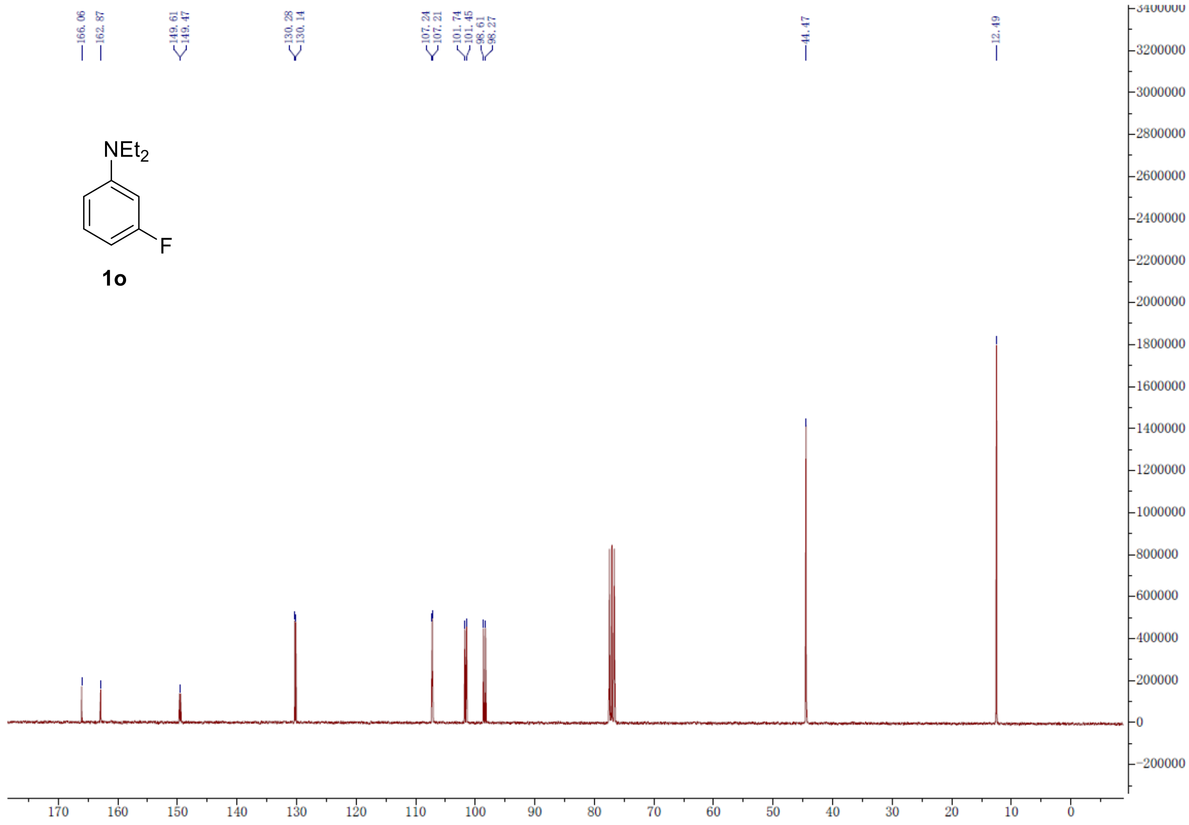

166.06
162.87
149.61
149.47
130.28
130.14
107.24
107.21
101.74
101.45
98.61
98.27
44.47
12.49
NEt2
F
1o

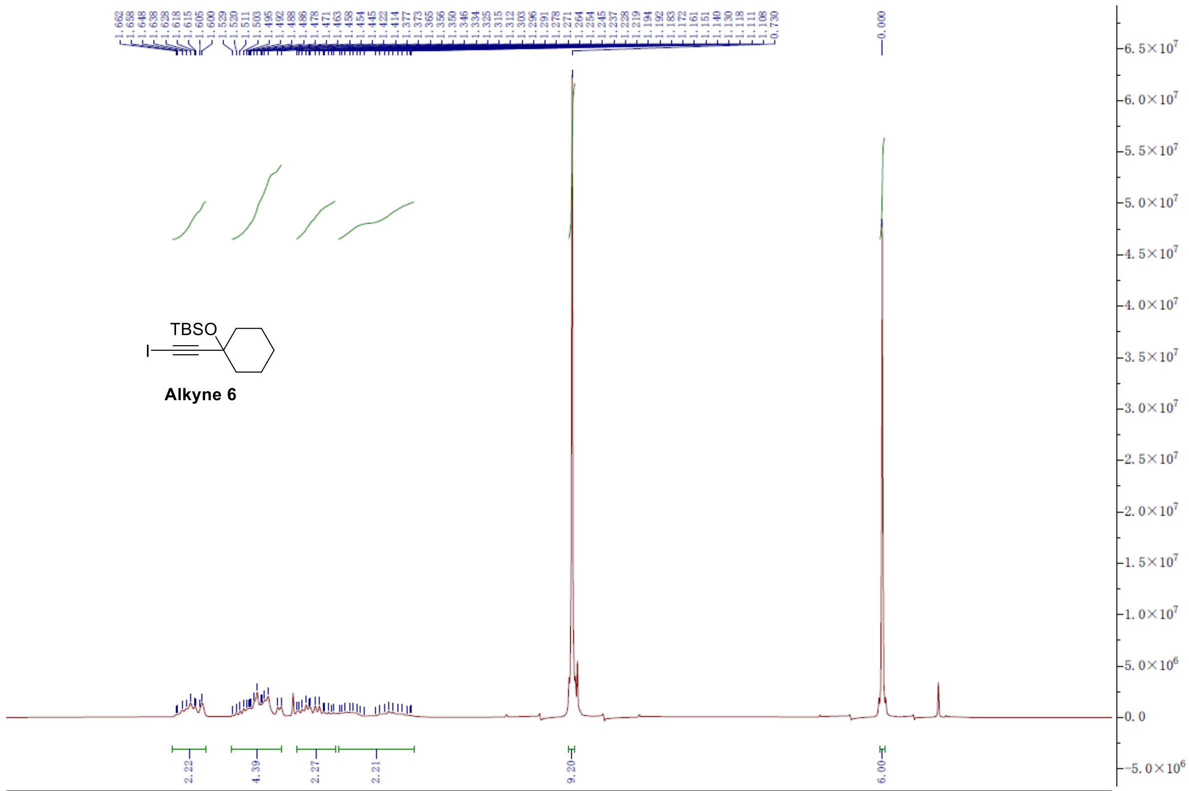

Alkyne 6

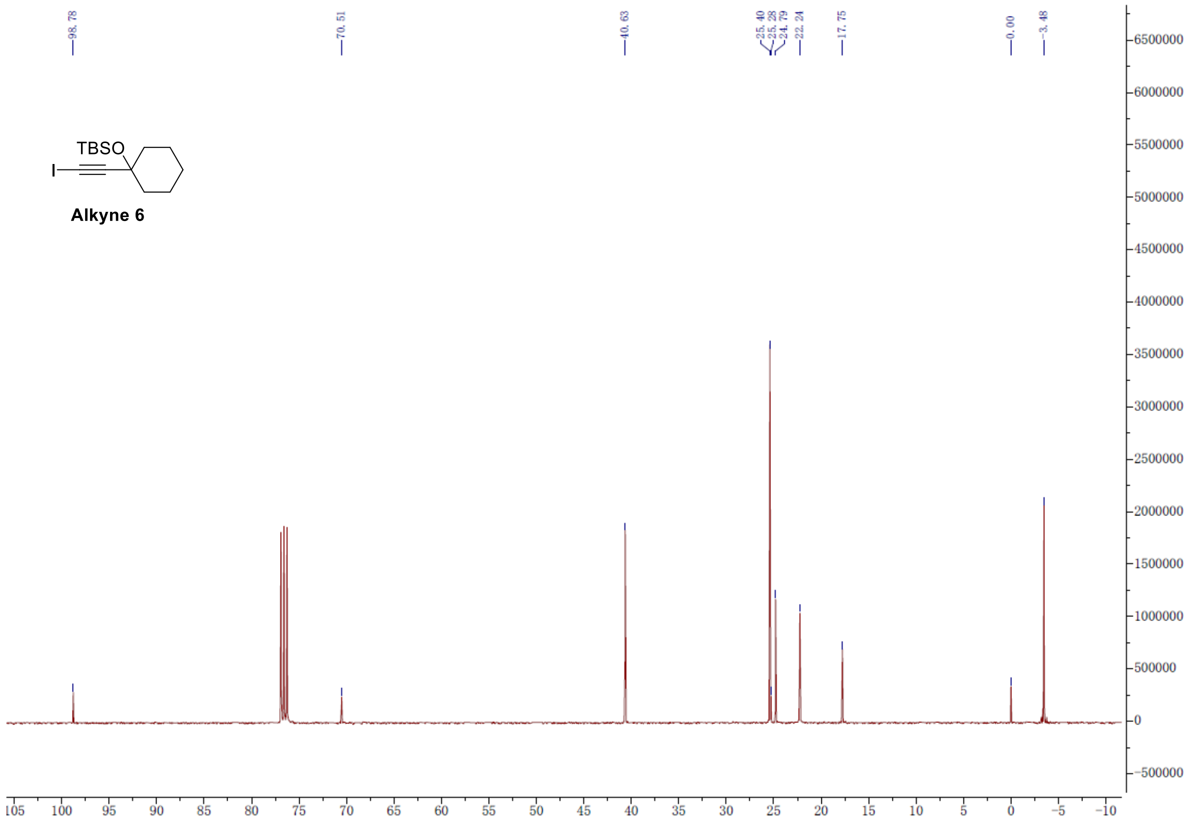

Alkyne 6

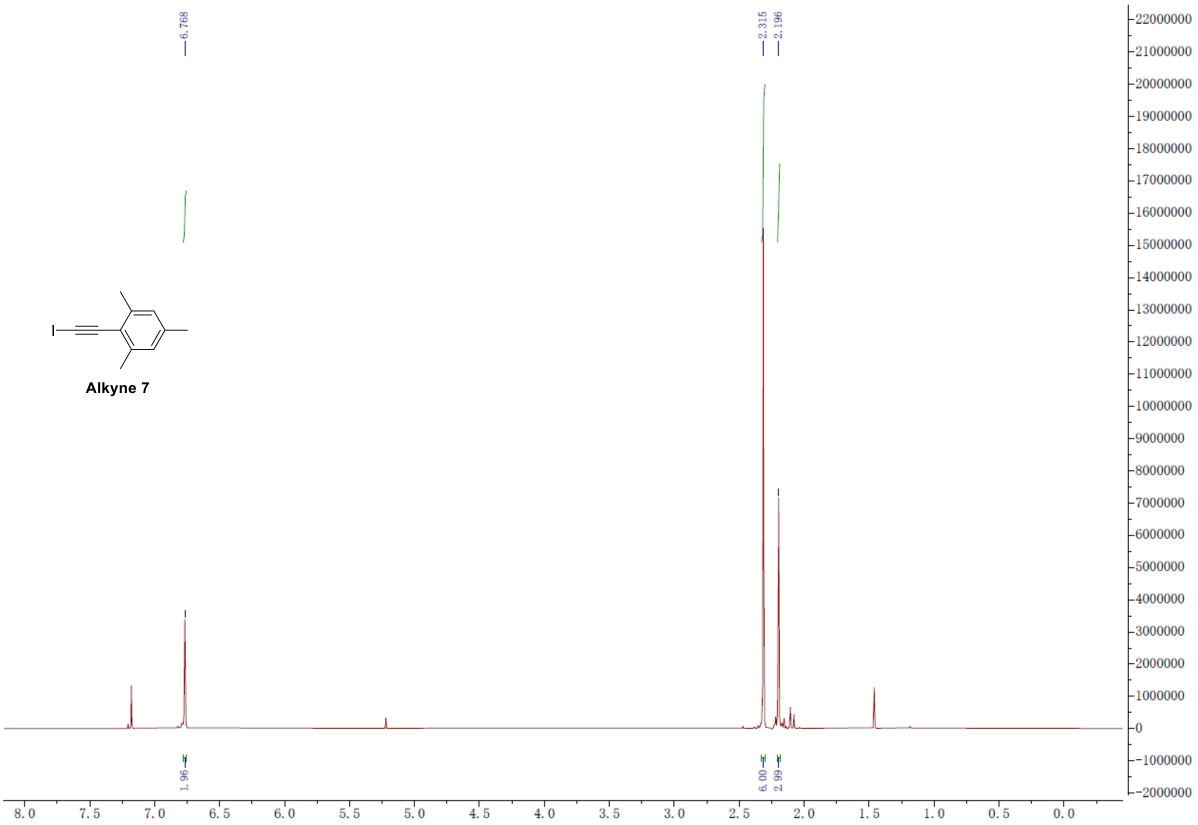
6.768
2.315
2.196
1.96
6.00
2.99
Alkyne 7

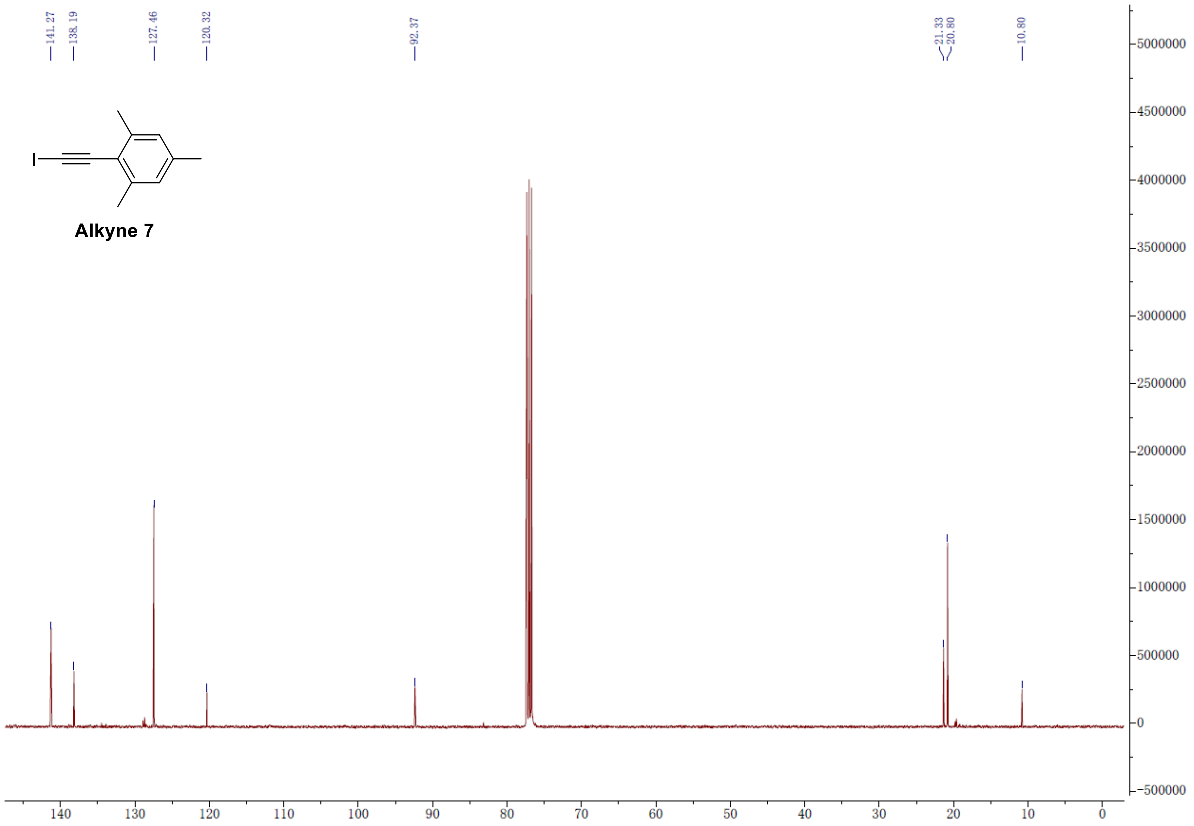
141.27
138.19
127.46
120.32
92.37
21.33
20.80
10.80
Alkyne 7

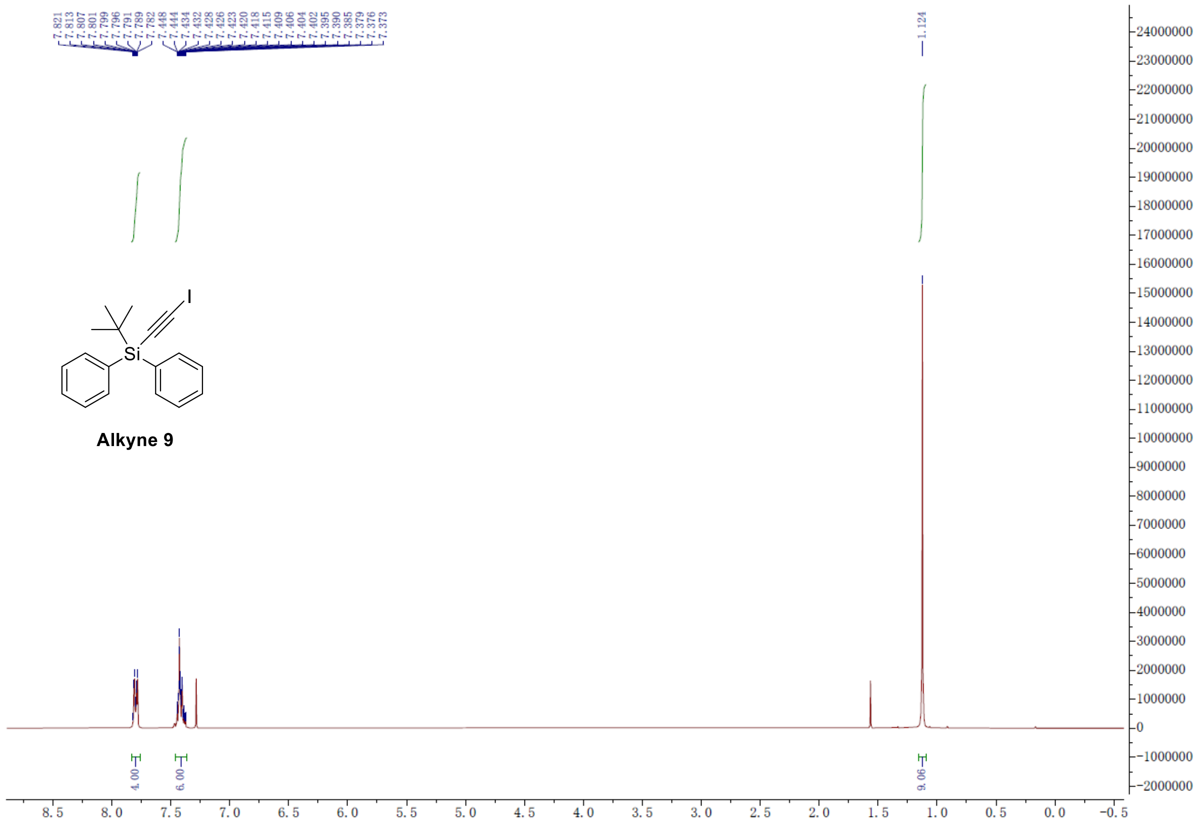
7.821 7.813 7.807 7.801 7.799 7.796 7.791 7.789 7.782 7.448 7.444 7.434 7.432 7.428 7.426 7.423 7.420 7.418 7.415 7.409 7.406 7.404 7.402 7.395 7.390 7.385 7.379 7.376 7.373
1.124
Si
I
Alkyne 9
4.00
6.00
9.06

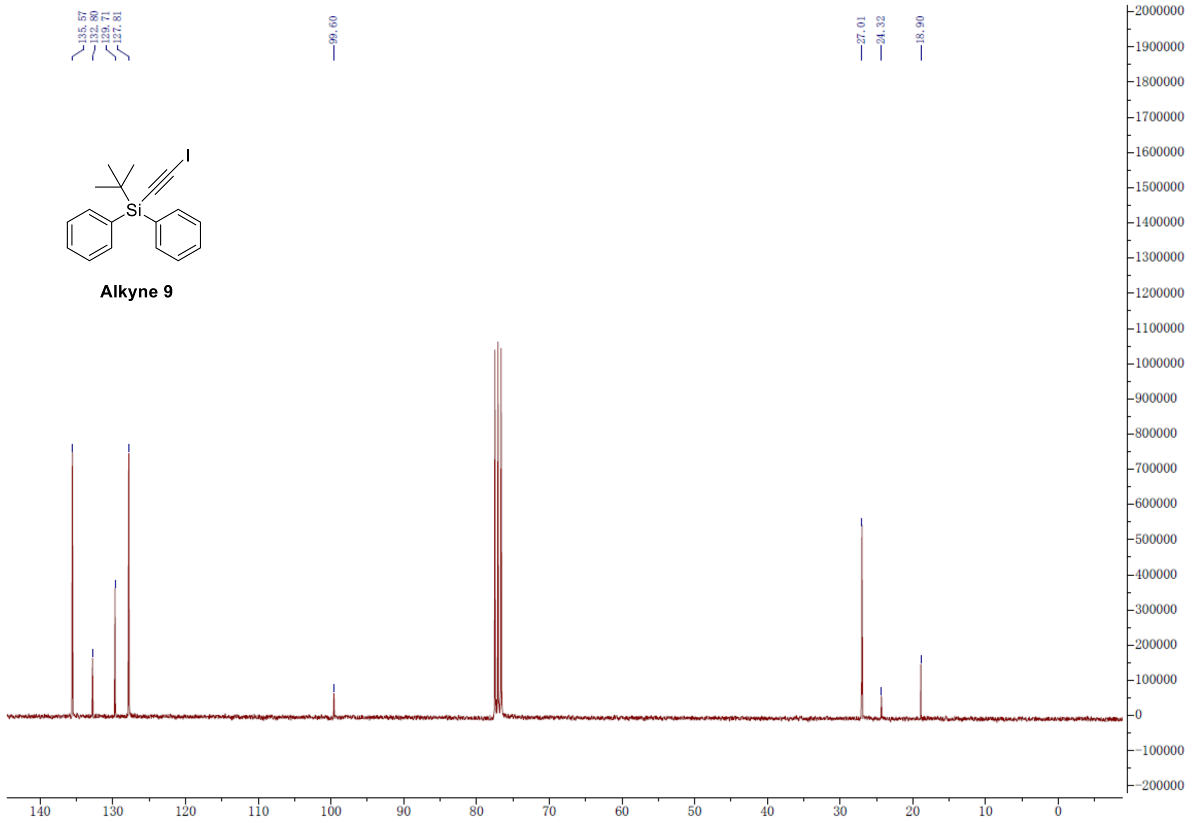
135.57 132.80 129.71 127.81
99.60
27.01 24.32 18.90
Si
I
Alkyne 9

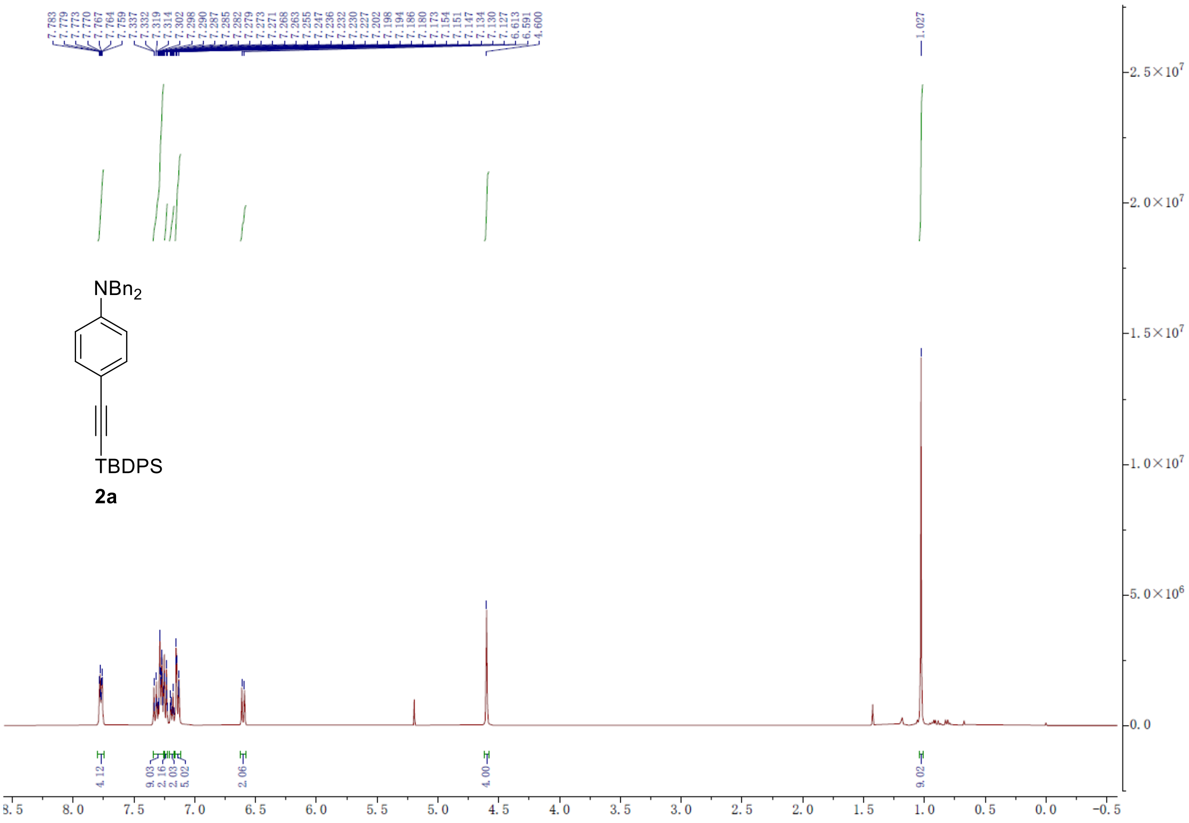

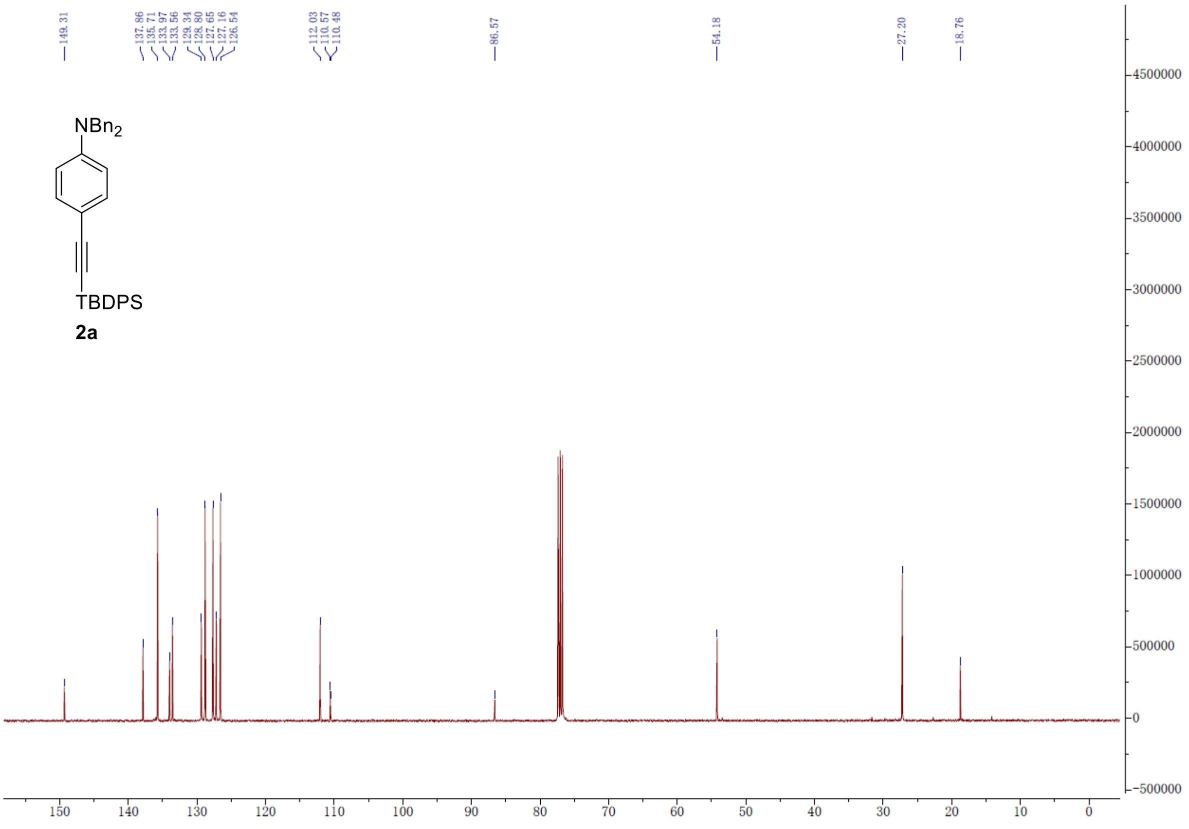

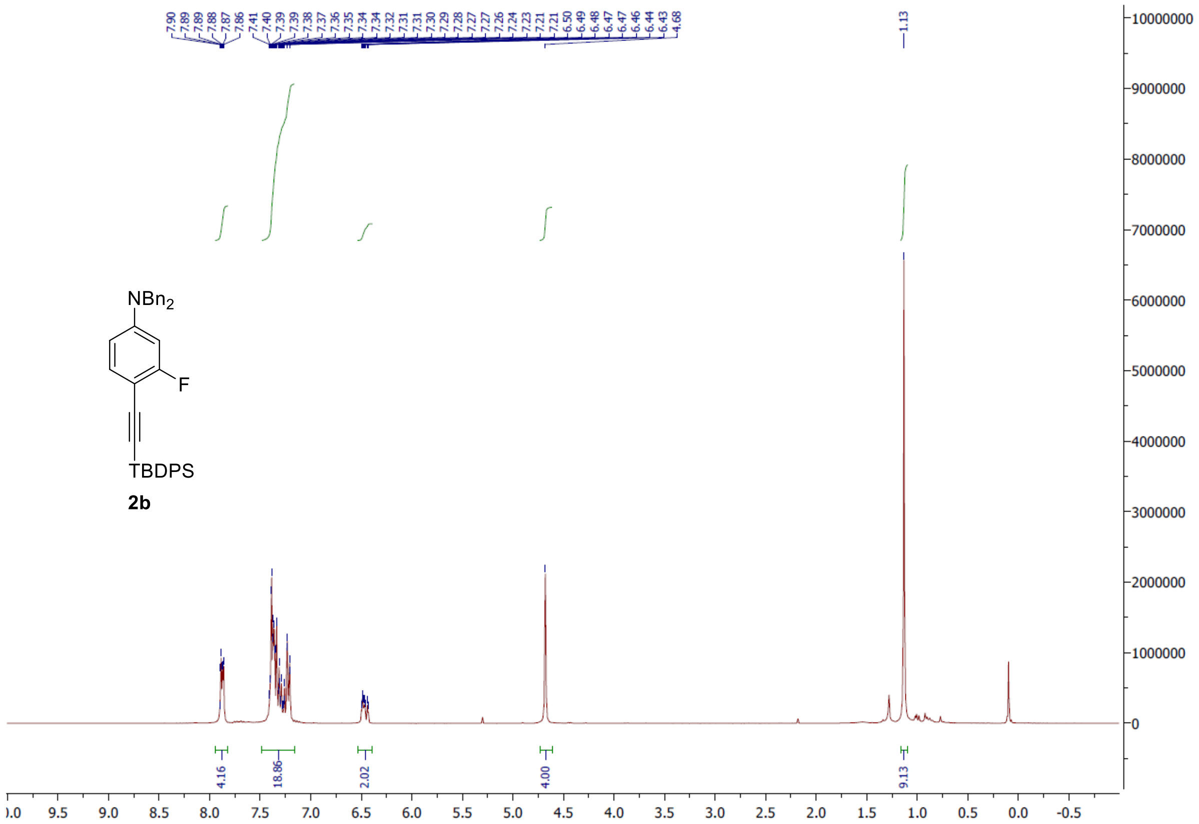
NBn2
F
TBDPS
2b

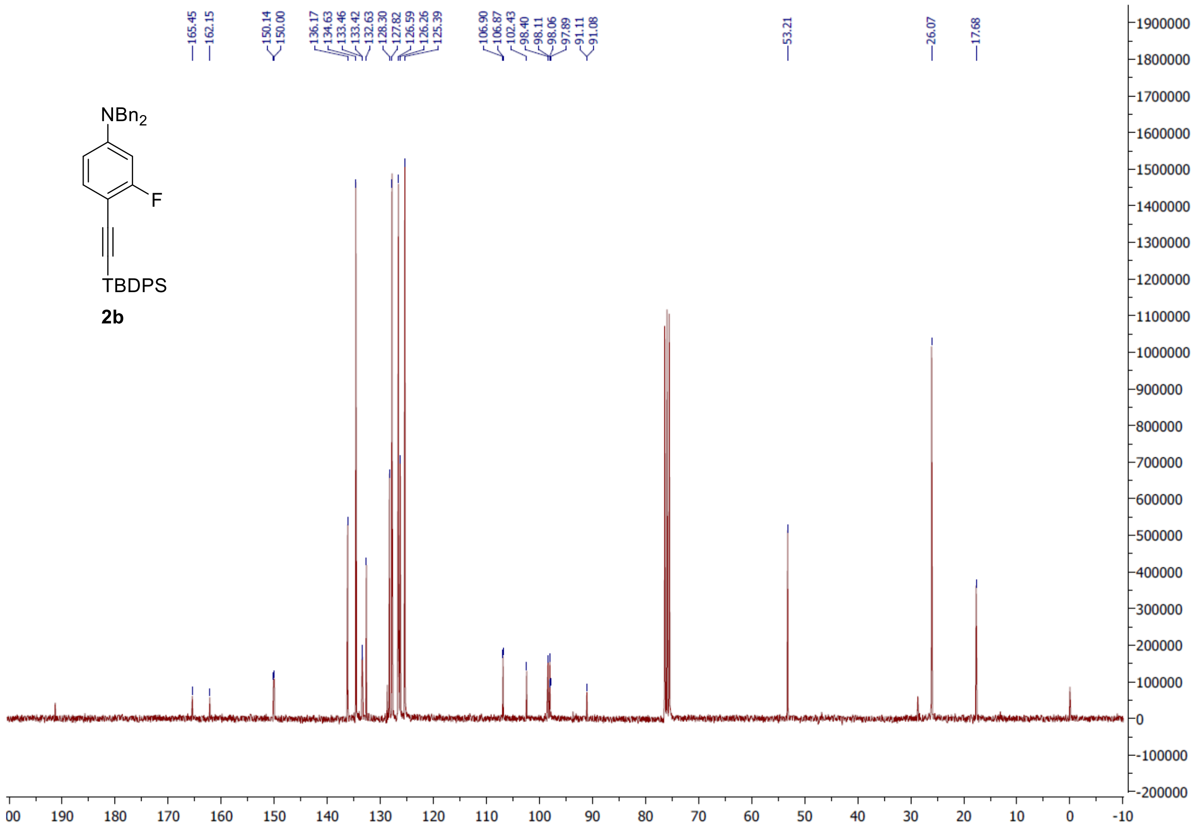
NBn2
F
TBDPS
2b

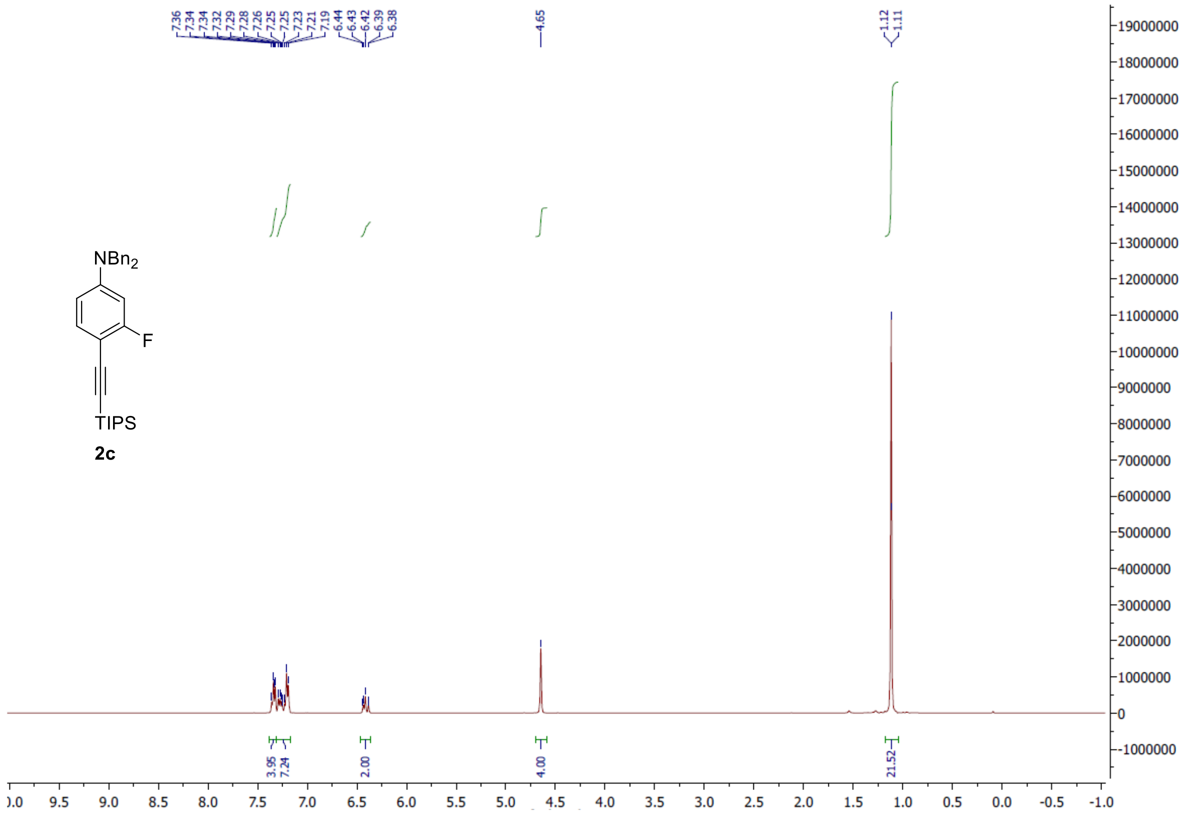

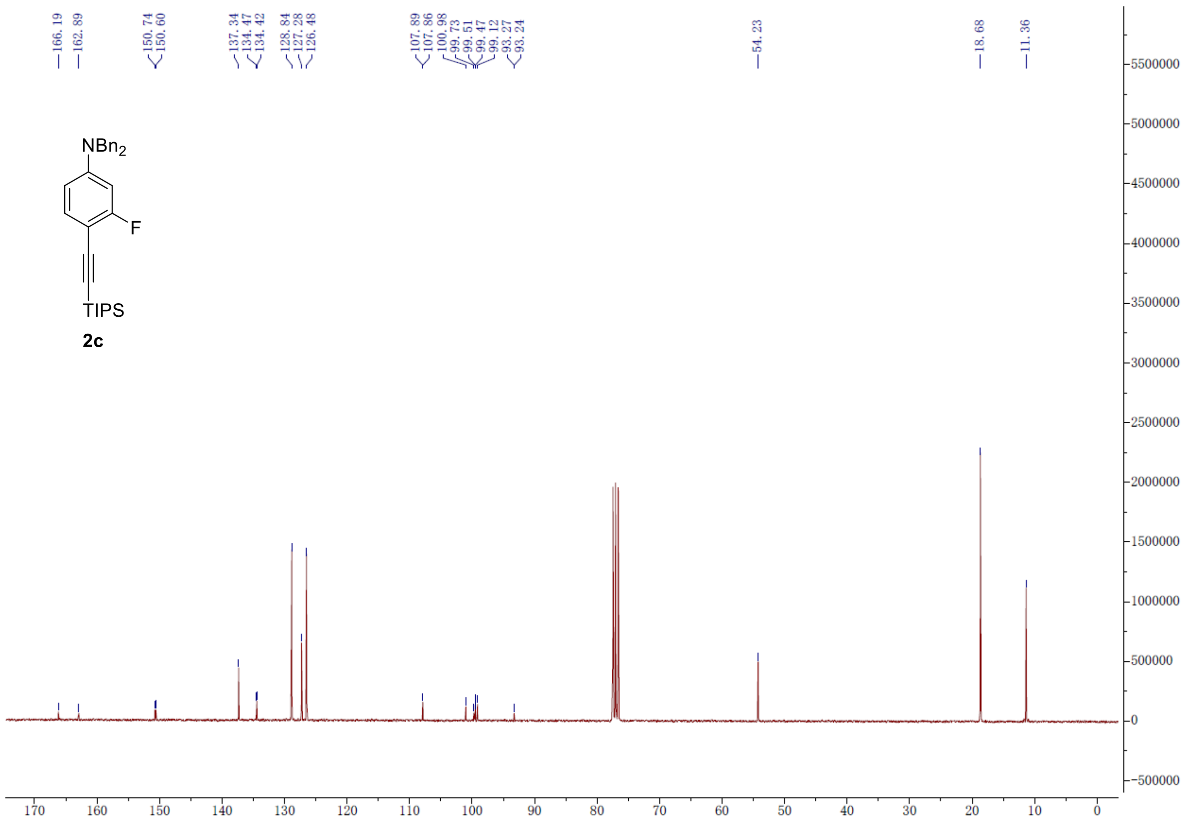

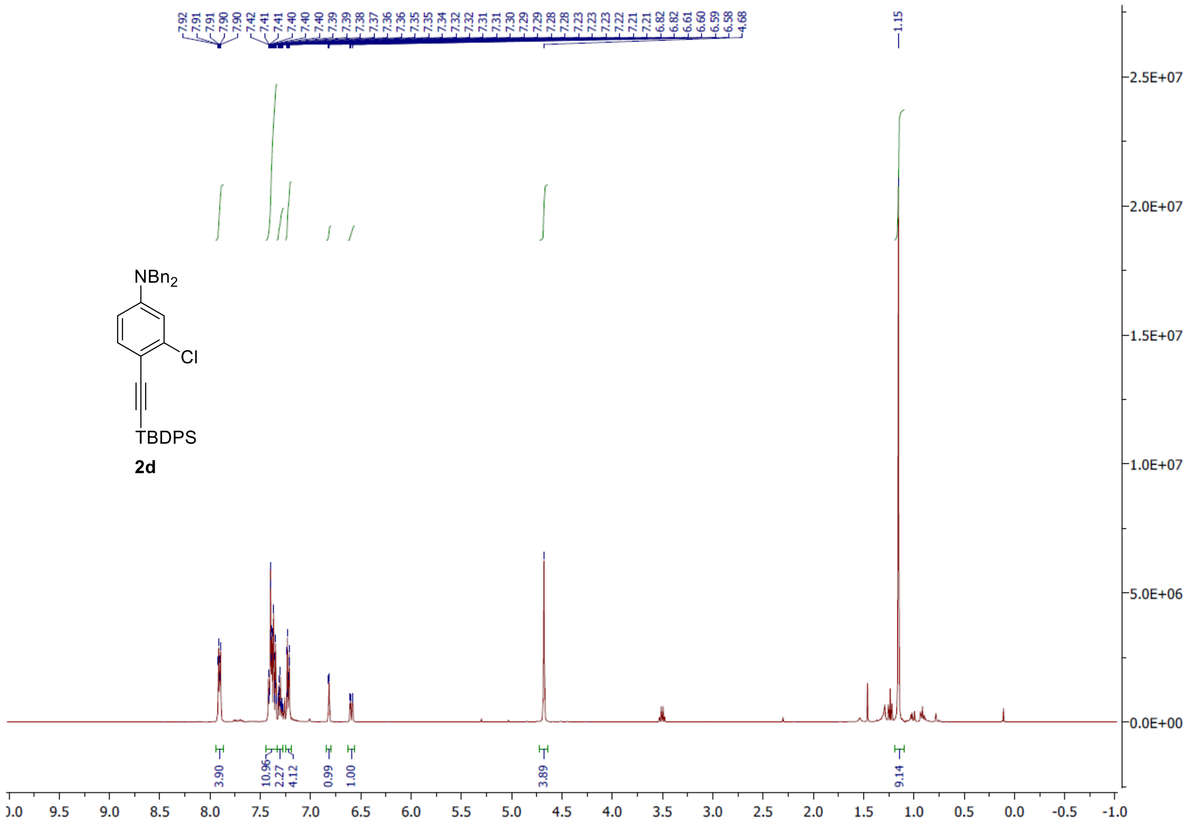

NBn2
Cl
TBDPS
2d

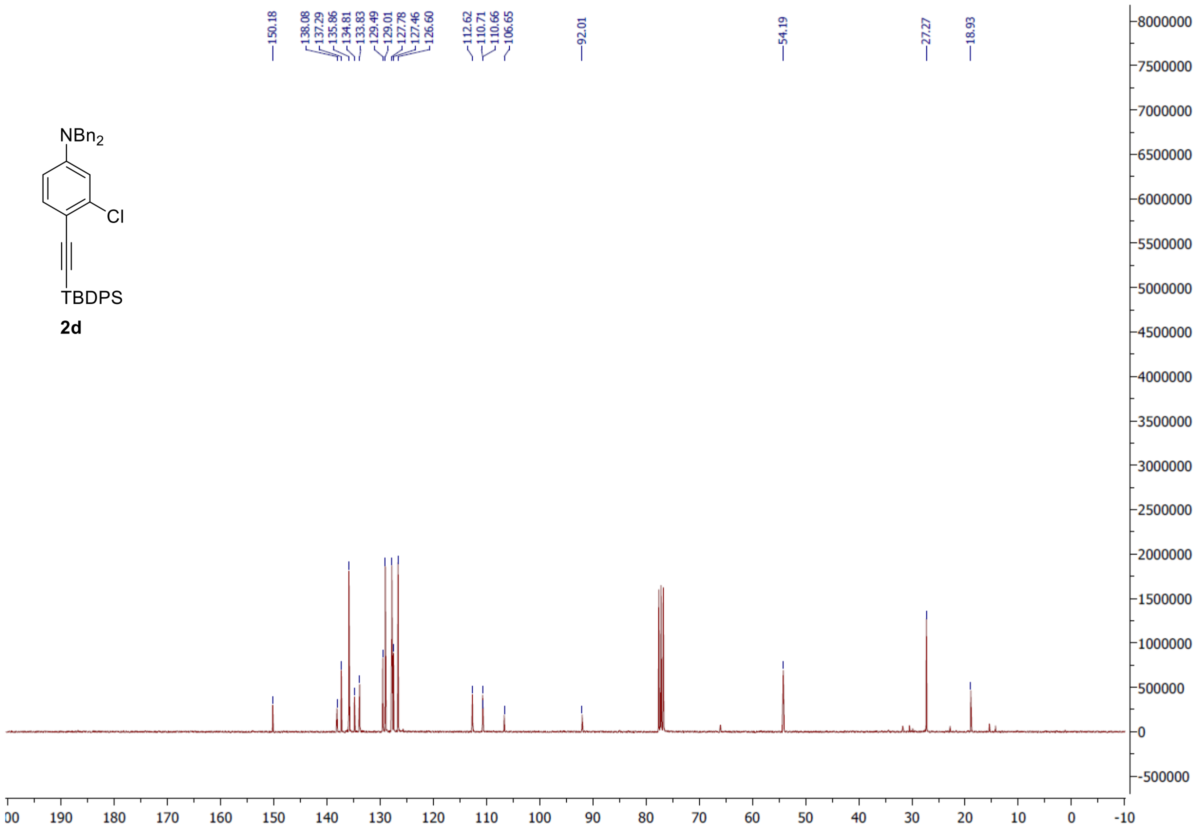

NBn2
Cl
TBDPS
2d

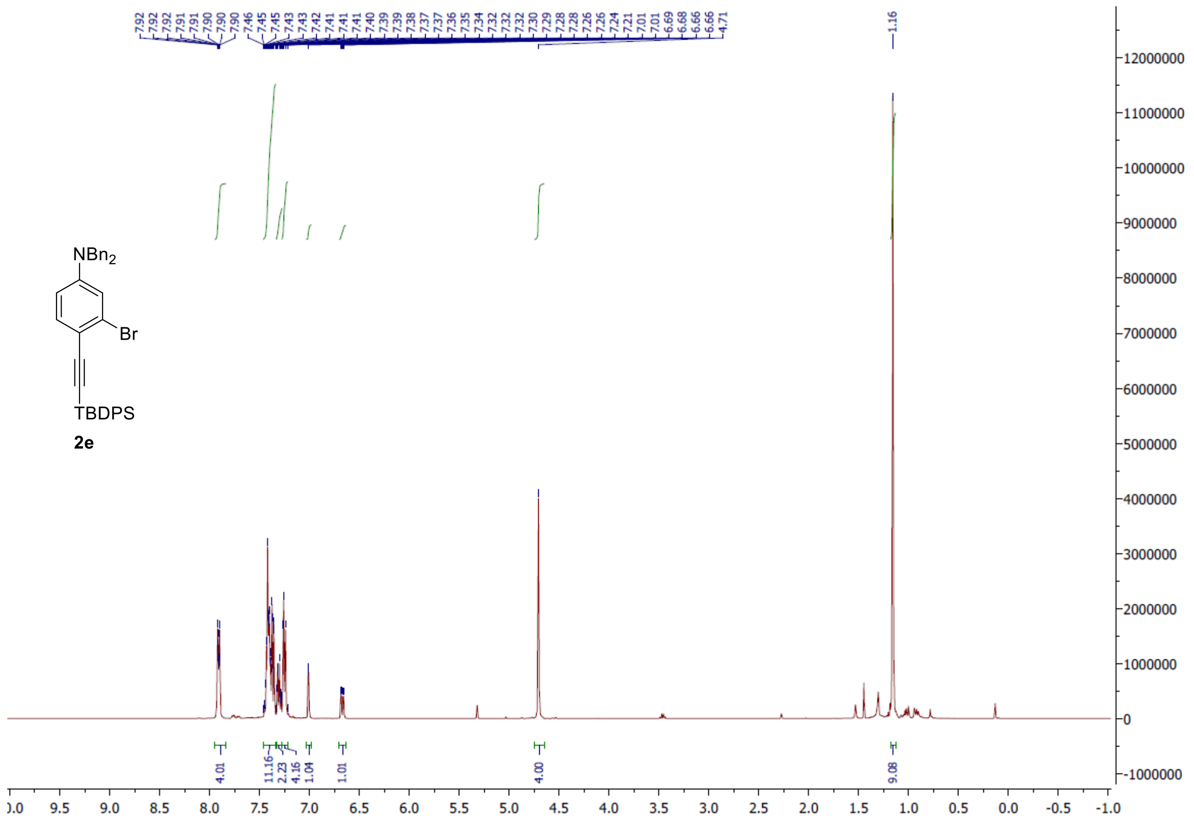
NBn2
Br
TBDPS
2e

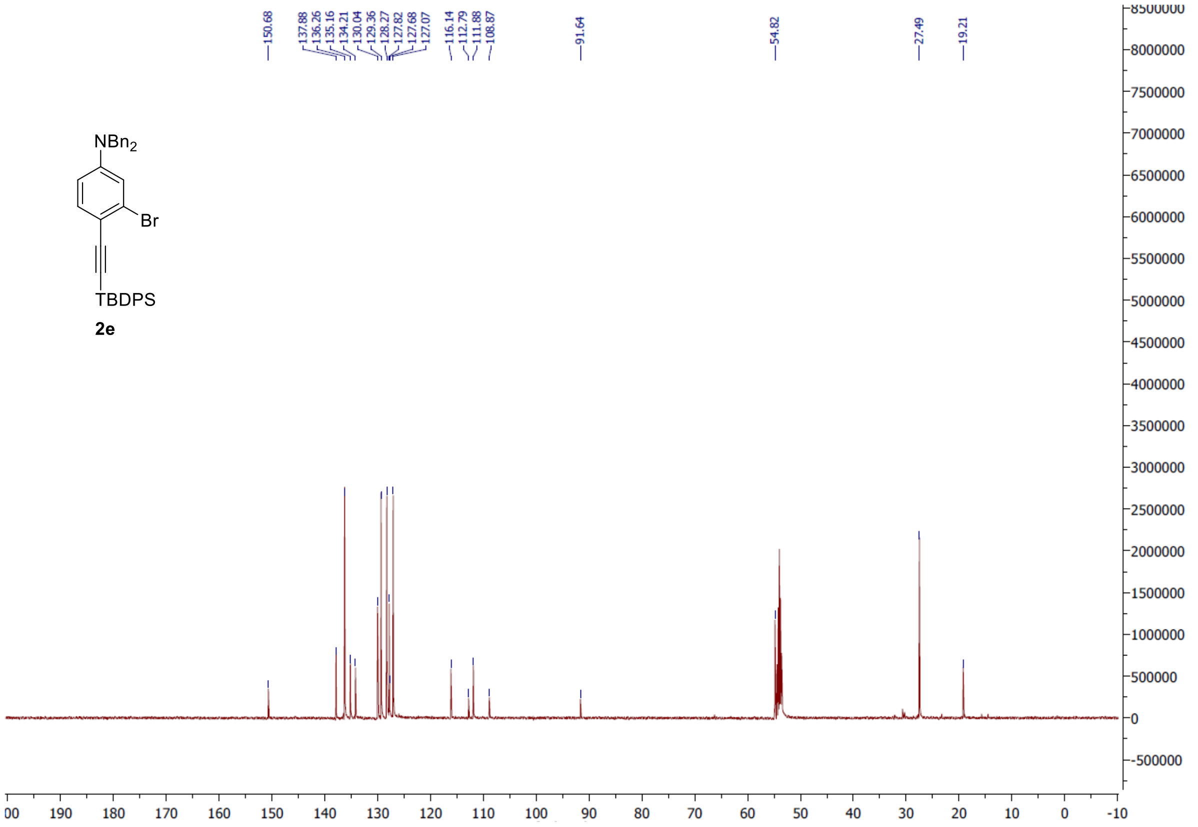
NBn2
Br
TBDPS
2e

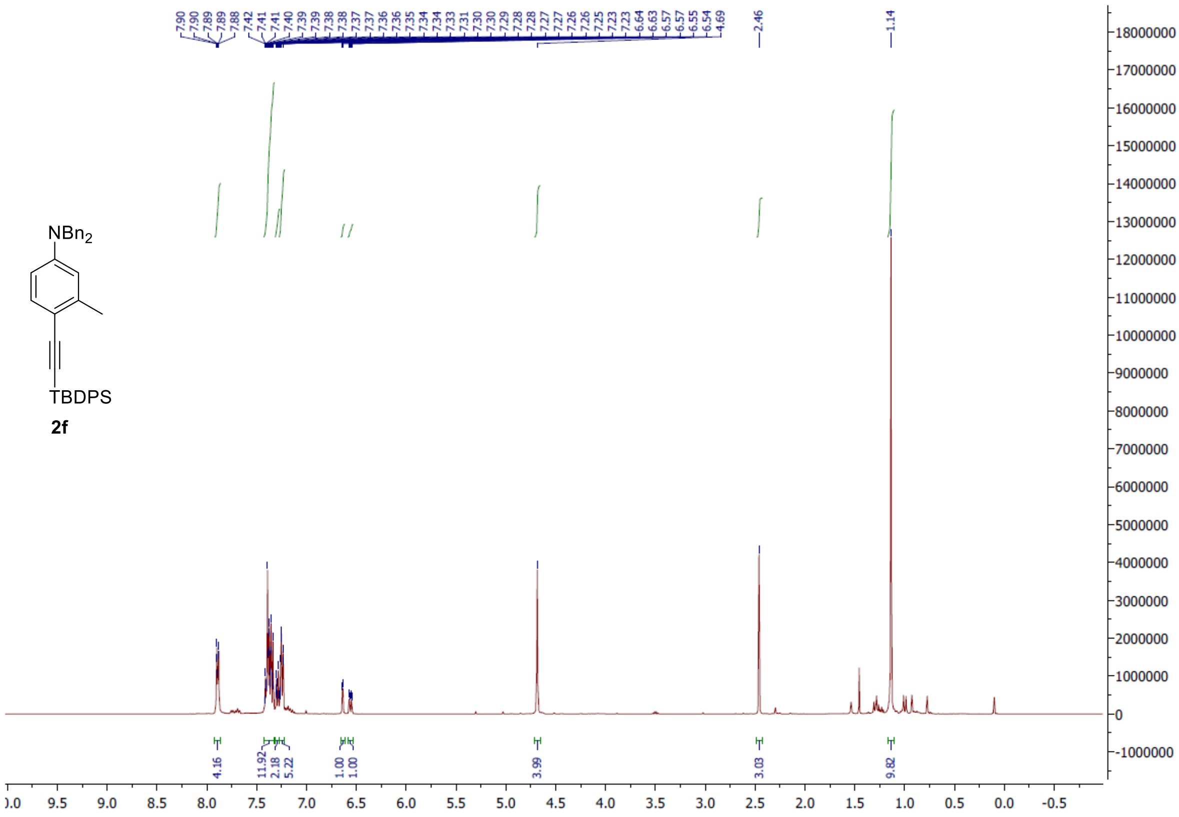
NBn2
TBDPS
2f

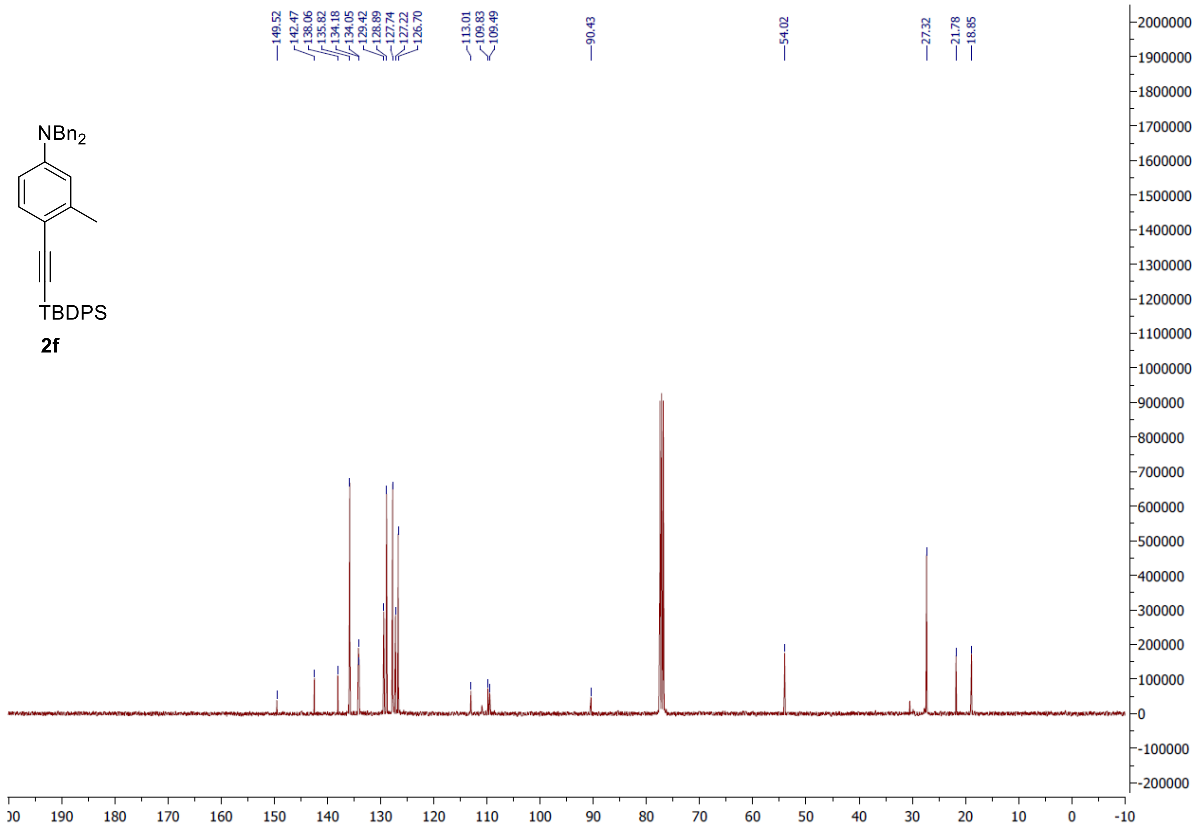
NBn2
TBDPS
2f

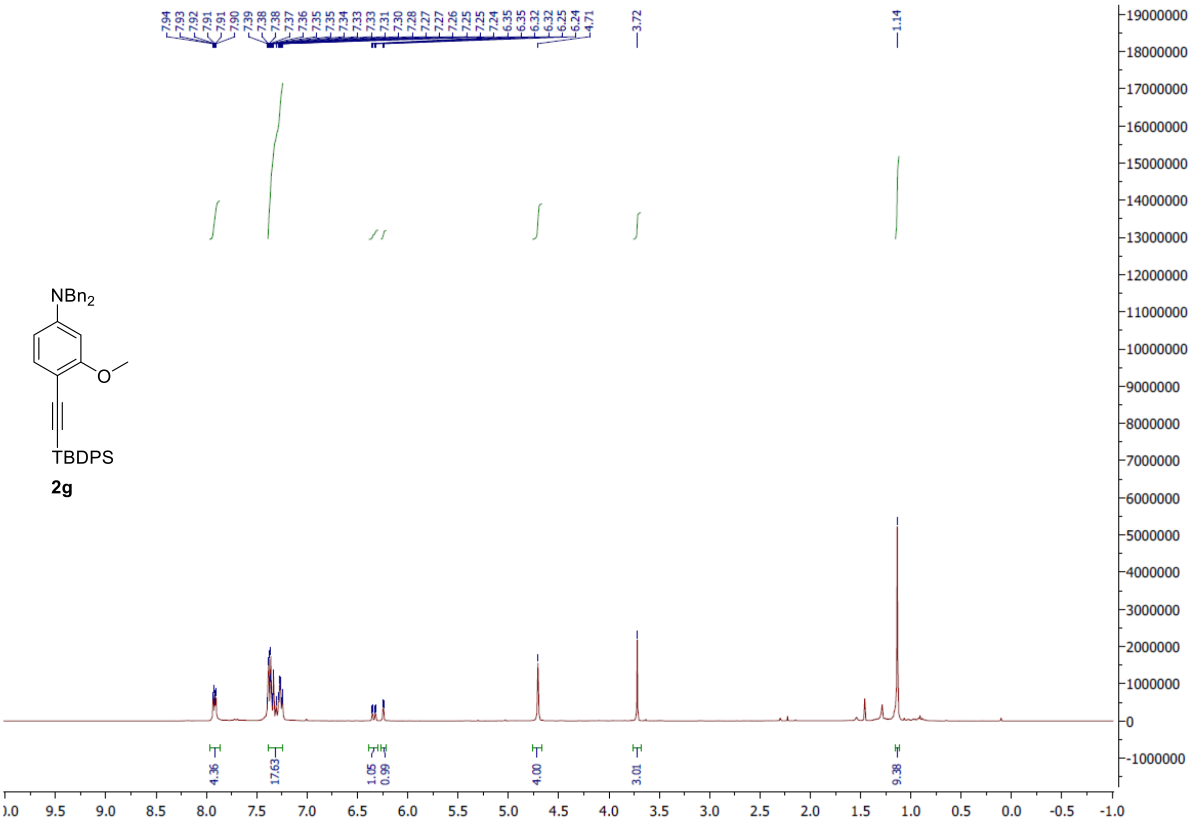
NBn2
O
TBDPS
2g

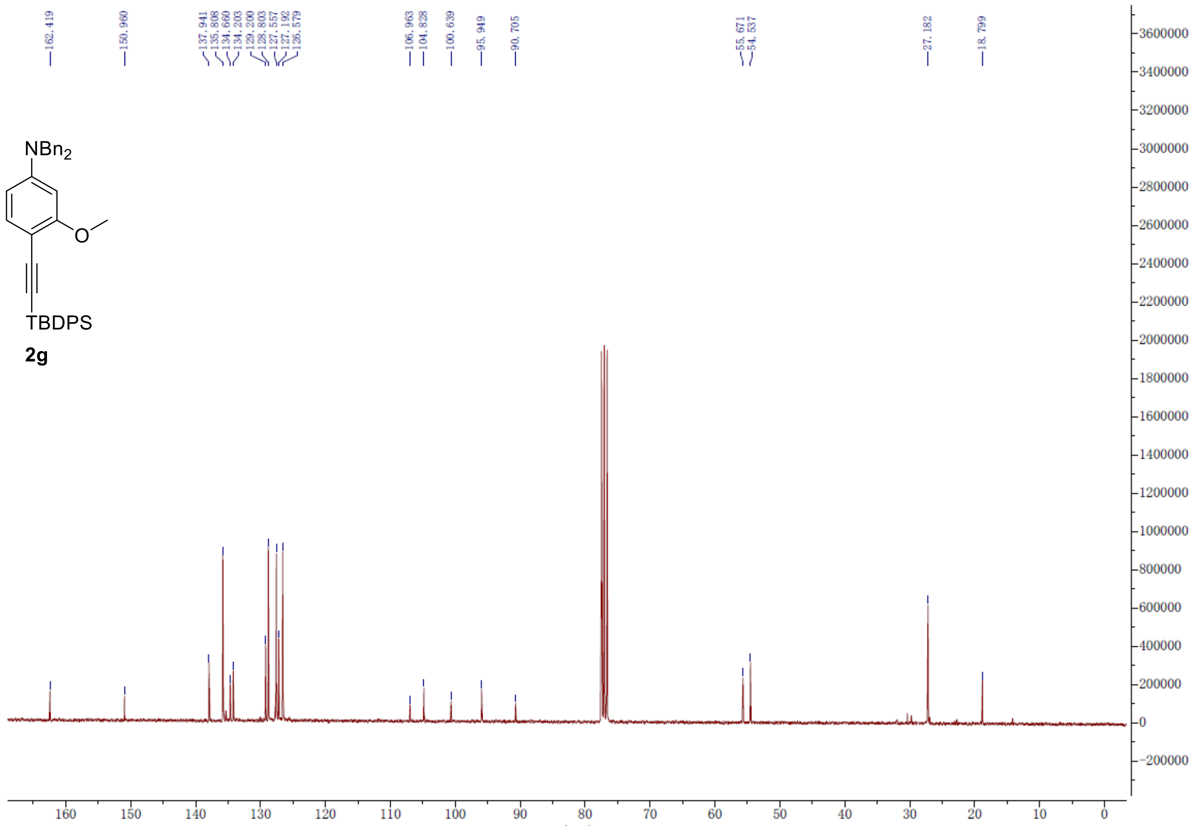
NBn2
O
TBDPS
2g

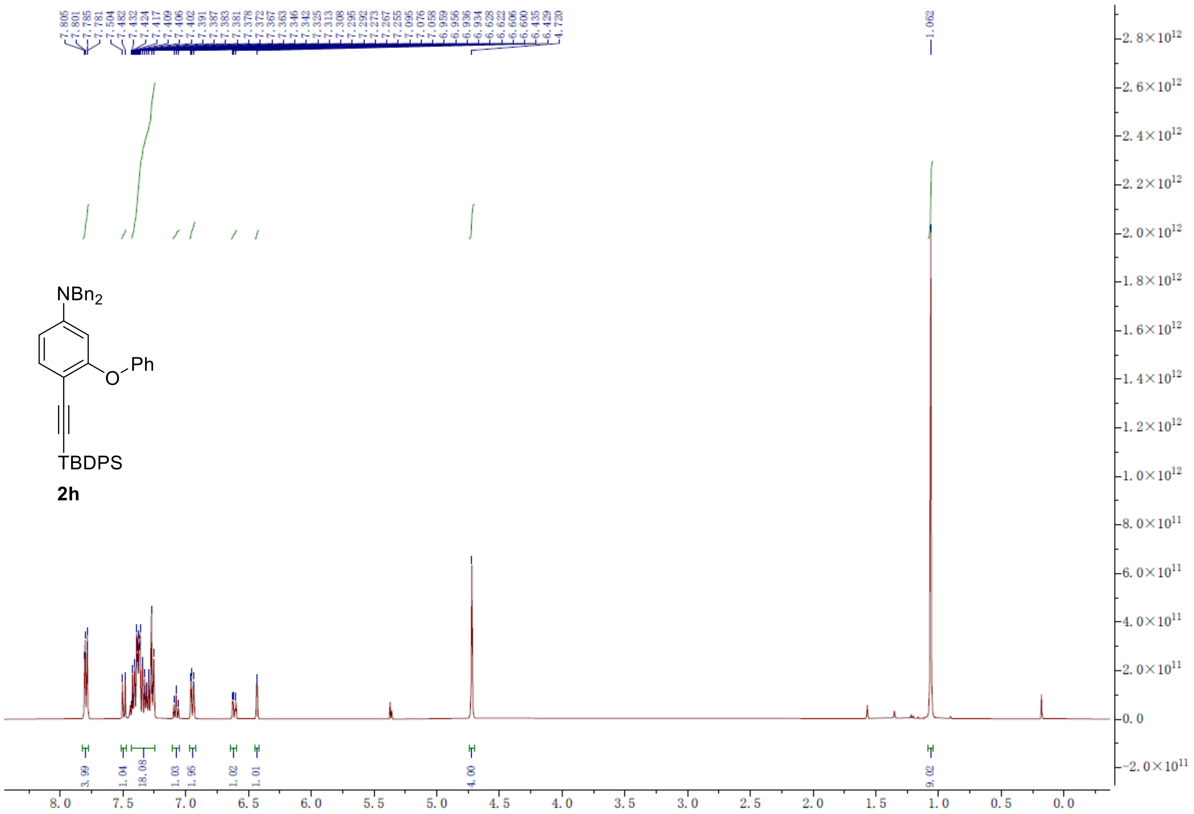

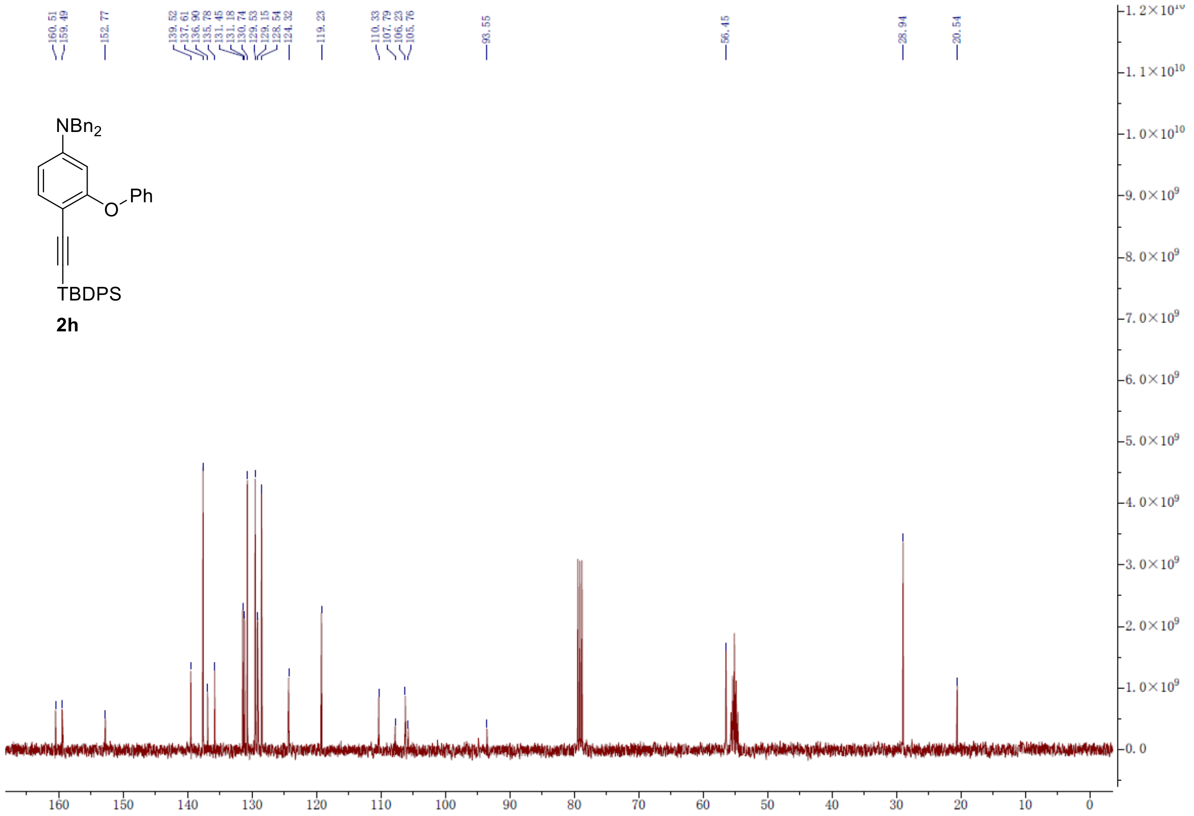

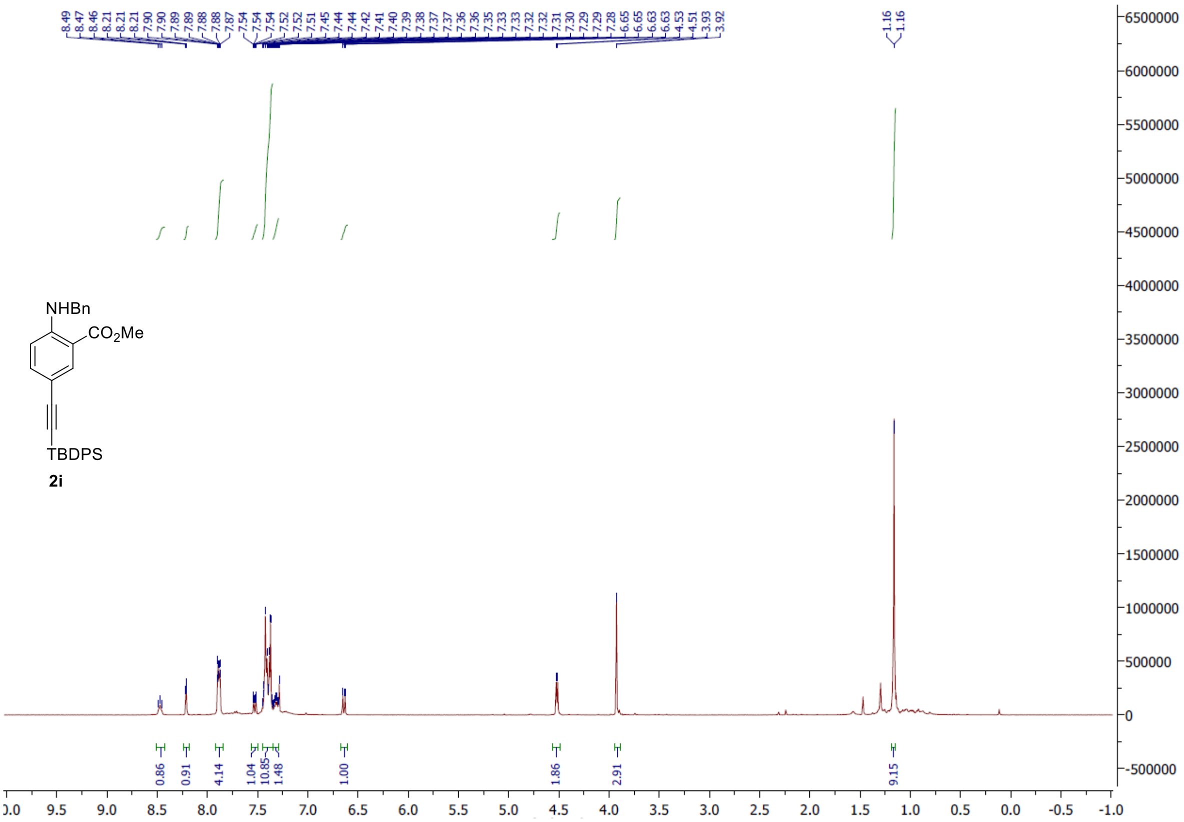

NHBn
CO2Me
TBDPS
2i

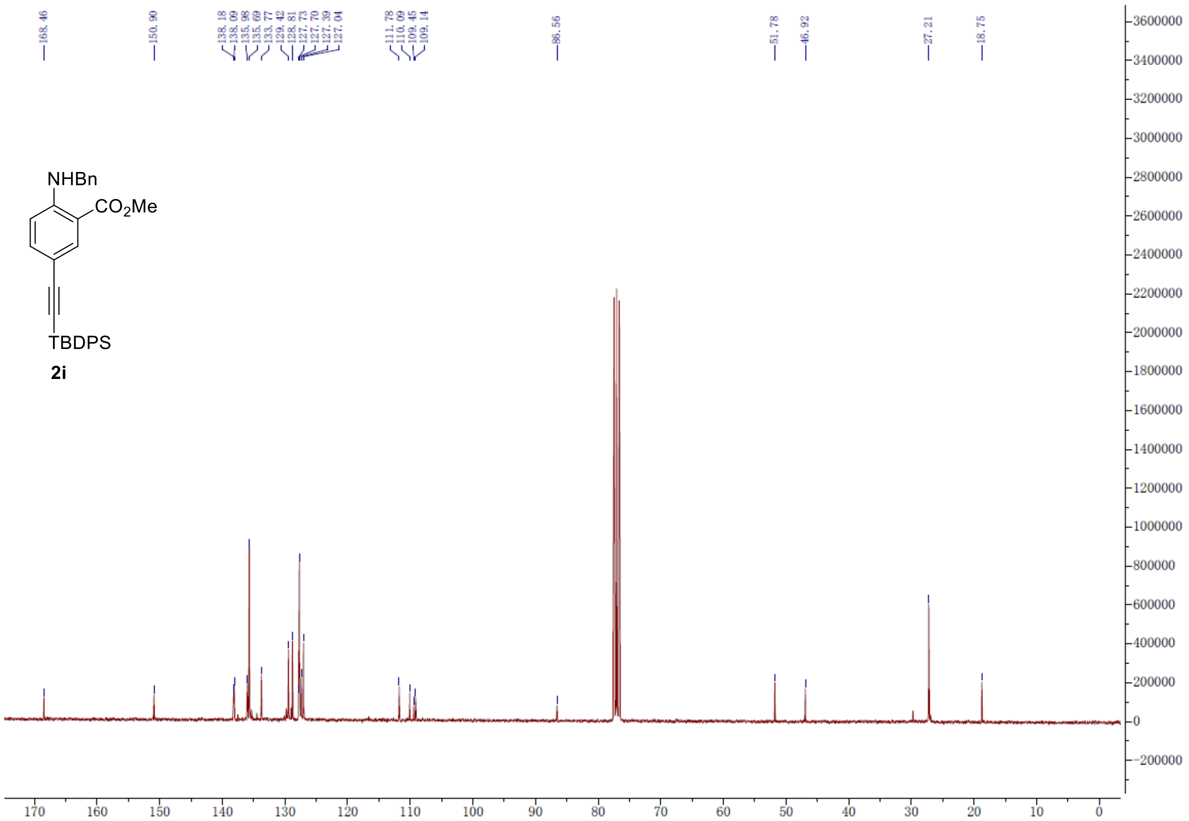

NHBn
CO2Me
TBDPS
2i

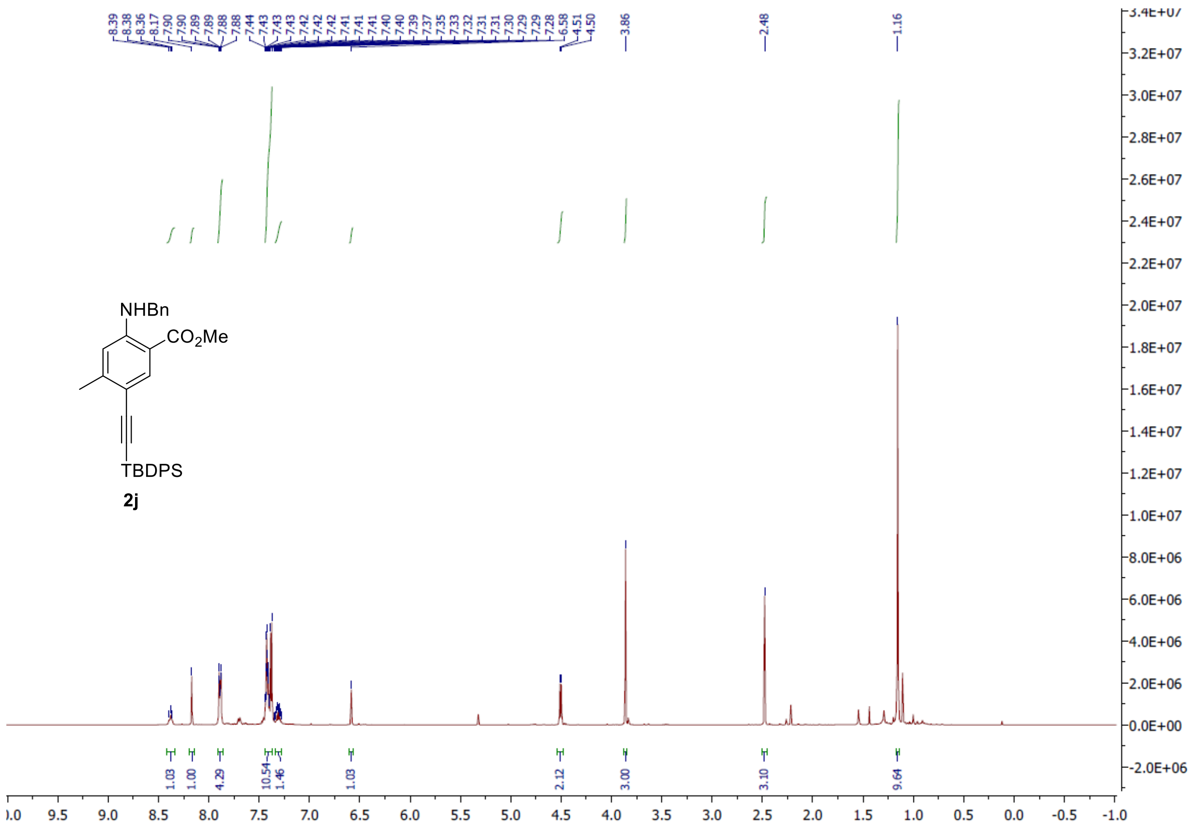

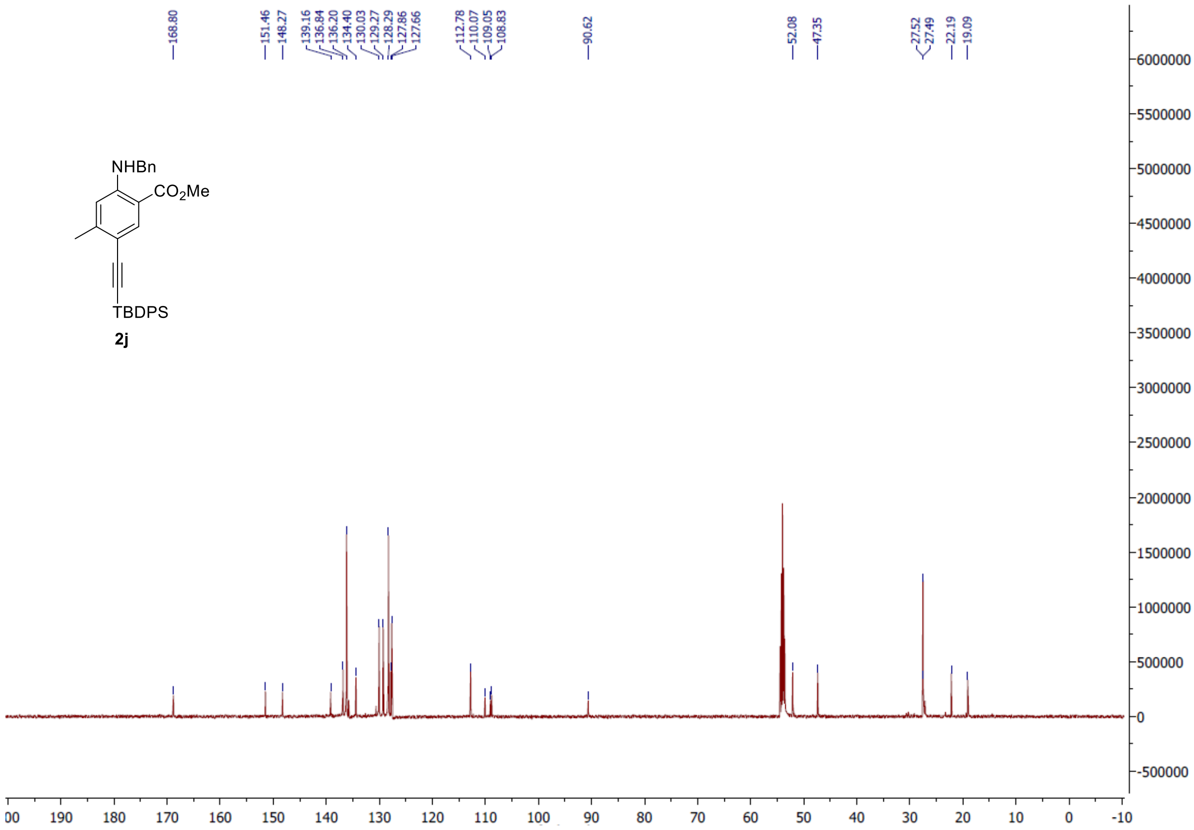

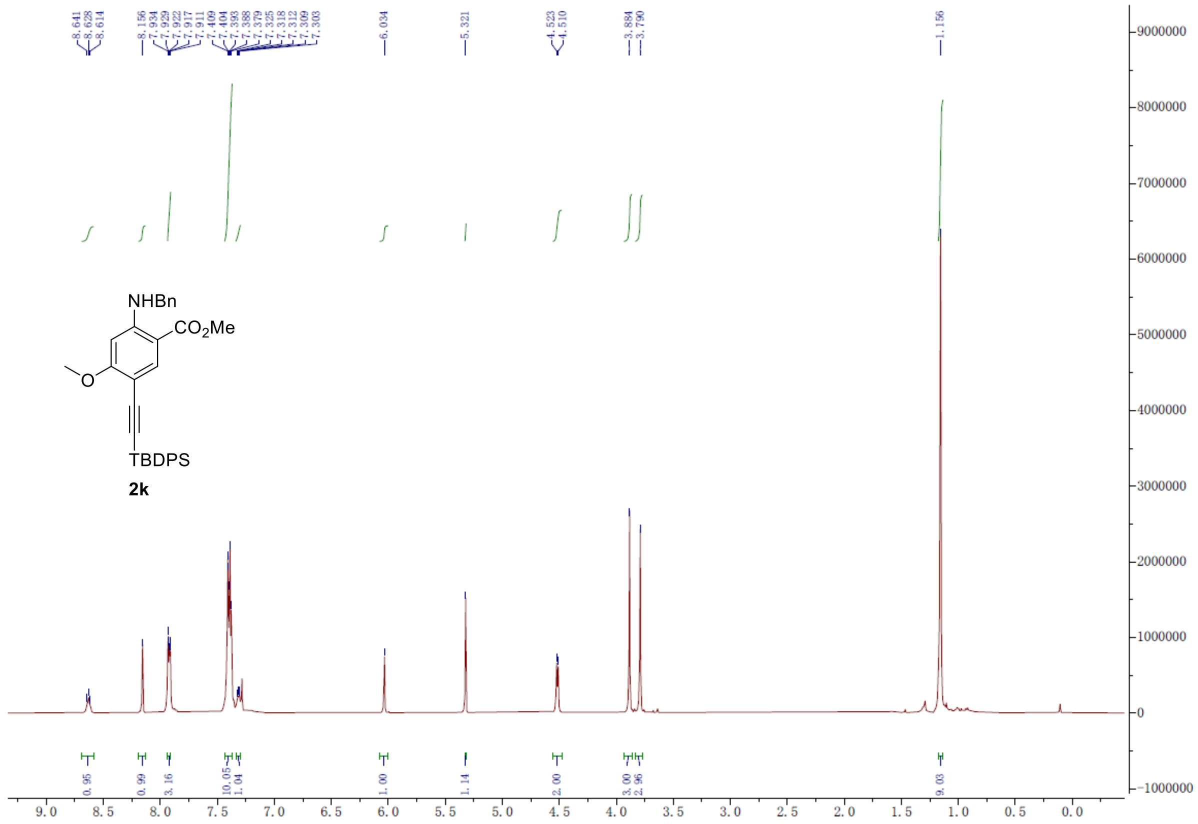

8.641
8.628
8.614
8.156
7.934
7.929
7.922
7.917
7.911
7.409
7.404
7.393
7.388
7.379
7.325
7.318
7.312
7.309
7.303
6.034
5.321
4.523
4.510
3.884
3.790
1.156
NHBn
CO2Me
O
TBDPS
2k
0.95
0.99
3.16
10.05
1.04
1.00
1.14
2.00
3.00
2.96
9.03
9.0
8.5
8.0
7.5
7.0
6.5
6.0
5.5
5.0
4.5
4.0
3.5
3.0
2.5
2.0
1.5
1.0
0.5
0.0
9000000
8000000
7000000
6000000
5000000
4000000
3000000
2000000
1000000
0
-1000000

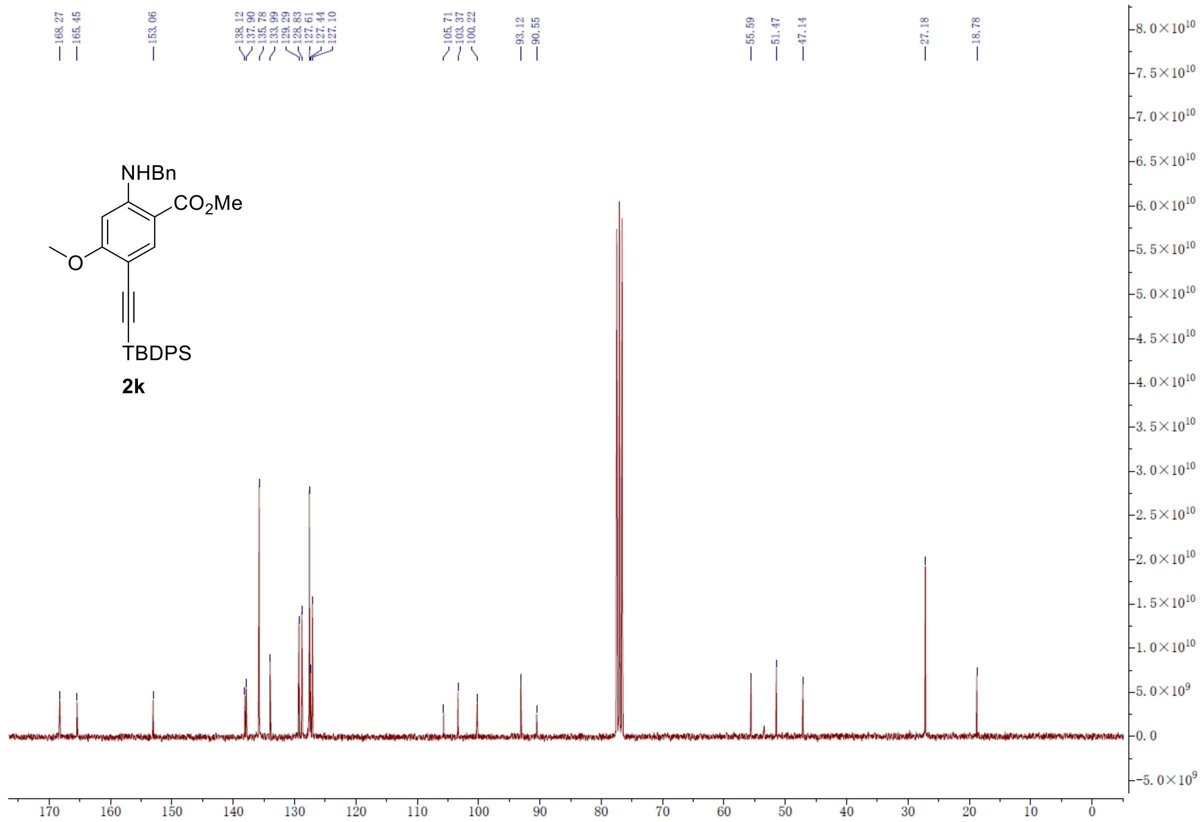

168.27
165.45
153.06
138.12
137.90
135.78
133.99
129.29
128.83
127.61
127.44
127.10
105.71
103.37
100.22
93.12
90.55
55.59
51.47
47.14
27.18
18.78
NHBn
CO2Me
O
TBDPS
2k
170
160
150
140
130
120
110
100
90
80
70
60
50
40
30
20
10
0

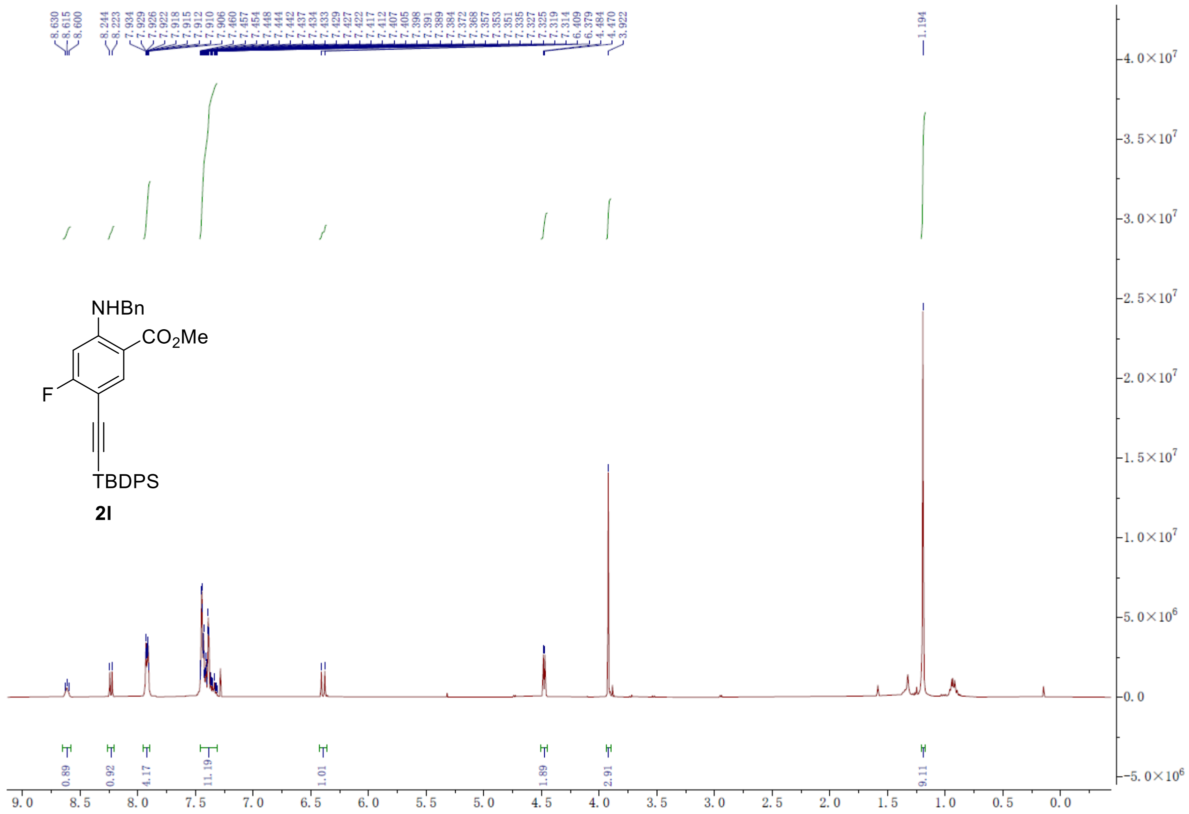
NHBn
CO_2Me
F
TBDPS
2l

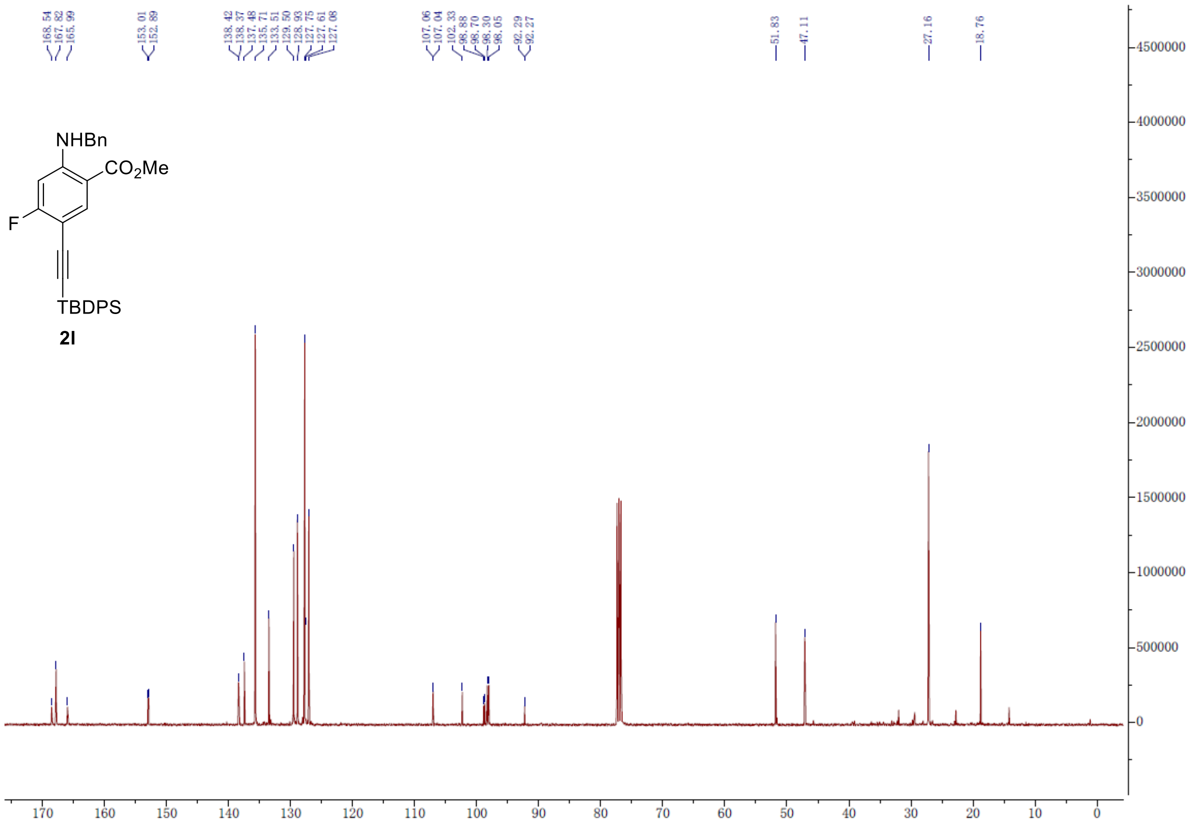
NHBn
CO_2Me
F
TBDPS
2l

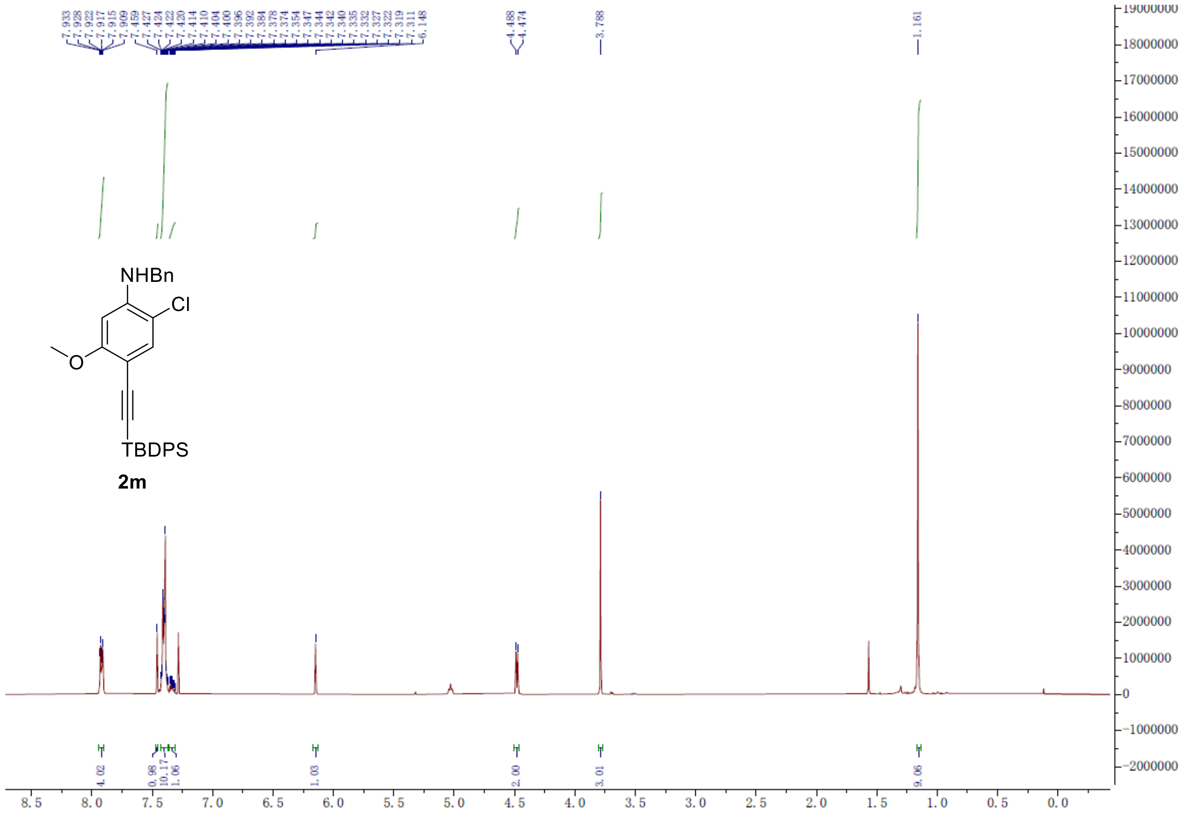
7.933 7.928 7.922 7.917 7.915 7.909 7.459 7.427 7.424 7.422 7.420 7.414 7.410 7.404 7.400 7.396 7.392 7.384 7.378 7.374 7.354 7.347 7.344 7.342 7.340 7.335 7.332 7.327 7.322 7.319 7.311 6.148
4.488 4.474
3.788
1.161
NHBn
Cl
O
TBDPS
2m
4.02
0.98
10.17
1.06
1.03
2.00
3.01
9.06
8.5 8.0 7.5 7.0 6.5 6.0 5.5 5.0 4.5 4.0 3.5 3.0 2.5 2.0 1.5 1.0 0.5 0.0

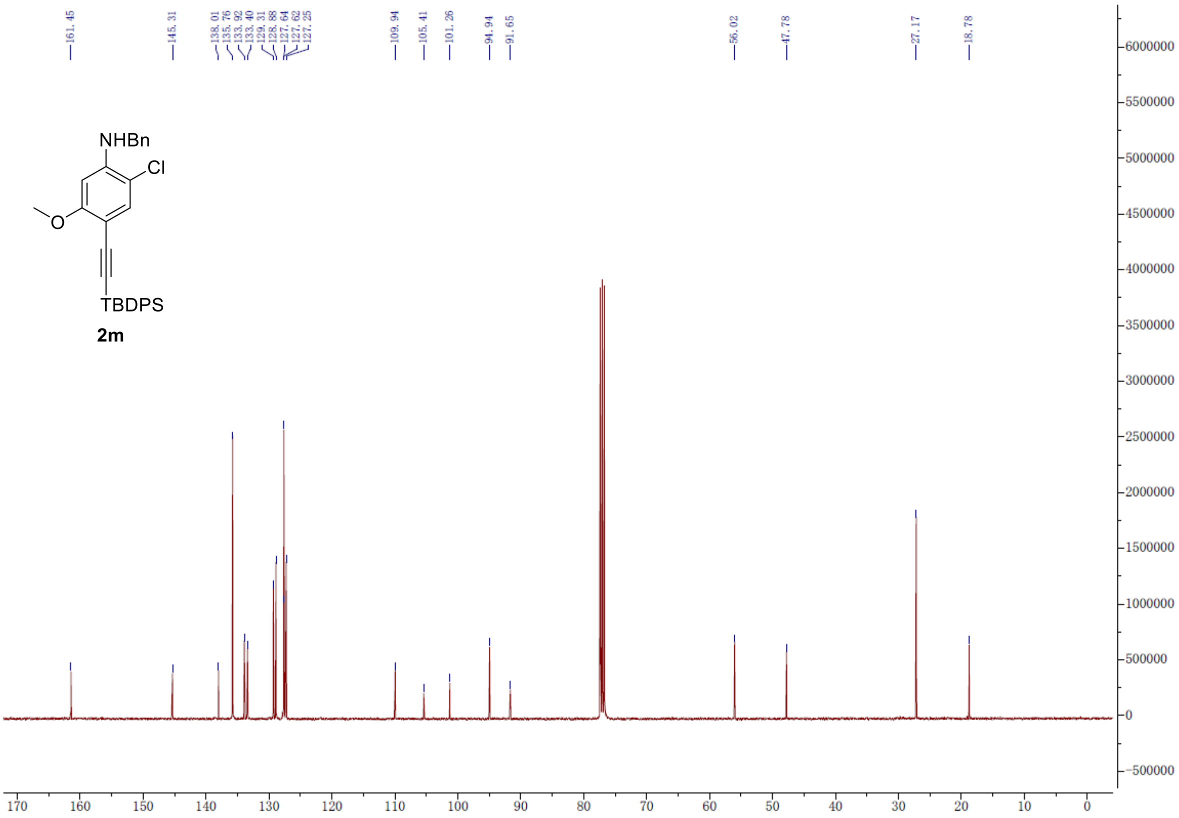
161.45
145.31
138.01 135.76 133.92 133.40 129.31 128.88 127.64 127.62 127.25
109.94
105.41
101.26
94.94
91.65
56.02
47.78
27.17
18.78
NHBn
Cl
O
TBDPS
2m
170 160 150 140 130 120 110 100 90 80 70 60 50 40 30 20 10 0

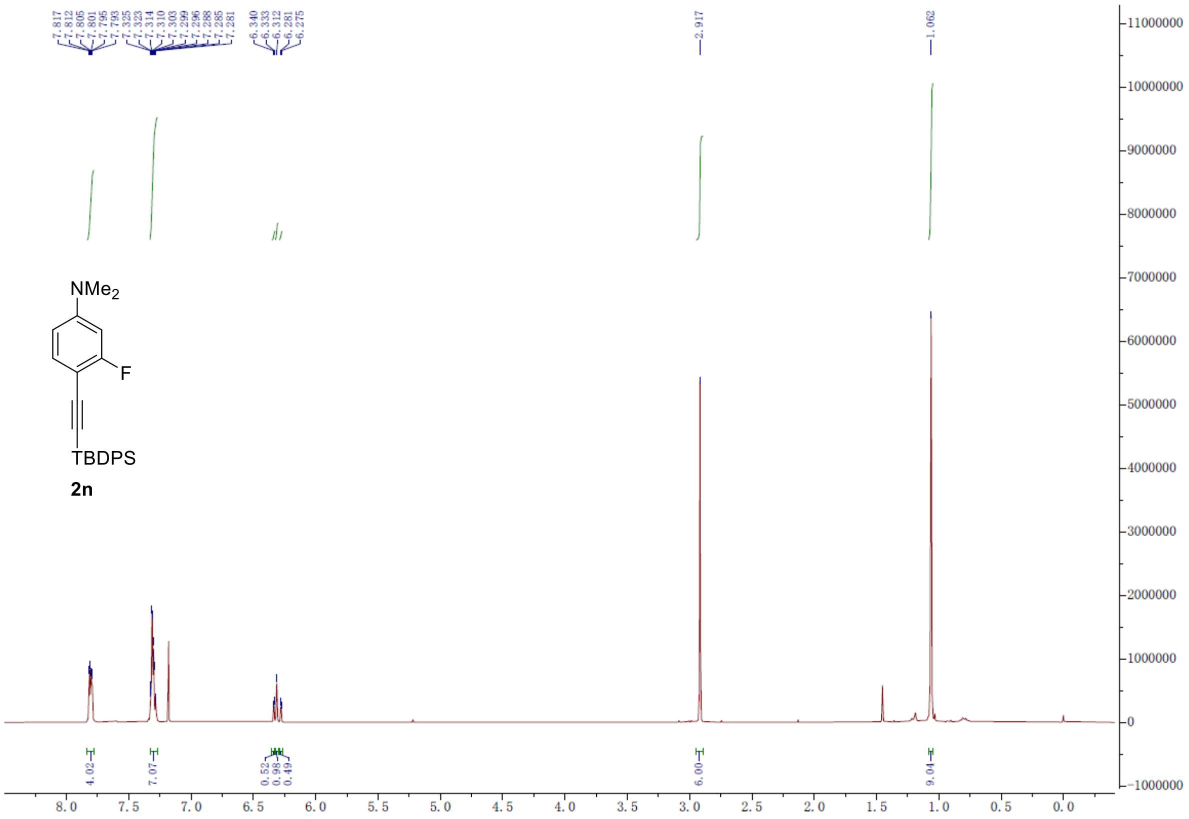

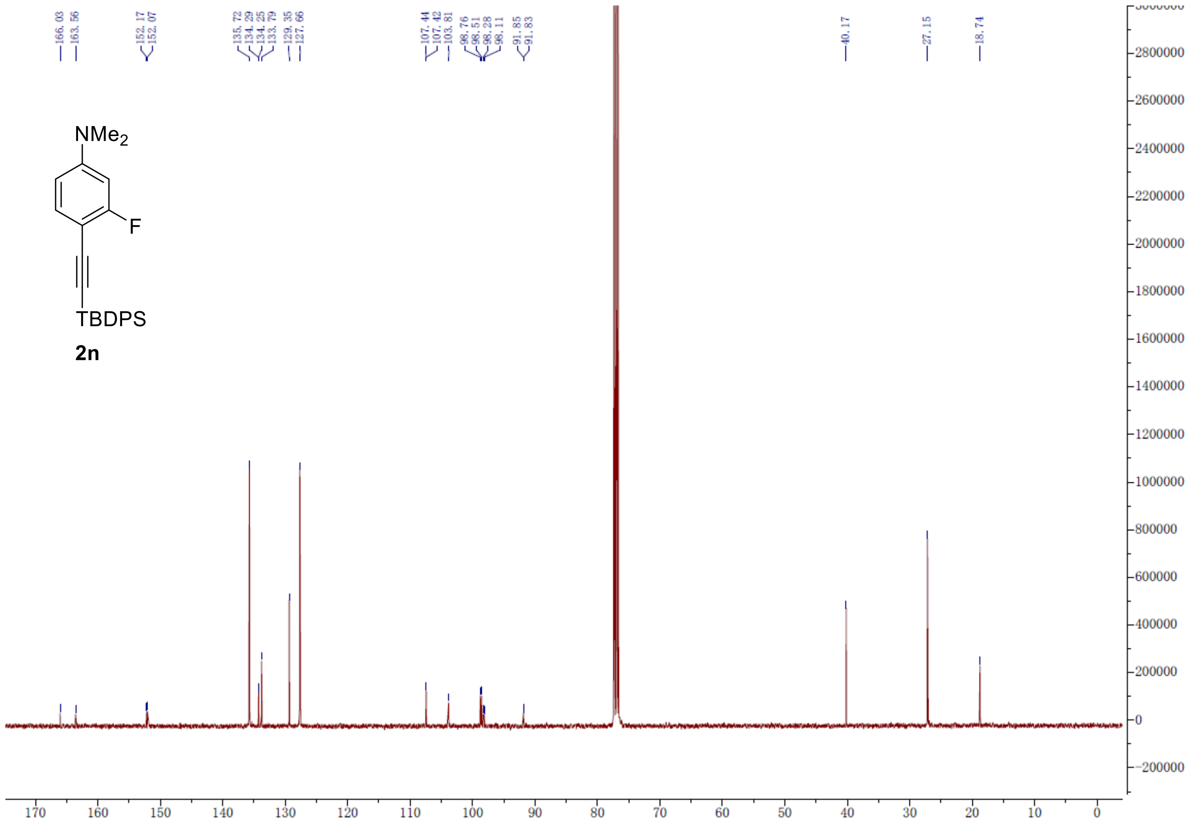

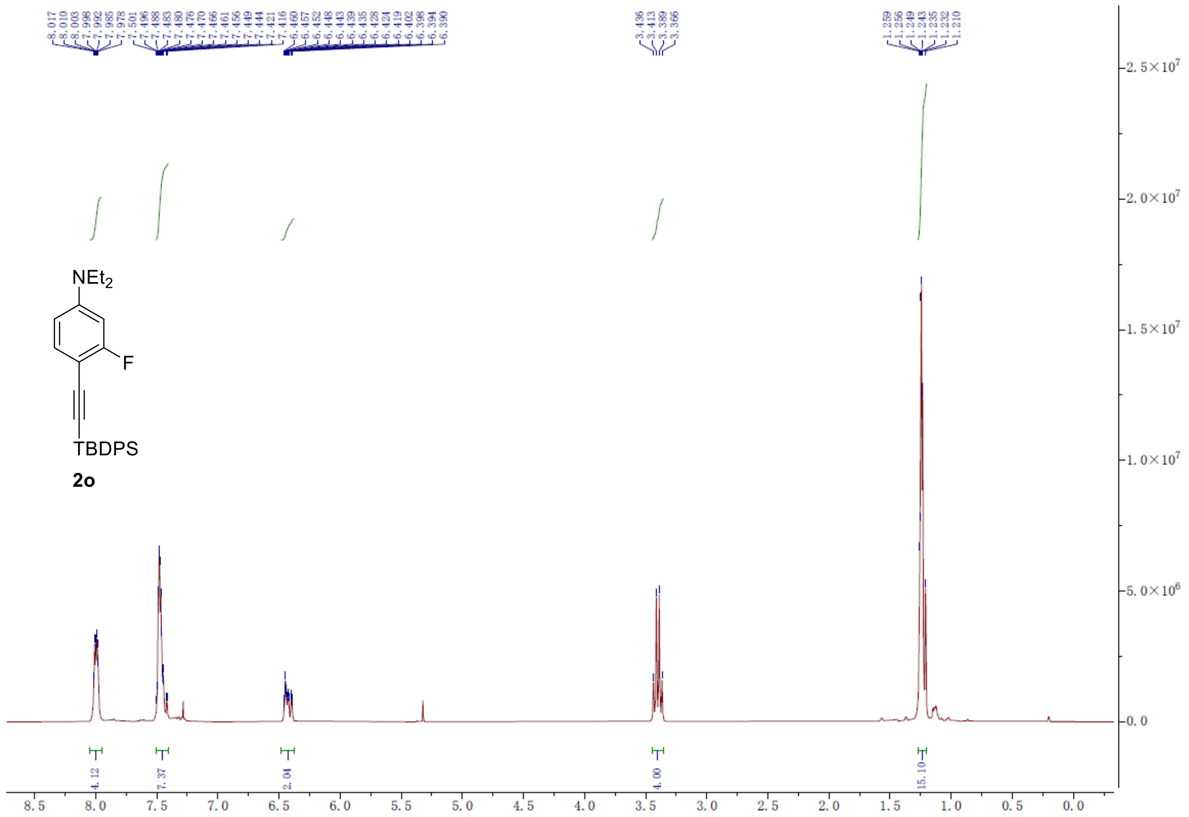
NEt2
F
TBDPS
2o

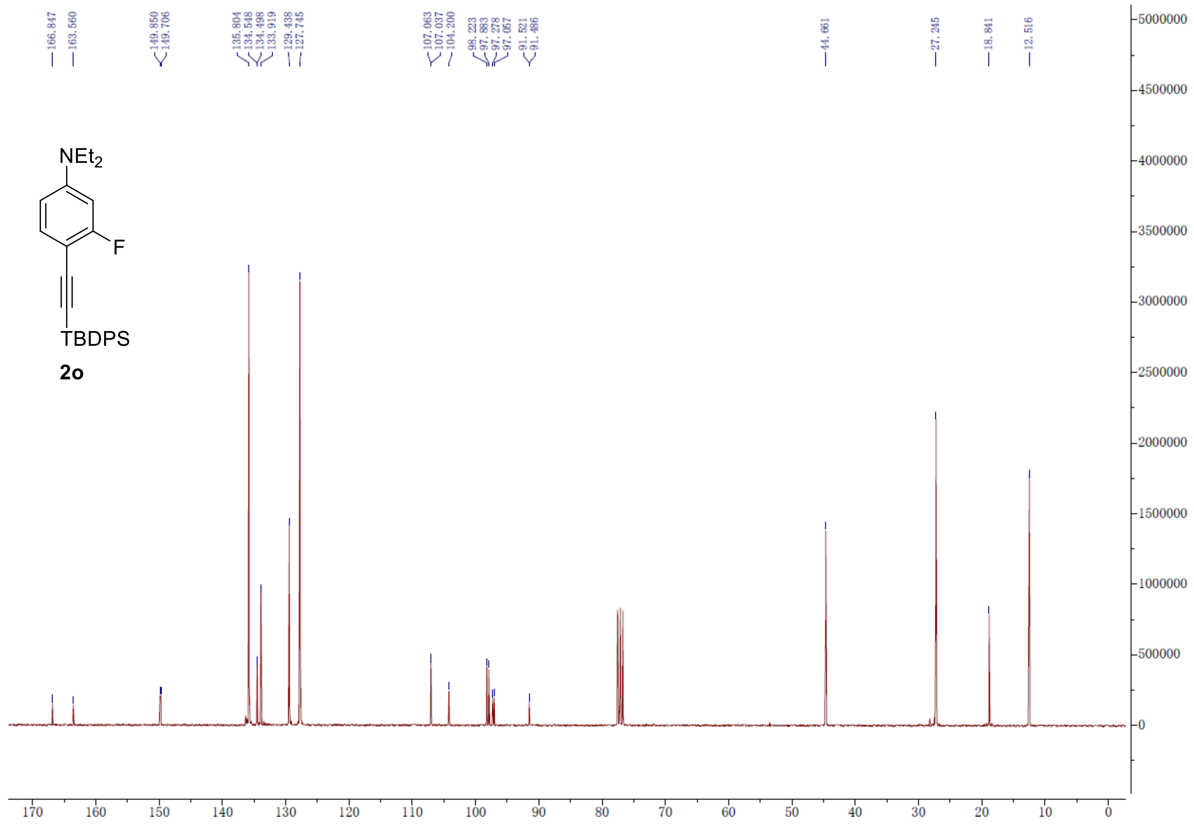
NEt2
F
TBDPS
2o

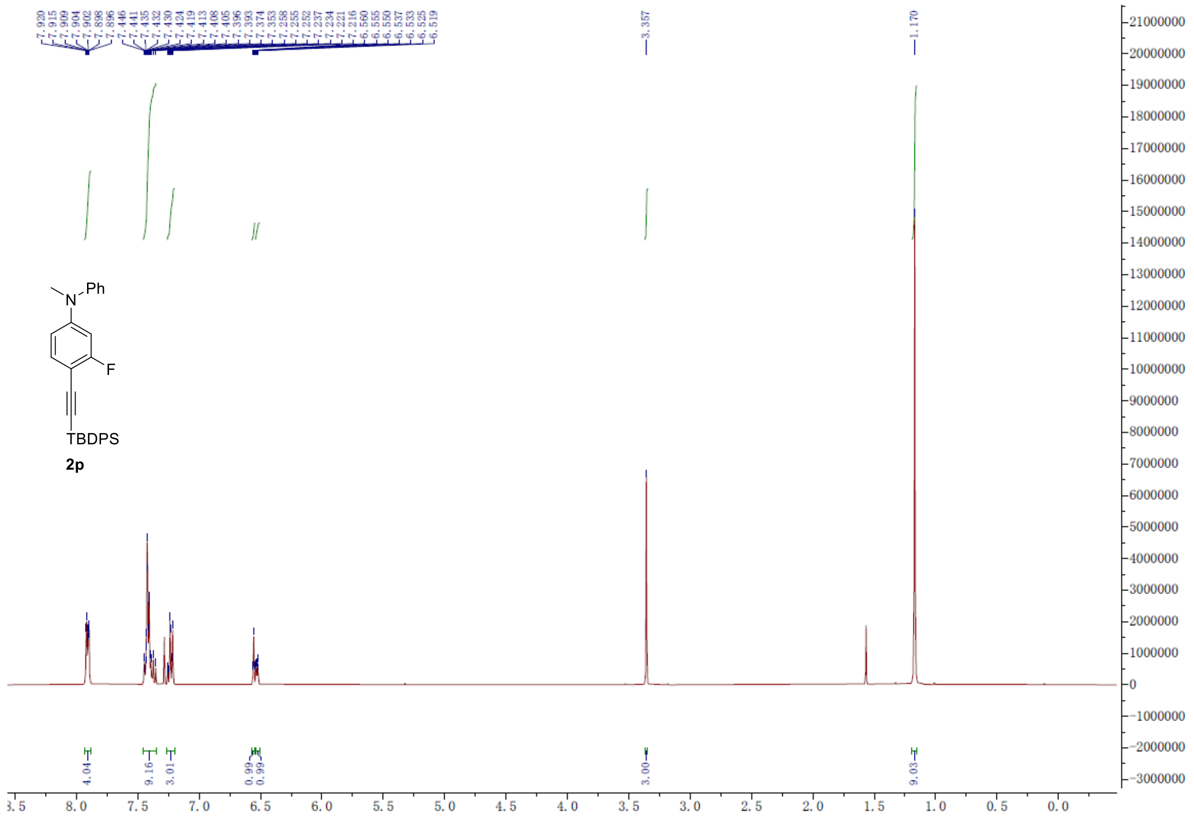

7.920 7.915 7.909 7.904 7.902 7.898 7.896 7.446 7.441 7.435 7.432 7.430 7.424 7.419 7.413 7.408 7.405 7.396 7.393 7.374 7.353 7.258 7.255 7.252 7.237 7.234 7.221 7.216 6.560 6.555 6.550 6.537 6.533 6.525 6.519
3.357
1.170
N
Ph
F
TBDPS
2p
4.04
9.16
3.01
0.99
0.99
3.00
9.03

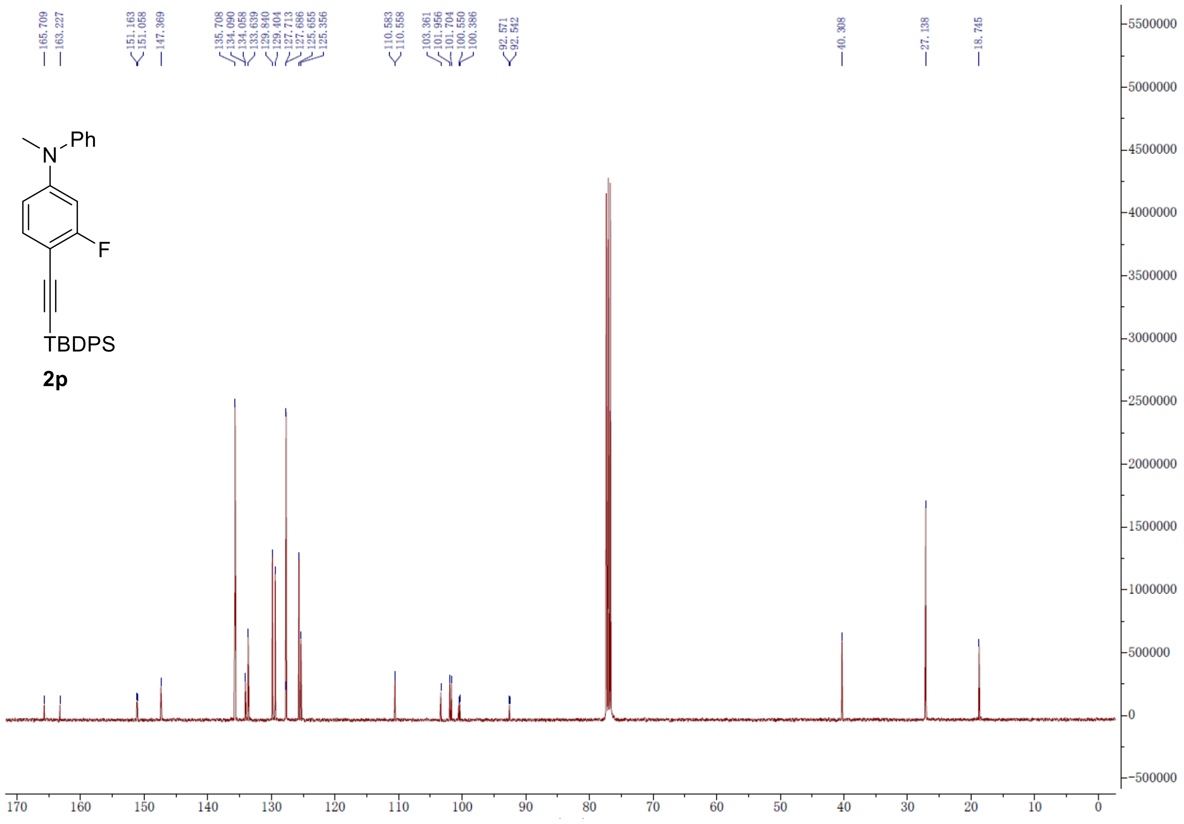

165.709 163.227
151.163 151.058 147.369
135.708 134.090 134.058 133.639 129.840 129.404 127.713 127.686 125.655 125.356
110.583 110.558
103.361 101.956 101.704 100.550 100.386
92.571 92.542
40.308
27.138
18.745
N
Ph
F
TBDPS
2p

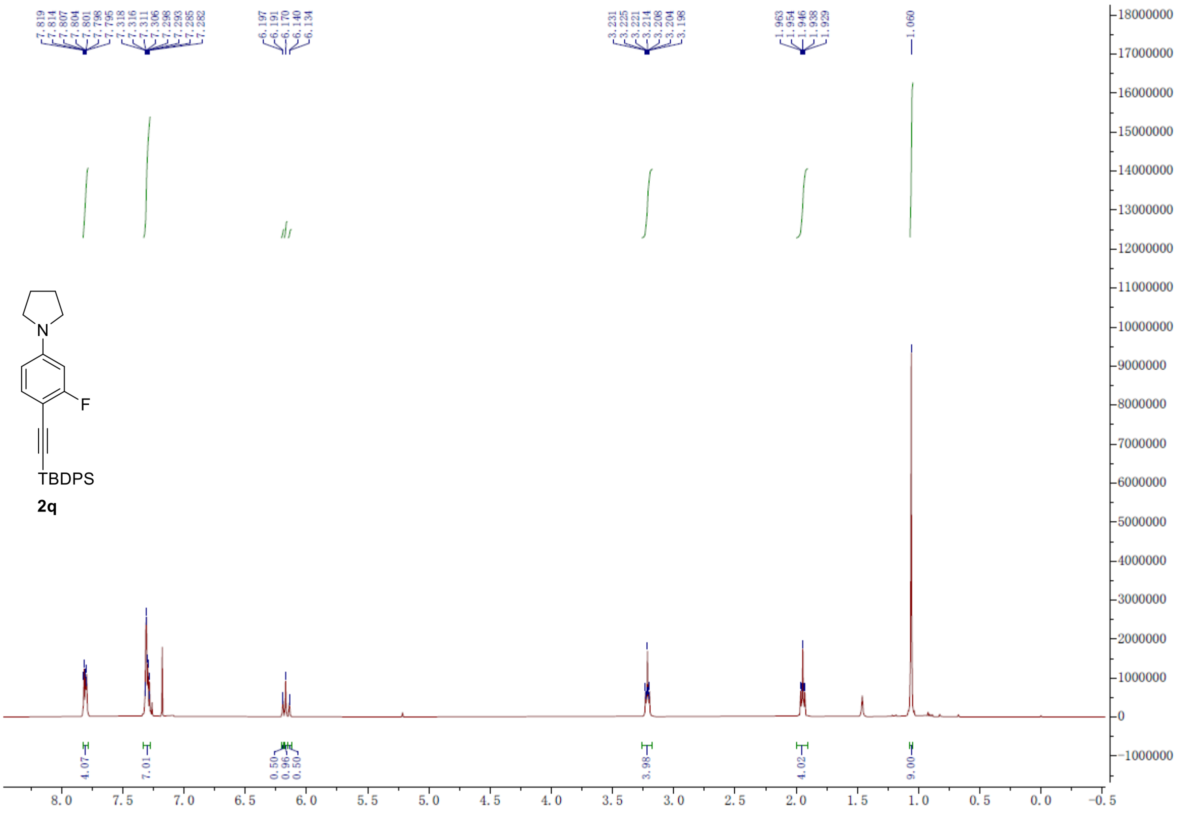
7.819 7.814 7.807 7.804 7.801 7.798 7.795 7.318 7.316 7.311 7.306 7.298 7.293 7.285 7.282
6.197 6.191 6.170 6.140 6.134
3.231 3.225 3.221 3.214 3.208 3.204 3.198
1.963 1.954 1.946 1.938 1.929
1.060
N
F
TBDPS
2q
4.07
7.01
0.50 0.96 0.50
3.98
4.02
9.00

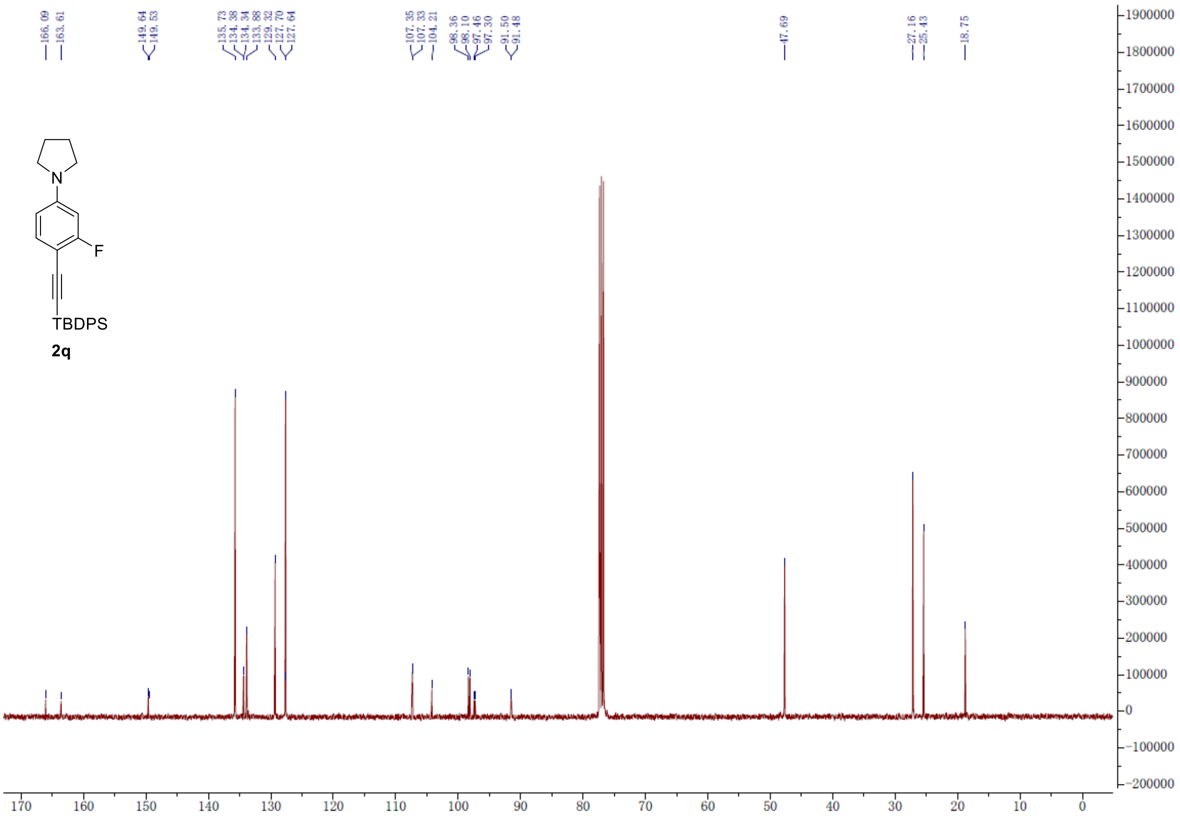
166.09 163.61
149.64 149.53
135.73 134.38 134.34 133.88 129.32 127.70 127.64
107.35 107.33 104.21 98.36 98.10 97.46 97.30 91.50 91.48
47.69
27.16 25.43
18.75
N
F
TBDPS
2q

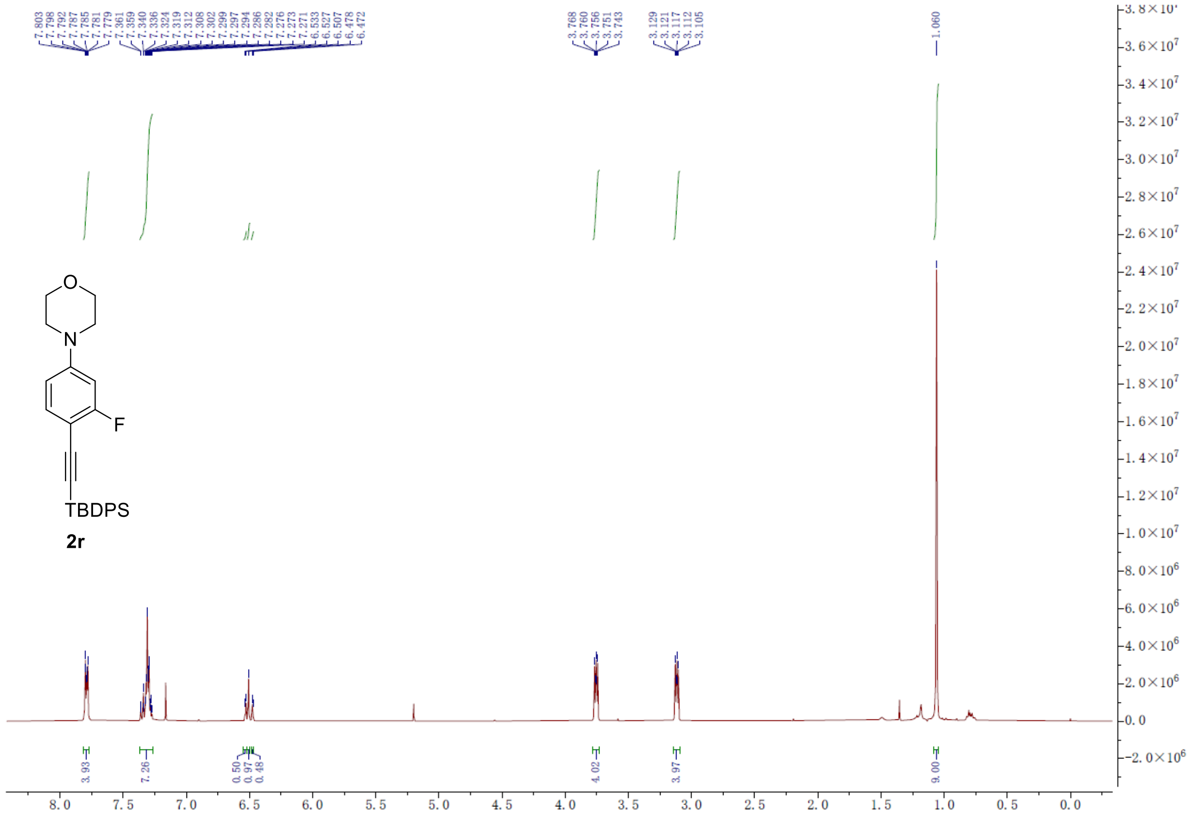

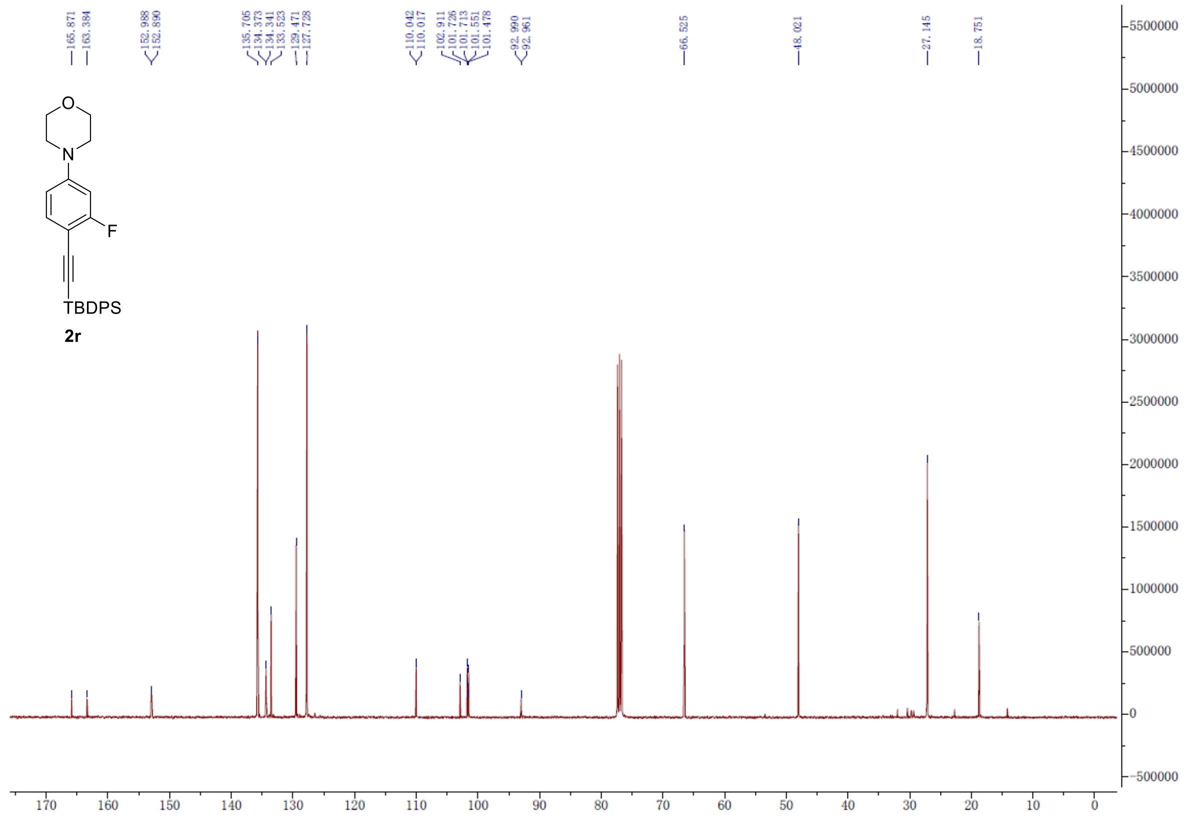

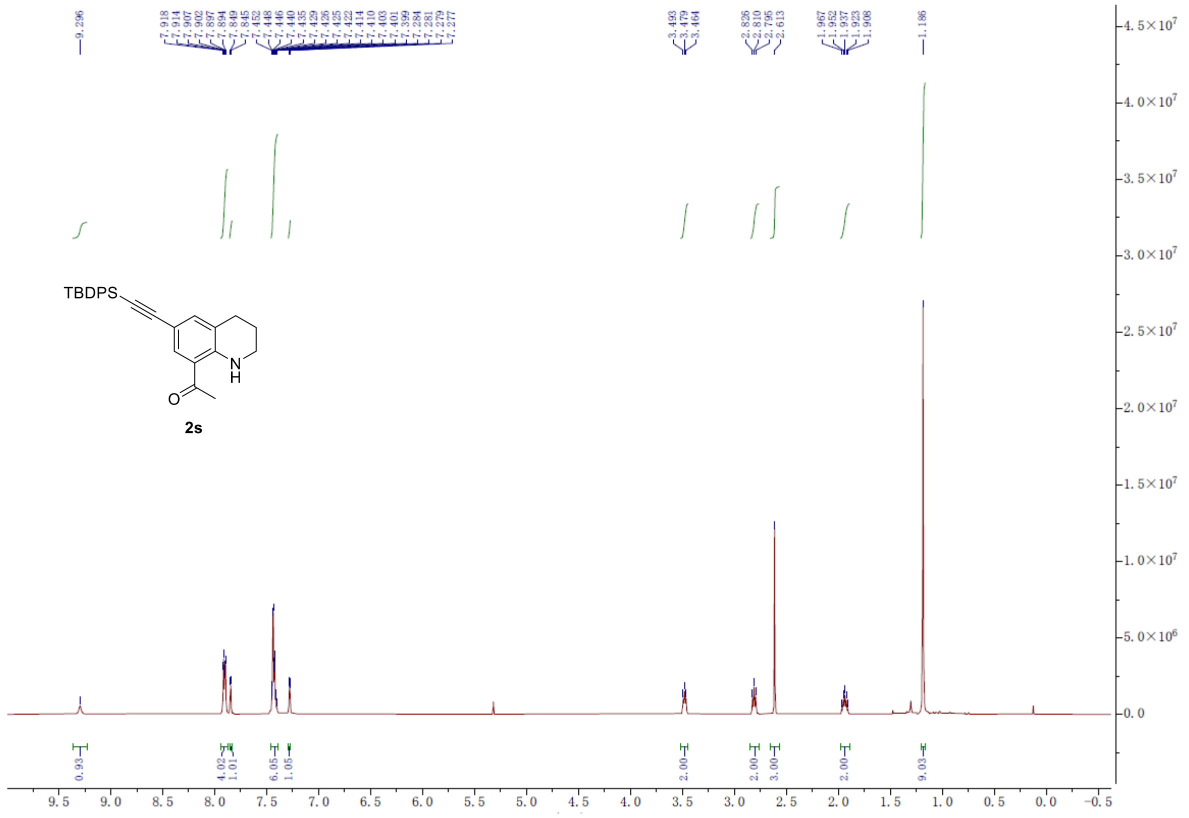
TBDPS
N
H
O
2s

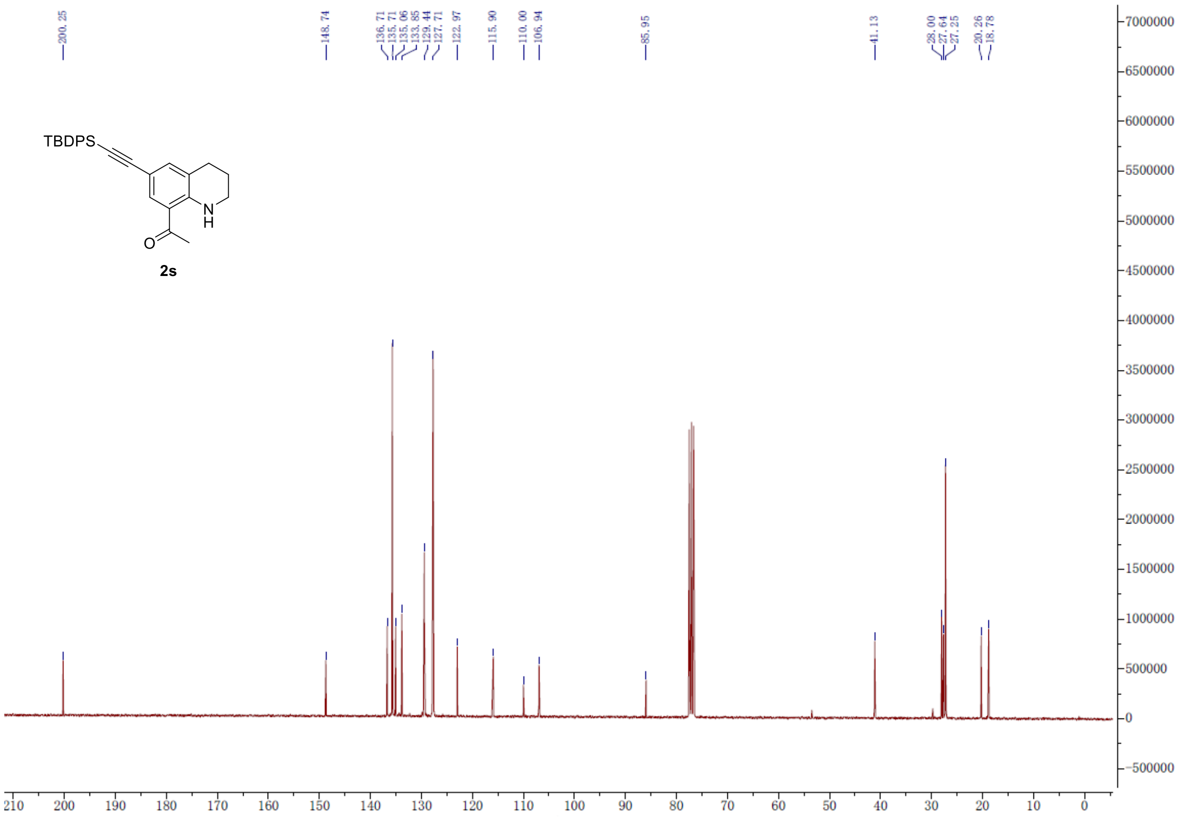
TBDPS
N
H
O
2s

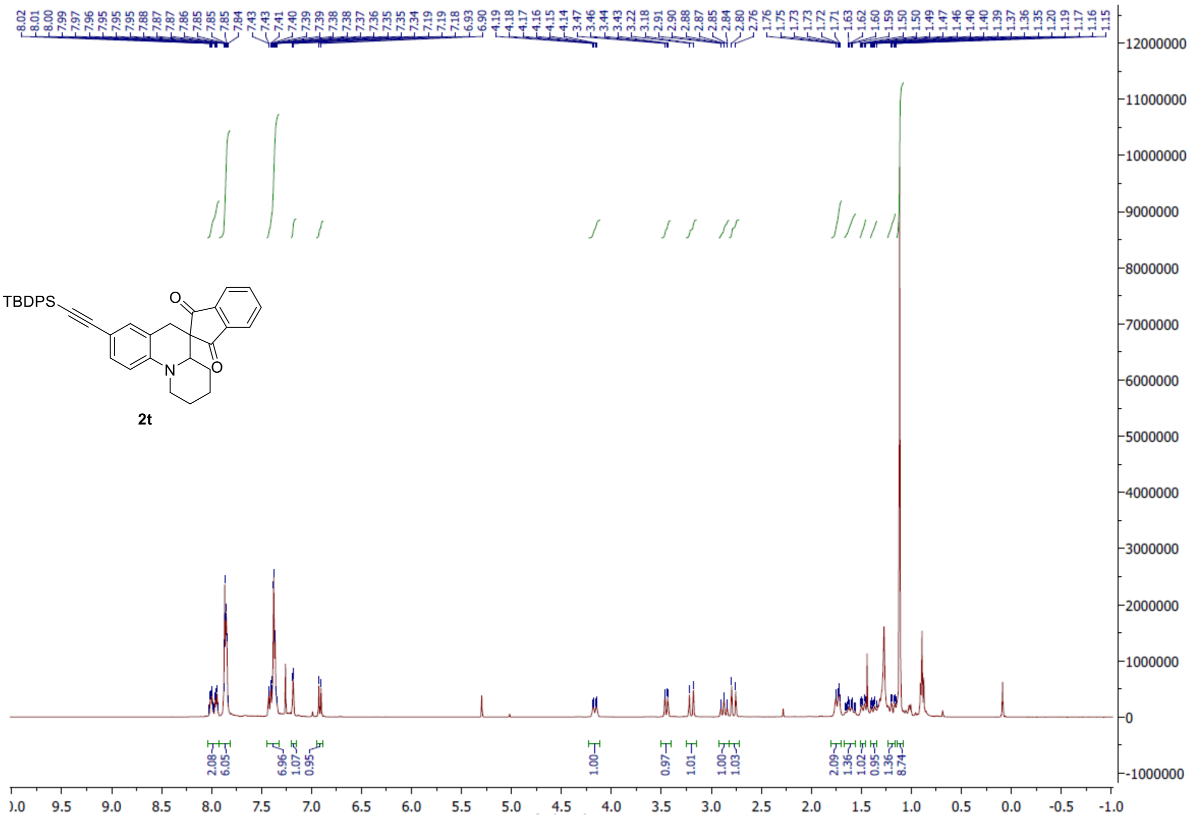
8.02 8.01 8.00 7.99 7.97 7.96 7.95 7.95 7.95 7.88 7.87 7.87 7.86 7.85 7.85 7.85 7.84 7.43 7.41 7.40 7.39 7.39 7.38 7.38 7.37 7.36 7.35 7.35 7.34 7.19 7.18 6.93 6.90 4.19 4.18 4.17 4.16 4.15 4.14 3.47 3.46 3.44 3.43 3.22 3.18 2.91 2.90 2.88 2.87 2.85 2.84 2.80 2.76 1.76 1.75 1.73 1.73 1.72 1.71 1.63 1.62 1.60 1.59 1.50 1.50 1.49 1.47 1.46 1.40 1.40 1.39 1.37 1.36 1.35 1.20 1.19 1.17 1.16 1.15
TBDPS
O
N
O
2t
2.08 6.05 6.96 1.07 0.95 1.00 0.97 1.01 1.00 1.03 2.09 1.36 1.02 0.95 1.36 8.74

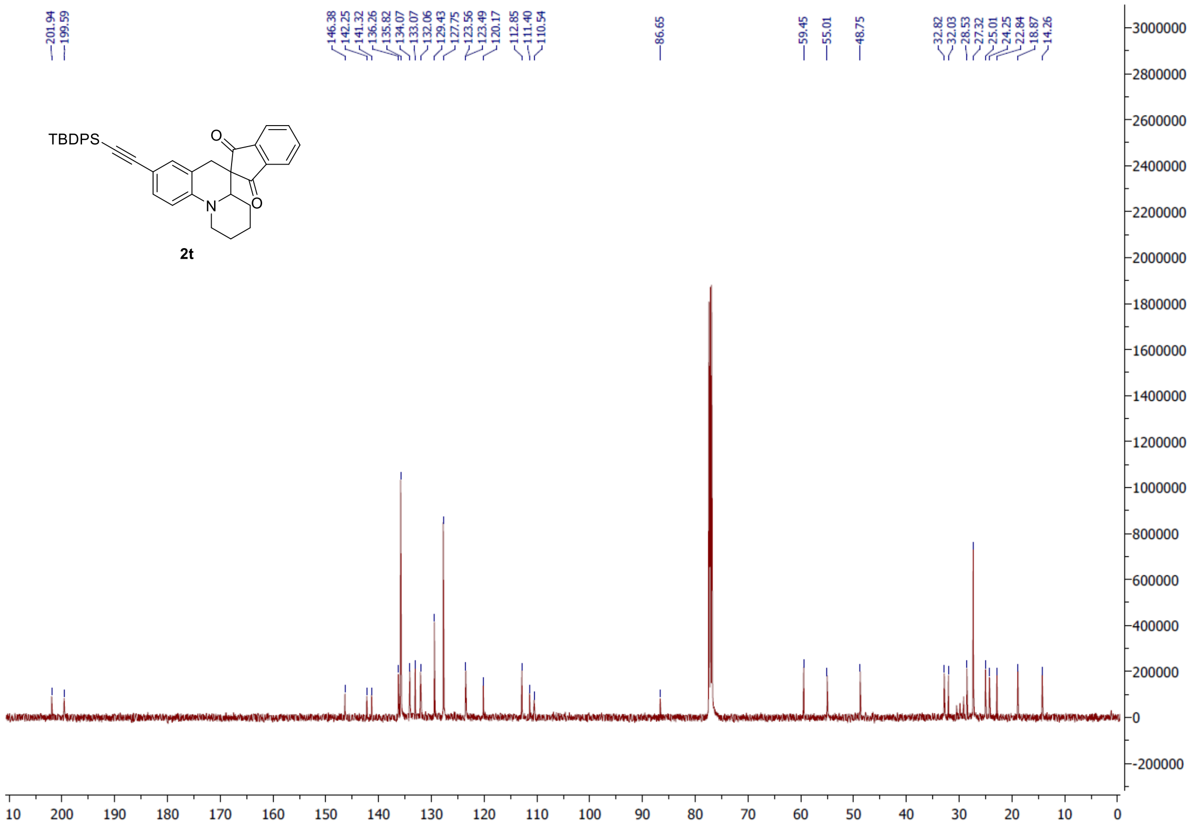
201.94 199.59 146.38 142.25 141.32 136.26 135.82 134.07 133.07 132.06 129.43 127.75 123.56 123.49 120.17 112.85 111.40 110.54 86.65 59.45 55.01 48.75 32.82 32.03 28.53 27.32 25.01 24.25 22.84 18.87 14.26
TBDPS
O
N
O
2t

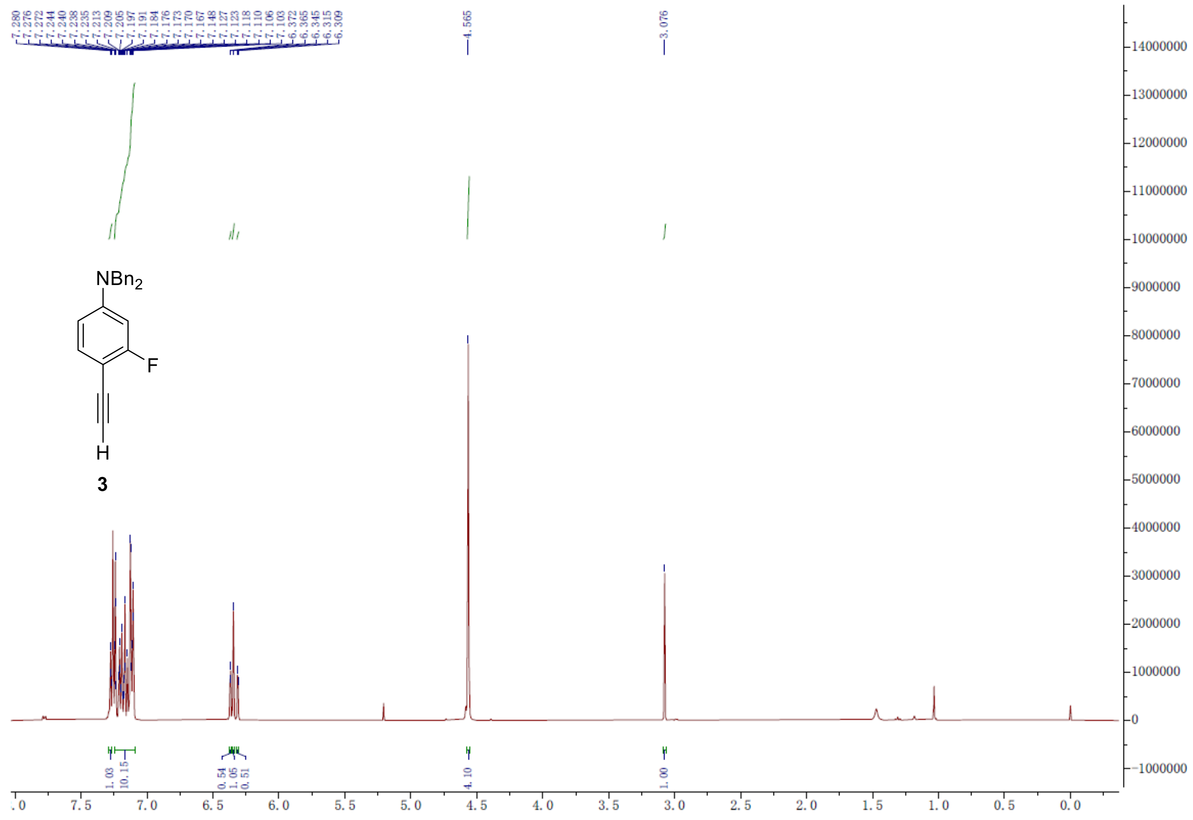

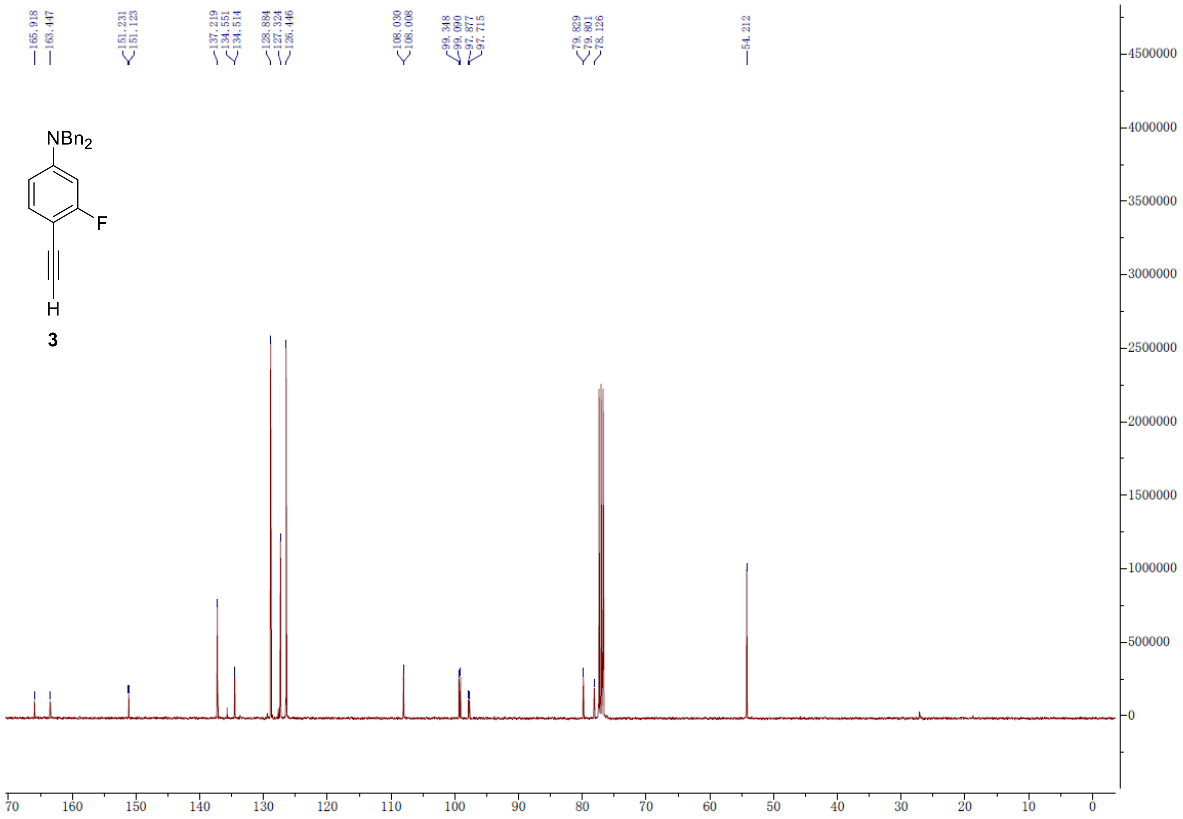

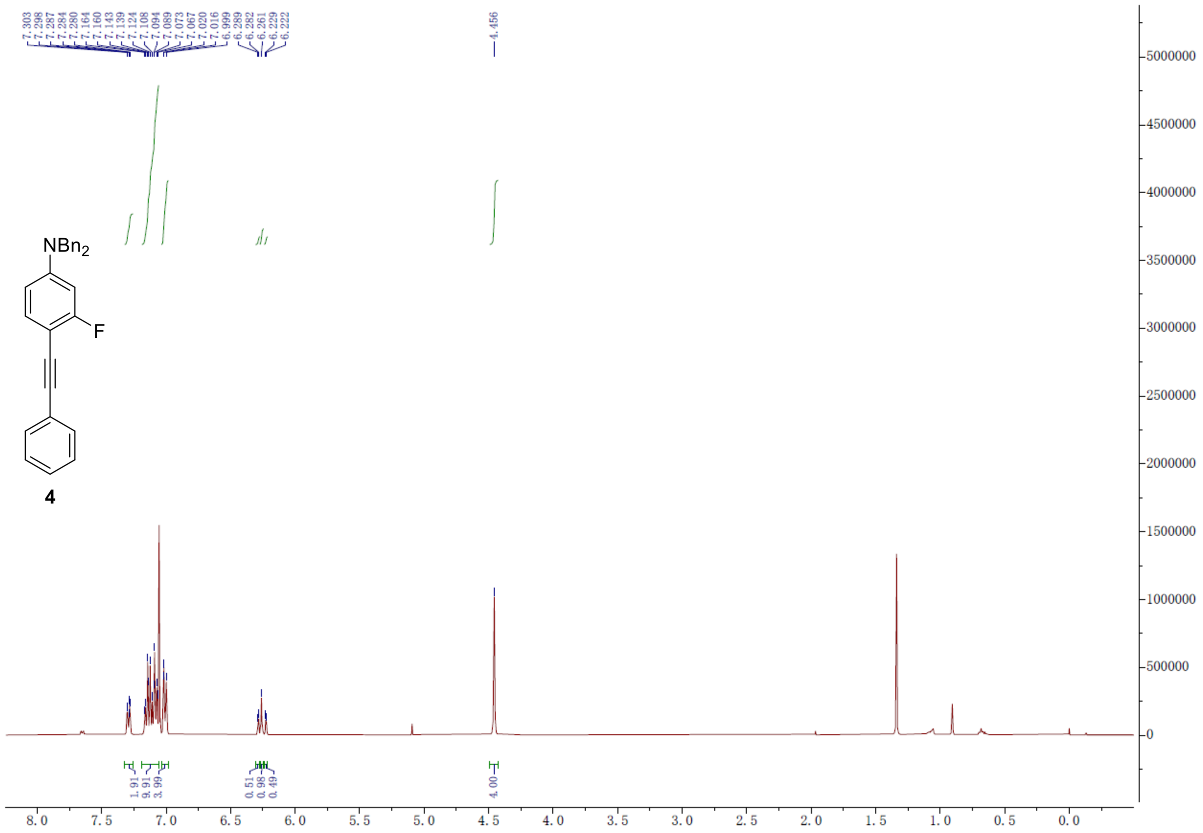
7.303
7.298
7.287
7.284
7.280
7.164
7.160
7.143
7.139
7.124
7.108
7.094
7.089
7.073
7.067
7.020
7.016
6.999
6.289
6.282
6.261
6.229
6.222
4.456
NBn2
F
4
1.91
9.91
3.99
0.51
0.98
0.49
4.00
8.0 7.5 7.0 6.5 6.0 5.5 5.0 4.5 4.0 3.5 3.0 2.5 2.0 1.5 1.0 0.5 0.0
5000000 4500000 4000000 3500000 3000000 2500000 2000000 1500000 1000000 500000 0

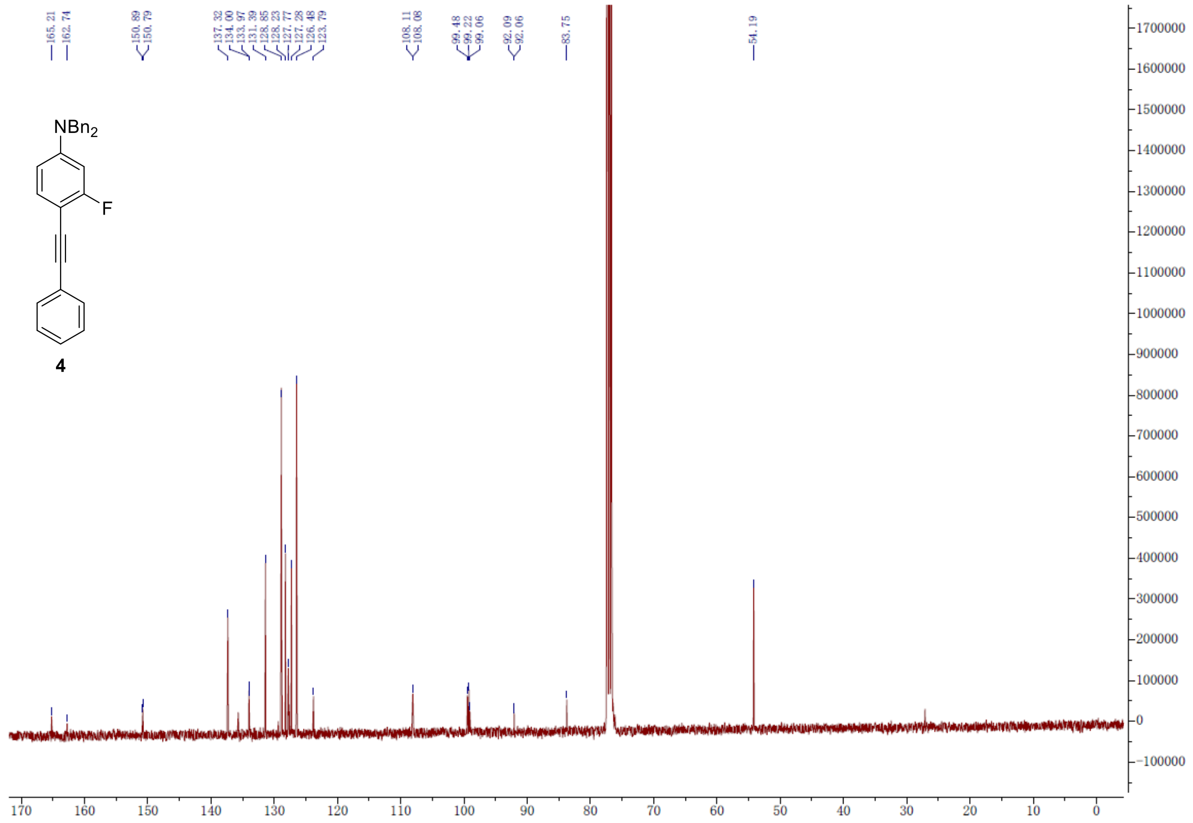
165.21
162.74
150.89
150.79
137.32
134.00
133.97
131.39
128.85
128.23
127.77
127.28
126.48
123.79
108.11
108.08
99.48
99.22
99.06
92.09
92.06
83.75
54.19
NBn2
F
4
170 160 150 140 130 120 110 100 90 80 70 60 50 40 30 20 10 0
1700000 1600000 1500000 1400000 1300000 1200000 1100000 1000000 900000 800000 700000 600000 500000 400000 300000 200000 100000 0 -100000

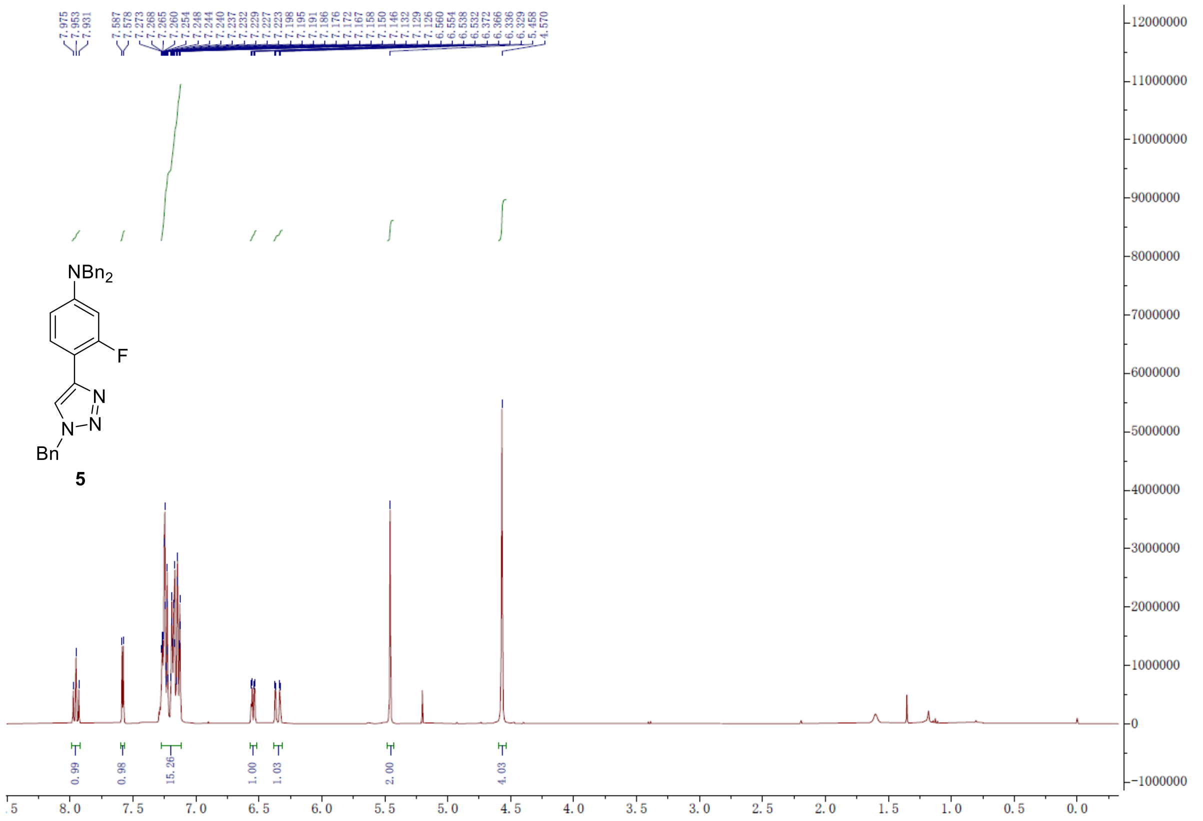

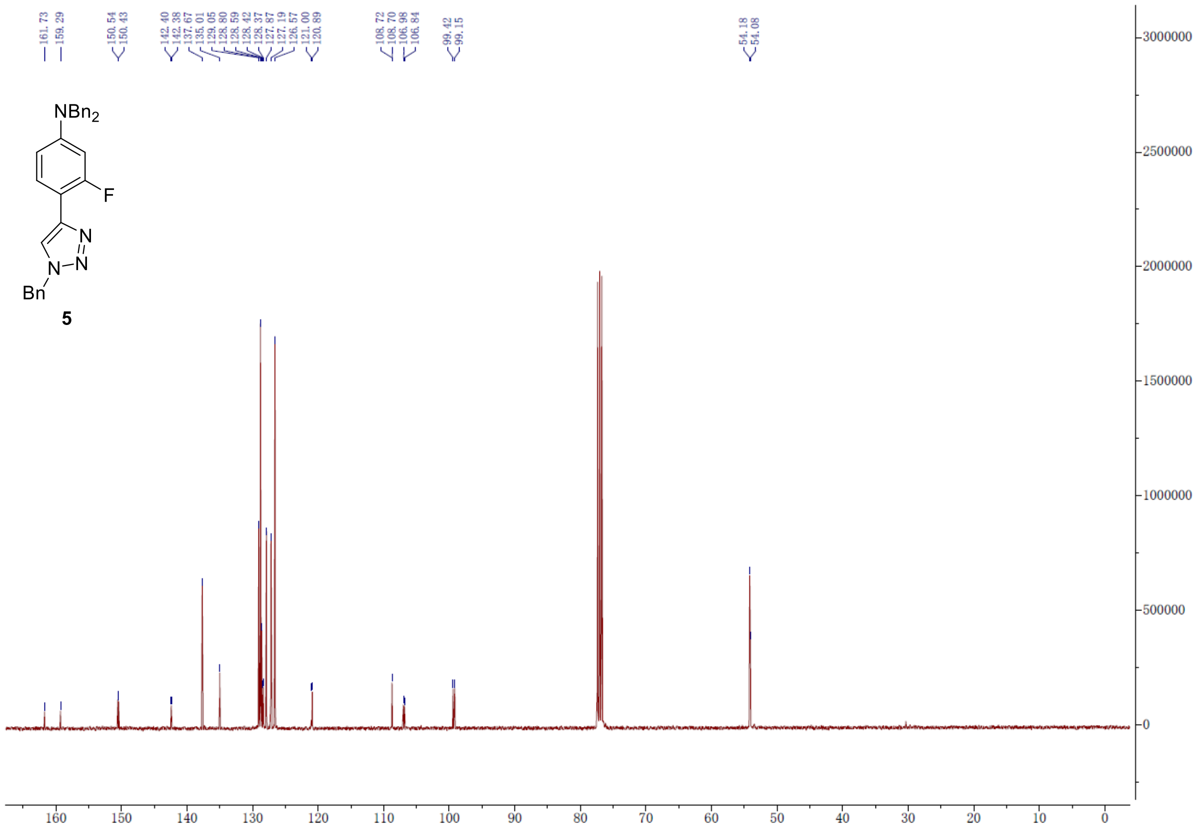

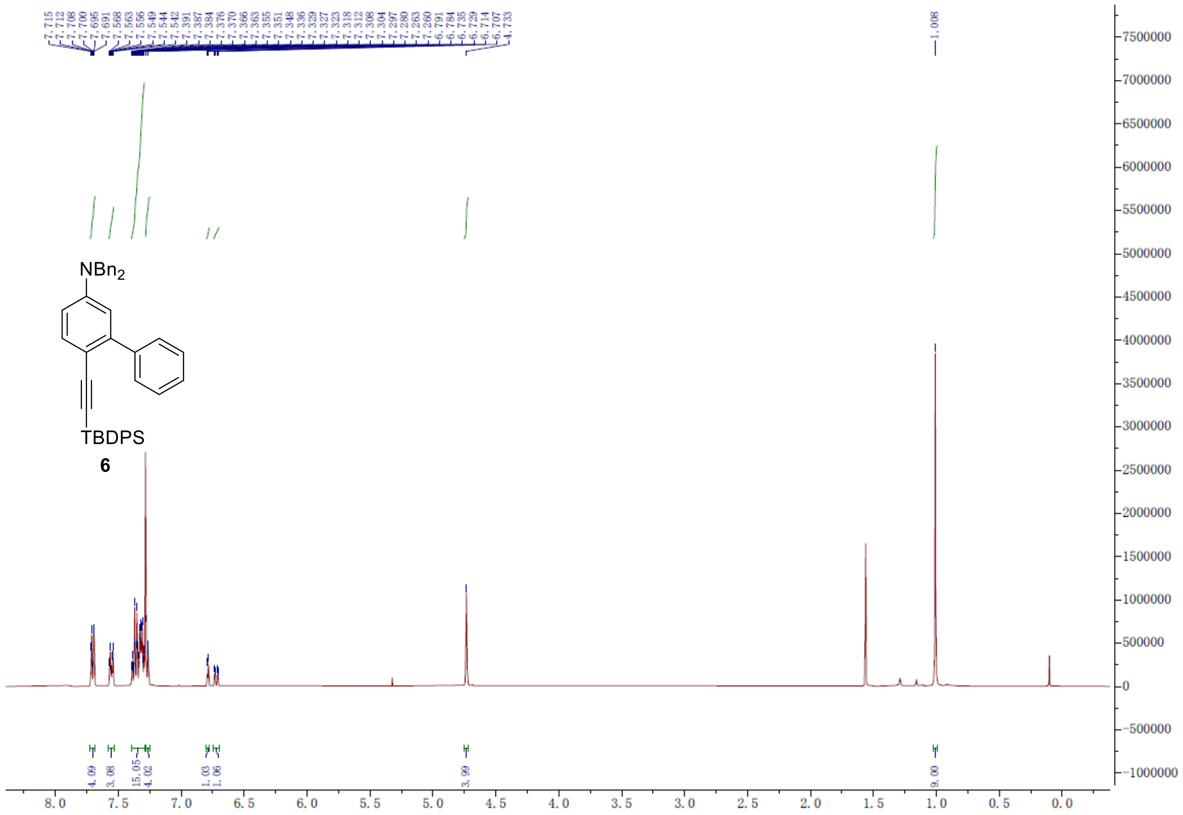
NBn2
TBDPS
6

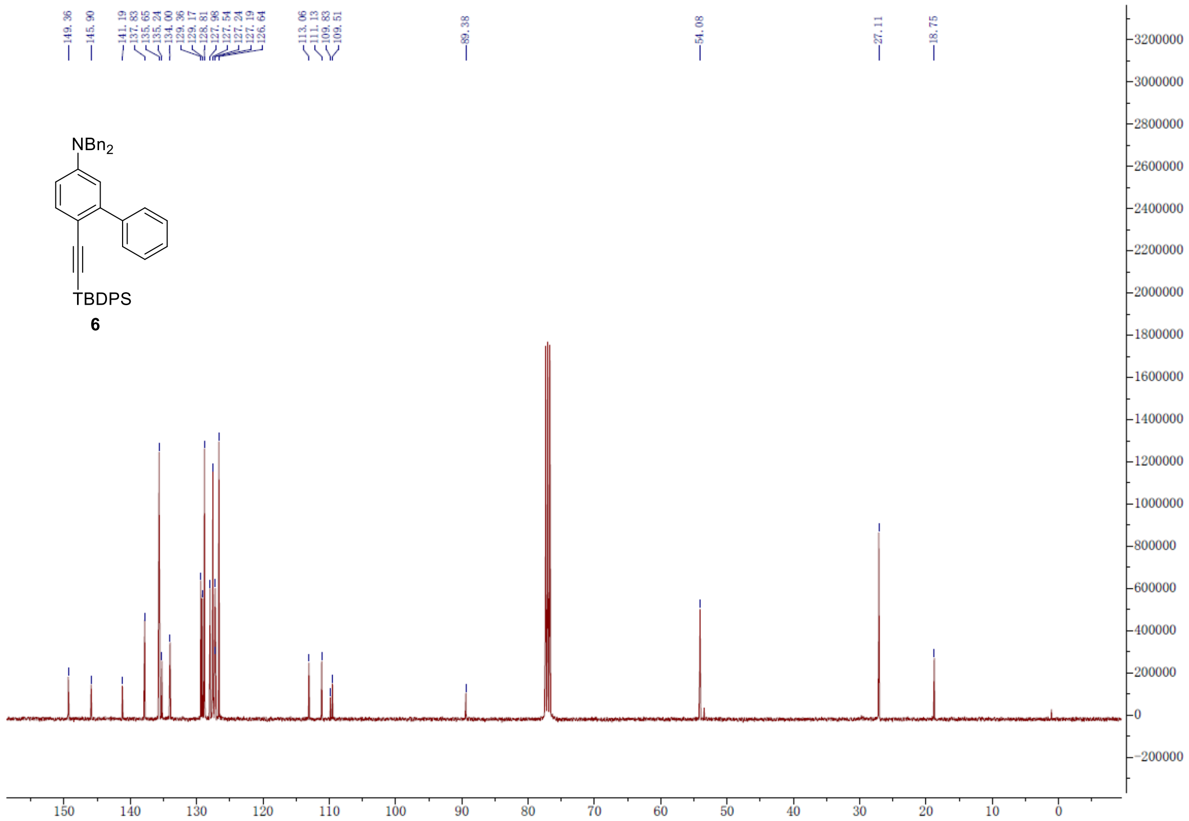
NBn2
TBDPS
6

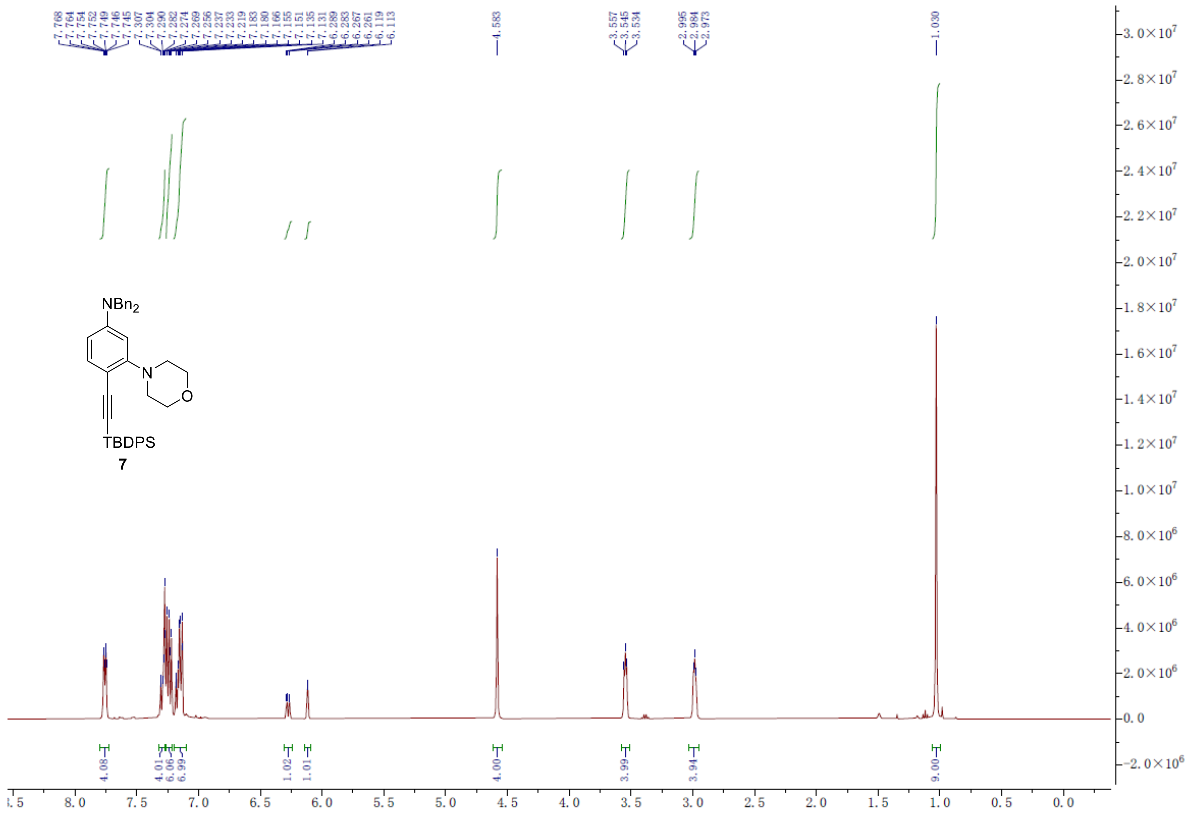

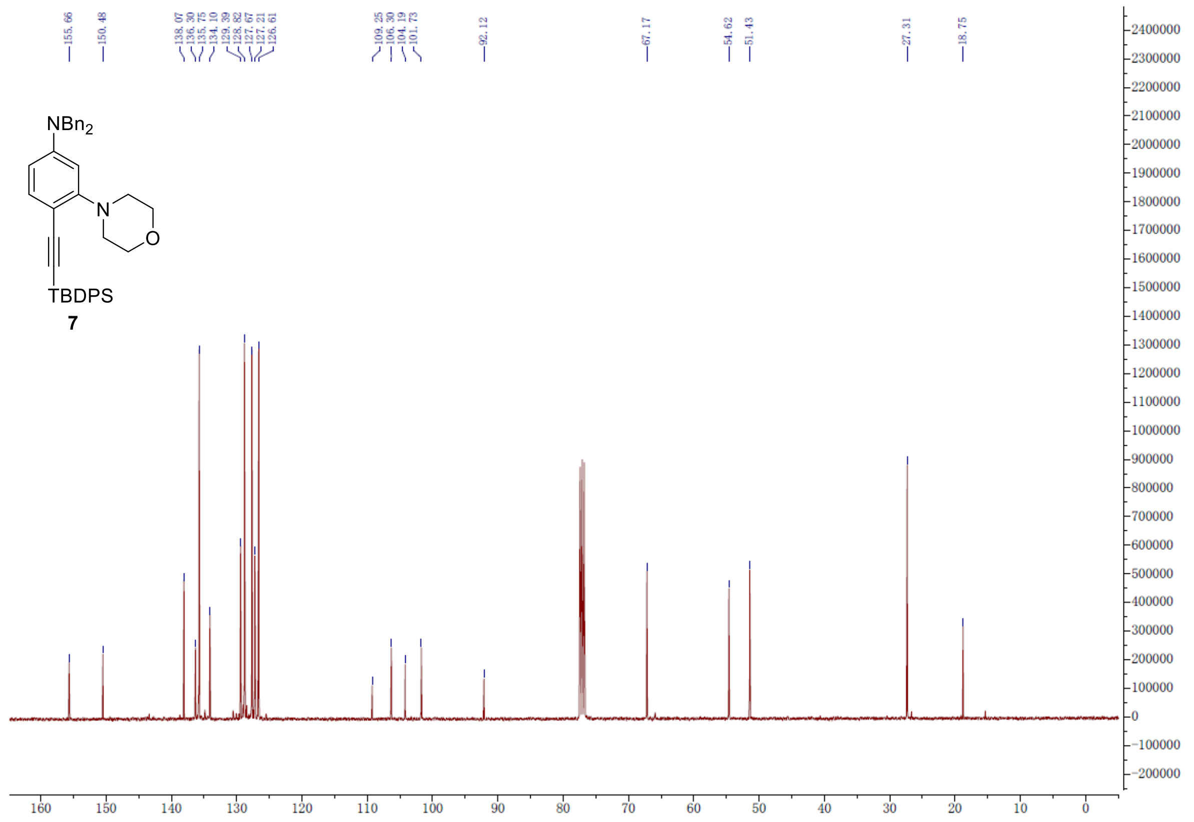

8. References

[1] K. Naksomboon, J. Poater, F. M. Bickelhaupt and M. Á. Fernández-Ibáñez, *J. Am. Chem. Soc.*, 2019, **141**, 6719.

[2] W. L. Jia, N. Westerveld, K. M. Wong, T. Morsch, M. Hakkennes, K. Naksomboon and M. Á. Fernández-Ibáñez, *Org. Lett.*, 2019, **21**, 9339

[3] K. Naksomboon, C. Valderas, M. Gómez-Martínez, Y. Álvarez-Casao and M. Á. Fernández-Ibáñez, *ACS Catal.*, 2017, **7**, 6342.

[4] S. Zhu, A. Das, L. Bui, H. Zhou, D. P. Curran, and M. Rueping, *J. Am. Chem. Soc.*2013, **135**, 1823.

[5] C. B. Singh, V. Kavala, A. K. Samal and B. K. Patel, *Eur. J. Org. Chem.*, **2007**, 1369.

[6] K. Hanaoka, Y. Kagami, W. Piao, T. Myochin, K. Numasawa, Y. Kuriki, T. Ikeno, T. Ueno, T. Komatsu, T. Terai, T. Nagano and Y. Urano, *Chem. Commun.*, 2018, **54**, 6939.

[7] C. B. Jacobsen, M. Meldal, and F. Diness, *Chem. Eur. J.*, 2017, **23**, 846.

[8] X. Kong, H. Zhang, Y. Xiao, C. Cao, Y. Shi and G. Pang, *RSC Adv.*, 2015, **5**, 7035.

[9] B. Xu, M.-L. Li, X.-D. Zuo, S.-F. Zhu, and Q.-L. Zhou, *J. Am. Chem. Soc.*, 2015, **137**, 8700.

[10] C. B. Jacobsen, M. Meldal and F. Diness, *Chem. Eur. J.*, 2017, **23**, 846.

[11] S. Peil, G. Bistoni, R. Goddard and A. Fürstner, *J. Am. Chem. Soc.*, 2020, **142**, 18541.

[12] K. Ishihara, J. Kobayashi, K. Nakano, H. Ishibashi and H. Yamamoto, *Chirality*, 2003, **15**, 135.

[13] H. Abe and H. Suzuki, *Bull. Chem. Soc. Jpn.*, 1999, **72**, 787.

[14] J. Le Shih, S. Jansone-Popova, C. Huynh and J. A. May, *Chem. Sci.*, 2017, **8**, 7132.

[15] Z. Ruan, S. Lackner and L. Ackermann, *ACS Catal.*, 2016, **6**, 4690.

[16] L. D. Caspers, P. Finkbeiner and B. J. Nachtsheim, *Chem. Eur. J.*, 2017, **23**, 2748.

[17] T. Wang, Y. N. Wang, R. Wang and X. S. Wang, *Chem. Asian J.*, 2018, **13**, 2449.

[18] J. Takaya, S. Udagawa, H. Kusama and N. Iwasawa, *Angew. Chem. Int. Ed.*, 2008, **120**, 4984.
